# Nucleobase–Guanidiniocarbonyl-Pyrrole Conjugates as Novel Fluorimetric Sensors for Single Stranded RNA

**DOI:** 10.3390/molecules22122213

**Published:** 2017-12-13

**Authors:** Željka Ban, Biserka Žinić, Robert Vianello, Carsten Schmuck, Ivo Piantanida

**Affiliations:** 1 Division of Organic Chemistry and Biochemistry, Ruđer Bošković Institute, Bijenička cesta 54, 10000 Zagreb, Croatia; Zeljka.Ban@irb.hr (Ž.B.); biserka.zinic@irb.hr (B.Ž.); robert.vianello@irb.hr (R.V.); 2 Institute for Organic Chemistry, University of Duisburg-Essen, Universitätsstrasse 7, 45141 Essen, Germany; carsten.schmuck@uni-due.de

**Keywords:** pyrrolocytosine, guanidiniocarbonyl-pyrrole, DNA/RNA binding, fluorimetric recognition

## Abstract

We demonstrate here for the first time that a guanidiniocarbonyl-pyrrole (GCP) unit can be applied for the fine recognition of single stranded RNA sequences—an intuitively unexpected result since so far binding of the GCP unit to ds-DNA or ds-RNA relied strongly on minor or major groove interactions, as shown in previous work. Two novel nucleobase–GCP isosteric conjugates differing in the flexibility of GCP unit revealed a fluorimetric recognition of various single stranded RNA, which could be additionally regulated by pH. The more rigid conjugate showed a specific fluorescence increase for poly A only at pH 7, whereby this response could be reversibly switched-off at pH 5. The more flexible derivative revealed selective fluorescence quenching by poly G at pH 7 but no change for poly A, whereas its recognition of poly AH^+^ can be switched-on at pH 5. The computational analysis confirmed the important role of the GCP fragment and its protonation states in the sensing of polynucleotides and revealed that it is affected by the intrinsic dynamical features of conjugates themselves. Both conjugates showed a negligible response to uracil and cytosine ss-RNA as well as ds-RNA at pH 7, and only weak interactions with ds-DNA. Thus, nucleobase–GCP conjugates can be considered as novel lead compounds for the design of ss-RNA or ss-DNA selective fluorimetric probes.

## 1. Introduction

Molecular visualization and recognition of DNA and RNA are an important area of ongoing research in medicine and molecular biology, in which small molecules can be not only useful dyes but also effective anticancer, antibiotic and antiviral therapeutic agents [1]. Besides the naturally most abundant ds-DNA, many DNA and RNA structures were discovered (i-motif, triplex and G-quadruplex structures), becoming novel targets for drugs, for instance in cancer chemotherapy [2]. Most of the studies of small molecules were focused on their interactions with double- or multiple-stranded DNA or RNA, but not with single stranded DNA/RNA. That was mostly due to a lack of interest since small molecules generally bind more strongly to multi-stranded nucleic acid structures than to single-stranded (ss) polymers. Moreover, ss-polynucleotides adopt poorly defined secondary structures, making ligand design a very challenging task. Nevertheless, single-stranded nucleic acids such as poly(A) have critical roles in cell biology [3,4,5], while single stranded G-rich structures can fold in a variety of G-quadruplexes. Furthermore, some RNA in the genomes of various cardioviruses and encephalomyocarditis viruses contain stretches composed of more than 75% cytosine, and single stranded C-polymers can adopt a non-B form structure called i-motif under slightly acidic or neutral pH [6,7]. Very few small molecules reveal selective binding to single stranded DNA or RNA structures.

For instance, some isoquinoline alkaloids bind poly(A) with considerable affinity [8,9] as does the the aminoglycoside antibiotic neomycin [10]. The most effective approach to ss-polynucleotide recognition by small molecules (MW < 1000) was combining intercalative unit with a nucleobase, as introduced by Lhomme et al. [11,12], and developed for various ss-polynucleotides by a series of phenanthridinium [13,14,15,16,17] and bis-phenanthridinium-nucleobase conjugates [18,19]. Also, some DBTAA-adenine conjugates specifically recognised poly dT [20].

Our research on novel moieties for interactions with DNA/RNA revealed intriguing properties of the guanidiniocarbonyl-pyrrole system (abbreviated GCP), which we studied in detail on various ds-DNA and ds-RNA. Particularly interesting were aryl-GCP conjugates, which, depending on their structure, showed specific fluorimetric and CD recognition of different ds-DNA or ds-RNA structures depending on pH or steric and/or H-bonding properties of conjugates [21,22,23,24,25]. Mostly, the GCP unit interacted within the DNA or RNA grooves, although there were indications that in its protonated form (pH 5) GCP could interact also with negatively charged DNA/RNA backbone. Particularly latter property raised the question whether GCP unit could also be used for interactions with DNA/RNA targets which do not have a well-defined secondary structure and hence minor or major grooves, respectively.

**Figure 1:**
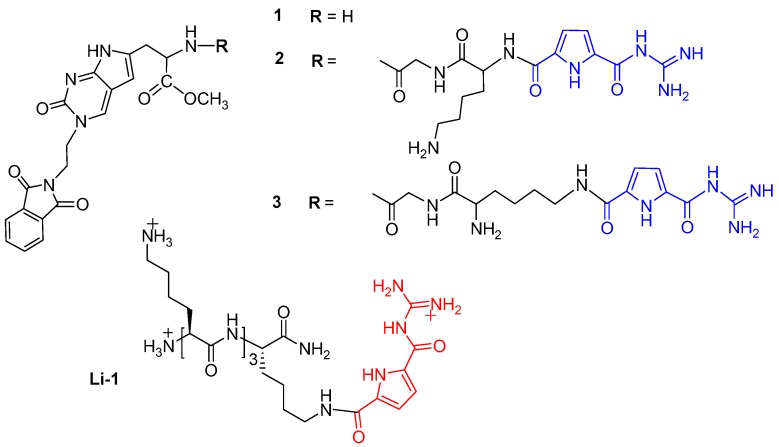
Studied compounds **1**–**3** and previously studied analogue **Li**-**1** [26].

Up till now, we did not assay the interactions of aryl-GCP conjugates with single stranded DNA or RNA. To address this, we relied on our previous research based on condensed-aryl-nucleobase conjugates [13,14,15,16,17,18,19,20] targeting particular ss-polynucleotide sequences, whereby large aryl moiety ensured sufficient DNA/RNA affinity and spectroscopic response while an attached nucleobase contributed to particular ss-sequence recognition by a set of H-bonds. In the research presented here we designed and prepared a novel fluorescent cytosine-amino acid analogue **1** with C- and N-end available for peptide coupling to ensure broad applicability (Figure 1). Further on, we attached GCP on lysine at two different positions and consequently coupled the two different GCP-lysines with **1**. The obtained nucleobase–GCP conjugates are isosteres, differing in GCP position either on the shorter and more rigid alpha-amino position (**2**) or on the longer, more flexible side-chain amino position (**3**).

## 2. Results and Discussion

### 2.1. Chemistry

We have designed and synthesized a new type of molecular probes by combining two molecular fragments Pyrr-C and GCP, both well known for their capability to strongly interact with nucleic acids. The fluorescent pyrrolocytosine nucleobase (Pyrr-C) [27] preserves the H-bonding pattern of the parent nucleobase cytosine [28,29], while the guanidiniocarbonyl-pyrrole (GCP) moiety is known as a highly efficient DNA minor groove binder [23,25].

**Scheme 1:**
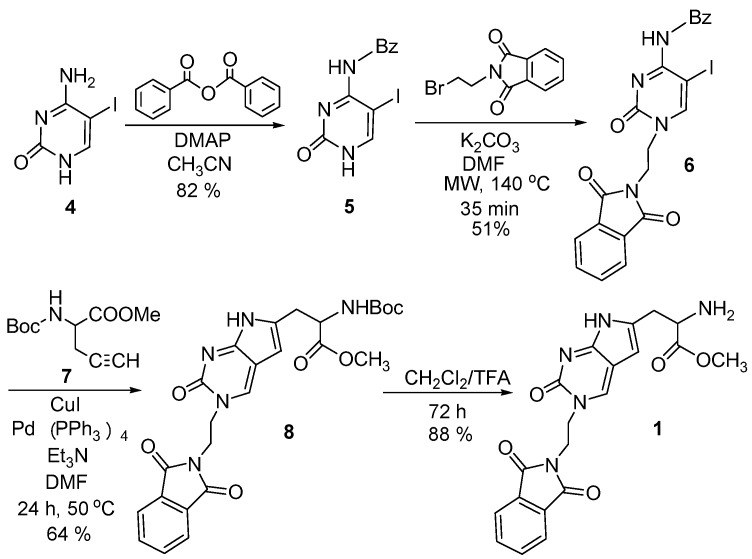
Synthesis of novel fluorescent Pyrr-C-amino acid analogue **1**.

We synthesized the fluorescent Pyrr-C-amino acid analogue **1** (Scheme 1) which served as the starting compound for the preparation of the two novel nucleobase-guanidiniocarbonyl-pyrrole conjugates **2** and **3**. Previous studies have shown that the Sonogashira coupling of terminal alkynes with 5-iodocytosine derivatives gave the 5-alkynyl products while *N*^4^-benzoyl-5-iodocytosine derivatives lead to the annulated pyrrolocytosine derivatives via domino cross-coupling and cyclization reaction [30,31]. 

With these facts in mind, the 5-iodo-*N*^4^-benzoylcytosine **5** was synthesized in a very good yield starting from cytosine (which was previously iodinated by using I_2_/HIO_3_ in biphasic AcOH/H_2_O/CH_2_Cl_2_ yielding 5-iodocytosine **4** [32,33], followed by the reaction with benzoic anhydride and the presence of DMAP in acetonitrile as described earlier for the synthesis of *N*^4^-benzoylcytosine (Scheme 1) [34]. 

As the next step, we planned to introduce N-1 substituent by alkylation of **5** with *N*-(2-bromoethyl)phthalimide. This substituent may have multiple roles; (i) it may simplify the isolation of the subsequent products due to increased lipophilicity; (ii) after removal of the phthalimide group a free amino group is generated enabling further functionalization; and (iii) the phthalimide groups itself could contribute to intermolecular interaction by H-bonding and/or aromatic stacking interactions. The best yield of N-1 alkylated product was obtained by alkylation of **5** with *N*-(2-bromoethyl)phthalimide in the presence of K_2_CO_3_/DMF under microwave irradiation at 140 °C giving **6** in 51% yield. 

Pyrr-C derivative **8** was synthesized by adopting the methods reported in the literature [35]. The one pot sequential Sonogashira cross-coupling reaction and annulation of **6** with protected [36] propargylglycine **7** was used. Compound **8** was readily deprotected by CH_2_Cl_2_/TFA to afford **1** in a very good yield (88%).

**Scheme 2:**
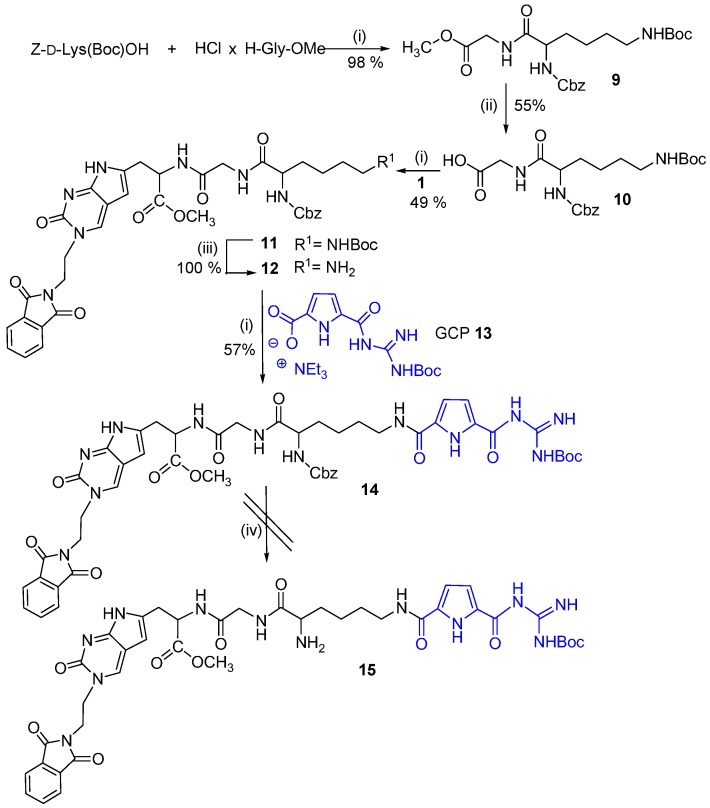
Attempted synthesis of **15**. *Reagents and conditions*: (i) HBTU, HOBt, Et_3_N, CH_3_CN; (ii) 2 M NaOH, dioxane/water (*v*/*v* 3:1); (iii) TFA/CH_2_Cl_2_; (iv) H_2_, Pd/C, MeOH.

Having the fluorescent Pyrr-C-amino acid analogue **1** in hand, we decided to link it with the guanidiniocarbonyl-pyrrole unit (Boc-GCP-OH) [37] **13** through a Gly-Lys dipeptide bridge (Scheme 2). We planned to first attach the fluorescent Pyrr-C unit **1** to Gly-Lys dipeptide and the GCP **13** unit in the second step (Scheme 2). Starting from the commercially available Z-d-Lys(Boc)-OH and H-Gly-OMe hydrochloride, the Z-d-Lys(Boc)-Gly-OMe dipeptide **9** was synthesized in excellent yield by a standard coupling reaction using HBTU/HOBt as coupling reagents and the base, triethylamine in acetonitrile [38]. Dipeptide **9** was readily deprotected with 2 M NaOH in dioxane/H_2_O (*v*/*v* 3:1) giving **10**. The same coupling procedure was applied for coupling of the Pyrr-C amino acid analogue **1** with Z-d-Lys(Boc)-Gly-OH dipeptide **10**; the product **11** is obtained in moderate yield. The Boc-protecting group was removed with a mixture of CH_2_Cl_2_/TFA affording **12** in quantitative yield. In the next step, **12** was coupled with GCP **13** giving the conjugate **14**. Unexpectedly, the removal of Cbz protecting group in **14** by hydrogenolysis failed and no deprotected compound **15** could be isolated.

**Scheme 3:**
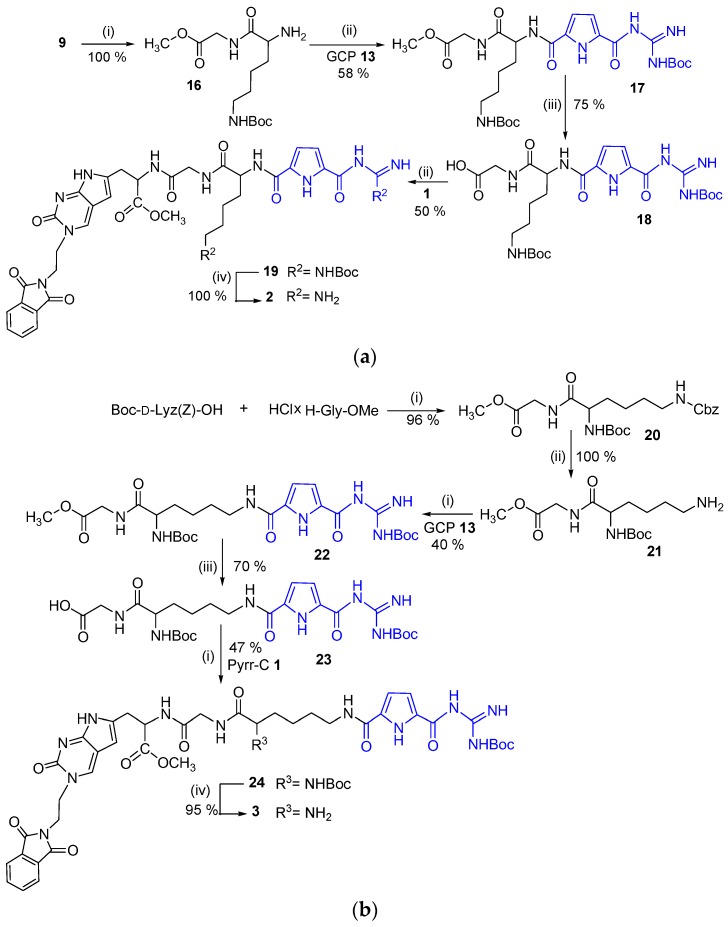
Synthesis of nucleobase–GCP conjugates **2** and **3**. *Reagents and conditions*: (**a**) (i) H_2_, Pd/C, MeOH; (ii) HBTU, HOBt, Et_3_N, CH_3_CN; (iii) LiOH, THF/water (*v*/*v* 4:1); (iv) CH_2_Cl_2_/TFA; (**b**) (i) HBTU, HOBt, Et_3_N, CH_3_CN; (ii) H_2_, Pd/C, MeOH; (iii) LiOH, THF/water (*v*/*v* 4:1); (iv) TFA/CH_2_Cl_2_.

Hence, the synthetic approach was changed in the way that the GCP **13** is firstly attached to Gly-Lys dipeptide at either of the two (alpha-amino or side-chain amino) positions, and the obtained products are subsequently coupled with the fluorescent Pyrr-C unit **1** (Scheme 3a,b). 

The synthesis of novel nucleobase-guanidiniocarbonyl-pyrrole conjugate **2** started by hydrogenolysis of Z-d-Lys(Boc)-Gly-OMe dipeptide **9** which afforded MeO-Gly-H-Lys(Boc)-dipeptide **16** in quantitative yield (Scheme 3a). Using a standard coupling reaction with HBTU/HOBt conjugate **17** was synthesized in moderate yield. The methyl ester was then removed by basic hydrolysis yielding **18**, which was then coupled with Pyrr-C derivative **1** resulting in hybrid compound **19**. Finally desired conjugate **2**, with the GCP-unit attached to the α-amino position of Lys unit, was obtained by deprotection of Boc-protecting groups with CH_2_Cl_2_/TFA. The preparation of nucleobase–GCP conjugate **3** with the GCP-unit attached to the side-chain amino position of Lys unit is outlined in Scheme 3b. In this case, the starting materials were amino acid Boc-d-Lys(Z)-OH and H-Gly-OMe hydrochloride, which were coupled by HBTU/HOBt activation giving the dipeptide **20**. After the Cbz deprotection, **21** was obtained in quantitative yield. Then, **21** was coupled with guanidiniocarbonyl-pyrrole derivative **13** under standard HBTU/HOBt conditions, providing **22** in moderate yield. The methyl ester in the conjugate **22** was easily removed by basic hydrolysis giving the GCP peptide **23** which was then coupled with the Pyrr-C derivative **1** under standard coupling conditions giving the conjugate **24**. Finally, Boc removal from **24** was again carried out with TFA, providing the Pyrr-C-guanidiniocarbonyl-pyrrole derivative **3** in high yield.

### 2.2. Interactions with DNA and RNA

**Figure 2:**
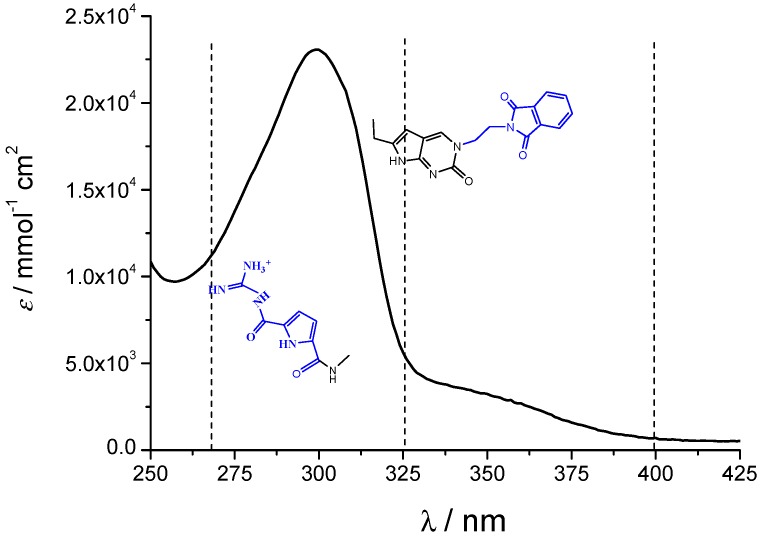
UV/Vis spectrum of **2** at pH 7.0, sodium cacodylate buffer, *I* = 0.05 M, note marked absorption ranges for two chromophores.

All compounds **1**–**3** are moderately soluble in water, and for easier application 0.01 M stock solutions were prepared in DMSO and diluted in buffered solutions prior to use (0.05 M Na cacodylate, DMSO content of the final solutions <0.01%). The UV/Vis spectra (Figure 2) of buffered solutions of **1**–**3** are proportional to their concentrations up to *c* = 2 × 10^−5^ mol dm^−3^, pointing out that studied compounds do not aggregate by intermolecular stacking at experimental conditions used. Since it is known that GCP unit is protonated at weakly acidic conditions (pH 5) [25], UV/Vis spectra were collected at pH 7 and pH 5, but only negligible differences were observed. At pH 7 compounds **1**–**3** bear one positive charge, while at pH 5 the GCP group is also protonated [25], so **2**, **3** have charge +2. Absorption maxima and corresponding molar extinction coefficients (ε) are given in Table S1 (Supplementary Materials). Heating of the aqueous solutions of **1**–**3** up to 90 °C did not cause any significant changes in the UV/Vis spectra and reproducibility upon cooling back to room temperature verified the chemical stability of the compounds.

**Figure 3:**
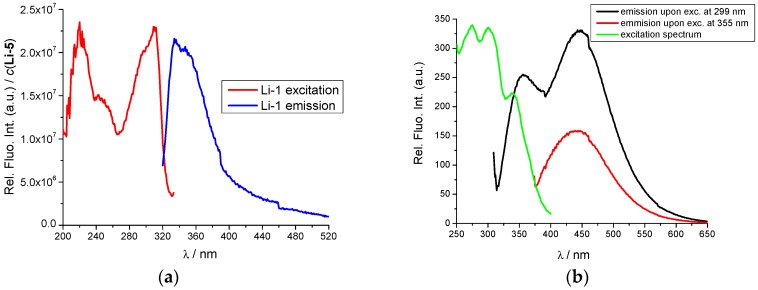
(**a**) Previously reported analogue GCP [26] fluorescence excitation spectrum (λ_em_ = 350 nm) and emission spectrum (λ_exc_ = 299 nm); (**b**) excitation (λ_em_ = 447 nm ▬) and emission (λ_exc_ = 299 nm ▬, λ_exc_ = 355 nm ▬) spectra of 2. Done at pH 7.0, sodium cacodylate buffer, *I* = 0.05 M.

The studied compounds have two fluorophores (GCP and nucleobase) connected by a more (compound **3**) or less (compound **2**) flexible linker. As shown in Figure 2 and Figure 3 (and Figure S12 of Supplementary Materials for reference compound **1**), excitation at λ_exc_ = 299 nm excites all fluorophores, while excitation at λ_exc_ = 355 nm excites only the nucleobase. That allows sequential fluorimetric studies, whereby fluorimetric response upon excitation at λ_exc_ = 299 nm monitors the impact of studied binding to all chromophores, while excitation at λ_exc_ = 355 nm is focused on the changes induced only on nucleobase-phthalimide moiety. 

### 2.3. DNA/RNA Binding Studies

Interactions of **1**–**3** with DNA and RNA were studied at both pH 7 and pH 5, due to the difference in the protonation state of GCP [25]. It should be stressed that poly A and poly C are single stranded under neutral conditions (pH 7), while at pH 5 they become double stranded (poly AH^+^-poly AH^+^ and poly CH^+^-poly CH^+^) [39,19].

With representative ds-DNA (calf thymus DNA) as well as with ds-RNA (poly A–poly U) none of the studied compounds showed any stabilisation in thermal denaturation experiments, at both pH 7 and pH 5 (Figures S46 and S47 of Supplementary Materials). Lack of thermal stabilisation excludes intercalative binding mode [2].

**Figure 4:**
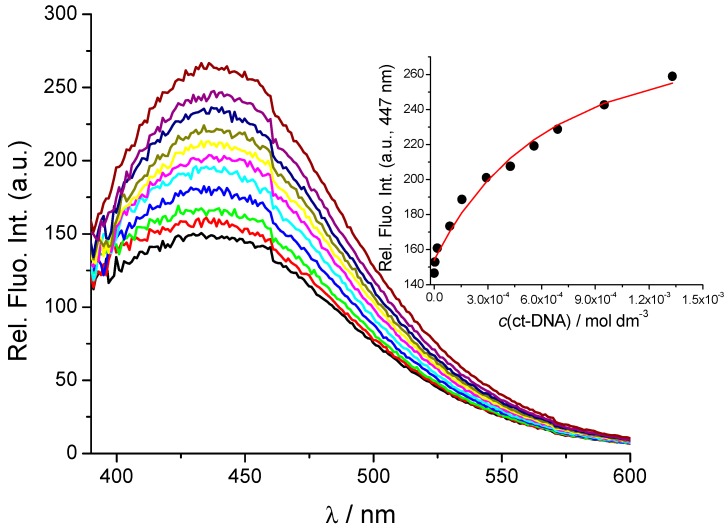
Fluorescence spectra changes of **2** (*c* = 2 × 10^−6^ mol dm^−3^) upon addition of ct-DNA at pH 7.0, sodium cacodylate buffer, *I* = 0.05 M, λ_exc_ = 355 nm. Inset: emission at 447 nm dependence on *c*(DNA) fitted to Scatchard equation [40,41].

Fluorimetric titrations of **2** (Figure 4) and **3** (Figure S29 of Supplementary Materials) at pH 7 revealed significant emission increase only upon addition of ct-DNA, but no emission change upon titration with ds-RNA (poly A–poly U, Figures S19 and S33 of Supplementary Materials). Intriguingly, at pH 5 also poly A–poly U yielded significant increase of fluorescence (Figure S24 of Supplementary Materials), but only for **2**. The lack of binding of **2**, **3** to ds-RNA at pH 7, but efficient binding of **2** at pH 5, could be partially attributed to the protonation of the GCP unit, which as previously shown, can significantly contribute to polynucleotide binding [22,23,24,25]. However, obviously position of GCP unit in the compound is also important for efficient binding, thus only for **2** but not **3** giving measurable fluorimetric changes are observed. Processing of titration data by Scatchard equation [40,41] revealed similar affinities (log *K*_s_ ≈ 4, Table 1) for all ds-DNA and ds-RNA (RNA only at pH 5). 

In order to gain insight into the changes of polynucleotide properties induced by small molecule binding, we have chosen CD spectroscopy as a highly sensitive method for conformational changes in the secondary structure of polynucleotides [42]. In addition, achiral small molecules can eventually acquire induced CD spectrum (ICD) upon binding to polynucleotides, which could give useful information about modes of interaction [43].

All studied compounds are chiral but intensity of their CD spectra in the 230–500 nm range (Figures S42–S45 of Supplementary Materials) is negligible with respect to CD spectra of polynucleotides, allowing accurate correction of CD titrations. Addition of **2** or **3** to ct-DNA or ds-RNA did not significantly change the CD spectrum of the polynucleotide at both pH 7 and pH 5, thus pointing out that no DNA/RNA conformational change happened upon compound binding. Moreover, no ICD bands >300 nm were observed, thus chromophores of **2**, **3** did not become uniformly oriented along polynucleotide chiral axis [43].

The lack of thermal stabilisation, negligible changes in CD experiments and rather low binding constants (log *K*_s_ ≈ 4) suggest that the observed fluorescence changes of **2**, **3** are a consequence of random aggregation of compounds along double stranded helix of polynucleotide, based on non-specific electrostatic interactions of their positive charge with DNA/RNA backbone as well as hydrophobic interactions.

#### Single Stranded (ss)-Polynucleotides

Compounds **2** and **3** contain a fluorescent analogue of cytosine, thus we studied their interactions with ss-polynucleotides. Fluorimetric titrations were performed using sequential excitation properties of **2** and **3**, whereby λ_exc_ = 299 nm excites all fluorophores, while excitation at λ_exc_ = 355 nm excites only nucleobase-phthalimide. It is important to stress that **1** (reference nucleobase unit) did not change emission upon addition of any ss-polynucleotide at λ_exc_ = 355 nm (Figure S40 of Supplementary Materials).

**Figure 5:**
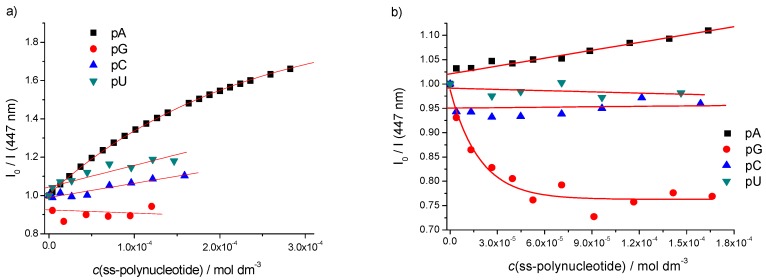
Fluorimetric titrations normalised to starting fluorescence intensity of compound: for **2** (**a**) and **3** (**b**), λ_exc_ = 355 nm, with all ss-polynucleotides. Done at pH 7.0, sodium cacodylate buffer, *I* = 0.05 M.

In general, the addition of any ss-polynucleotide to GCP-rigid analogue **2** yielded indiscriminate emission increase upon excitation at λ_exc_ = 299 nm. However, excitation of **2** at only nucleobase-fluorophore (λ_exc_ = 355 nm) at pH 7 induced significant emission increase of **2** only for poly A (Figure 5a), while intriguingly at pH 5 emission of **2** did not change (Figure S25 of Supplementary Materials), likely due to the formation of poly AH^+^-poly AH^+^ [19,39]. Thus, general fluorescence increase upon λ_exc_ = 299 nm can be attributed to the interaction of GCP-unit, which non-selectively binds to all polynucleotides, while highly selective emission upon λ_exc_ = 355 nm excitation can be attributed to the particular interaction of nucleobase-unit of **2** with poly A (observed only at pH 7).

For GCP-flexible analogue **3**, the excitation at λ_exc_ = 299 nm yielded also fluorescence increase for all ss-RNA. Intriguingly, at pH 7 analogue **3** upon excitation at λ_exc_ = 355 nm showed highly selective emission quenching for poly G in respect to other ss-polynucleotides (Figure 5b), but did not show poly A recognition as **2**. Only at acidic conditions (pH 5) at which GCP is protonated (and poly a forms ds-structure poly AH^+^-poly AH^+^) [39], **3** exhibited the poly A-selective response, whereby intriguingly at the same acidic conditions emission of **2** did not change (Figure S35 of Supplementary Materials). Thus, only **3** yielded emission response upon interacting with poly AH^+^-poly AH^+^.

To examine the influence of GCP-unit on interaction with ss-polynucleotides, we tested interactions of compound **12** (analogue of **2** lacking GCP unit) with poly A (Figure S41 of Supplementary Materials), which at both, pH 7 and pH 5 showed non-selective fluorimetric results, similar to the response of free nucleobase **1** (Pyrr-C). These fluorimetric titrations clearly stressed synergistic effect of GCP conjugation with nucleobase for the ss-polynucleotide recognition by **2** and **3**. All fluorimetric titrations with sufficiently large emission change were processed by means of Scatchard eq. to obtain binding constants (Table 1). 

In CD experiments addition of **2** or **3** did not change significantly the CD spectra of ss-polynucleotides, at both pH 7 and pH 5, thus indicating that no polynucleotide conformational change happened upon compound binding. Moreover, no ICD bands >300 nm were observed, thus chromophores of **2**, **3** upon binding did not become uniformly oriented along polynucleotide chiral axis [43].

### 2.4. Molecular Modelling

The ss-RNA do not have well-defined structural features in aqueous solutions and therefore did not allow accurate modelling approach. However, conjugates **2** and **3**, although isosteres, significantly differ in flexibility of GCP-unit attachment, former being significantly more rigid than latter (Figure 1). Such difference in structure could be responsible for dramatic differences in ss-RNA recognition.

In order to examine conformational features of **2** and **3** and inspect whether their intrinsic dynamics in aqueous solution play any role in determining their ability to interact with biological systems studied here, we performed molecular dynamics (MD) simulations of different protonation forms of **2** and **3** placed in the explicit water solvation followed by the quantum-mechanical analysis at the DFT level with implicit solvation. 

**Scheme 4:**
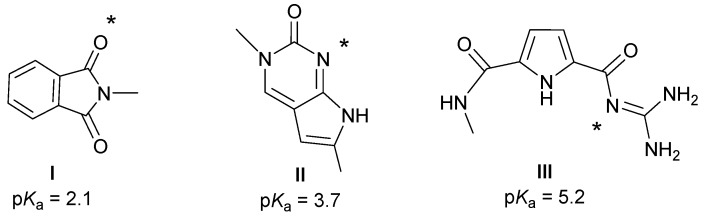
Constituting fragments of systems **2** and **3** and the p*K*_a_ values calculated at the MP2/6–311++G(2df,2pd)//(SMD)/M06–2X/6–31+G(d) level of theory. The most favourable protonation atom is denoted with an asterisk. * represents the most basic position within a molecule.

Initially, we performed the analysis of the acid/base features of **2** and **3** in water to determine their predominant protonation forms under experimental conditions (pH values of 5 and 7). For that purpose, we calculated aqueous phase p*K*_a_ values of fragments **I**–**III** (Scheme 4) that are building blocks of compounds **2**–**3**. In doing so, we assumed that the free amino group originating from lysine will be protonated at both pH values since in lysine its p*K*_a_ value is 10.5, which is unlikely to change much or be reduced by 3–4 p*K*_a_ units in cases studied here [44].

The p*K*_a_ values calculated for **I** and **II** are slightly lower than those of succinimide (p*K*_a_ = 3.9) and cytosine (p*K*_a_ = 4.5) used as references for their evaluation. This implies that even at pH = 5 both of these fragments will predominantly be unprotonated. Interestingly, although guanidine (p*K*_a_ = 13.6) and its derivatives provide some of the strongest organic bases know today [45,46,47], combining it to the carbonyl-pyrrole fragment significantly reduces its basicity to p*K*_a_(**III**) = 5.2, being a consequence of an extended conjugated π-system, which depletes the electron density from the guanidine imino nitrogen leading to much-reduced basicity. This suggests that at pH = 7, the fragment **III** will be neutral, while at pH = 5 it will assume monoprotonated form **III**^+^, thus putting our results in firm agreement with earlier experimental observations [22], and confirming that this fragment is predominantly responsible for the biological activity investigated here.

**Figure 6:**
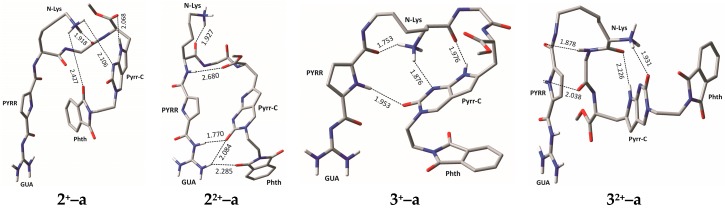
Most stable structures of **2** and **3** in their mono- and di-protonated forms. These are identified after a set of structures, elucidated with the clustering analysis of the corresponding MD trajectories, was optimized at the (SMD)/M06–2X/6–31G(d) level of theory. A larger set of representative structures is placed in the Supplementary Materials (Figures S48–S51). Abbreviations GUA, PYRR, N-Lys, Pyrr-C and Phth denote the guanidine group, pyrrole moiety, free amino group, nucleobase, and phthalimide moiety, respectively. Only selected hydrogen atoms are depicted due to clarity.

Having this in mind, we prepared the geometries of monocationic **2**^+^ and **3**^+^ (protonated at the free amino group, corresponding to pH = 7), as well as dicationic **2**^2+^ and **3**^2+^ (also protonated at the guanidine moiety, corresponding to pH = 5), and submitted all four systems to MD simulations. Following the clustering analysis of the obtained trajectories, we optimized a set of representative structures in each case at the (SMD)/M06–2X/6–31G(d) level of theory and extracted total electronic energies in search of most stable, thus most dominant structures in solution (Figure 6).

It is interesting to observe that, at neutral pH, **2** reveals notable interactions only with ss-poly A, while, surprisingly, at pH = 5, no interactions are detected with either of the ss-polynucleotides. Under acidic conditions, the most stable structure **2^2+^–a** has the guanidine fragment engaged in three rather strong hydrogen bonds involving one of its amino groups interacting with the carbonyl moiety on the nucleobase (1.77 Å), and the other amino group interacting both with the same carbonyl fragment (2.08 Å) and the carbonyl centre from phthalimide (2.29 Å). This is the only case in all four studied systems where, in the most dominant structure, the guanidine moiety is so strongly involved in the intramolecular stabilization, which is a pattern also observed in several other structures subsequently less stable than **2^2+^–a** (Figure S49 of Supplementary Materials). 

This clearly diminishes the ability of the guanidine group in **2^2+^** to interact with other systems as revealed at pH = 5 here. Let us also mention that the most stable 2**^2+^** structure having the guanidine group not involved in any intramolecular hydrogen bonding, **2^2+^–e** (Figure S49 of Supplementary Materials), is 2.9 kcal/mol less stable than **2^2+^–a**, which turns out to be too high to be overcome and provides evidence to no activity of **2** towards any of the ss-polynucleotides at pH =5.

At pH = 7, system **2** loses an extra proton on the guanidine moiety and is present as monocationic **2^+^**. In the most stable structure **2^+^–a** the guanidine moiety is not involved in any hydrogen bonding, which obviously allows **2^+^** to interact with ss-poly A. The intramolecular stabilization in **2^+^–a** predominantly occurs through the interaction of the protonated free amino group with (a) the imino nitrogen on the nucleobase (2.11 Å); (b) the carbonyl group on phthalimide (2.43 Å); and (c) the carbonyl moiety in the amide fragment (1.92 Å). Interestingly, only 0.6 kcal mol^−1^ above **2^+^–a**, a structure **2^+^–b** is also dominated by the intramolecular interactions with the protonated free amino group, but there the guanidine fragment is found in the stabilizing π–π stacking interactions with phthalimide (Figure S48 of Supplementary Materials), which likely affects its interactions with ss-polynucleotides other than ss-poly A. The structure with a non-interacting cytosine nucleobase is 9.6 kcal mol^−1^ less stable than **2^+^–a**, which is clearly too high to have any impact on the potential pairing with ss-poly G. 

Under acidic conditions (pH = 5), system **3** is the only molecule that exhibits interactions with two polynucleotides, namely, poly A and poly G (Table 1). In its most stable structure **3^2+^–a** the guanidine moiety is not involved in any hydrogen bonding, although the nearby pyrrole fragment and vicinal carbonyl group interact with the neighbouring amide fragments at 2.04 and 1.88 Å, respectively. The structure where the whole GCP fragment is free of any hydrogen bonds is **3^2+^–b** (Figure S51 of Supplementary Materials), which is 2.4 kcal mol^−1^ less stable than **3^2+^–a**. Taken all together, all of this allows **3^2+^** to interact with both poly A and poly G, yet relative changes in increasing or decreasing the fluorescence upon binding are the smallest here (Table 1).

Under physiological conditions (pH = 7, system **3^+^**), the situation with **3** is similar to that with **2^+^** in a way that it shows notable interactions with only one polynucleotide, namely ss-poly G (Table 1). The most stable structure **3^+^–a** has the guanidine moiety free of any hydrogen bonding interactions, yet, as in **3^2+^–a**, the interactions of the pyrrole and amide carbonyl of the GCP fragment with the nucleobase carbonyl (1.95 Å) and the protonated free amino group (1.75 Å), respectively, are evident. The structure with the whole GCP fragment deprived of intramolecular interactions is here only 1.9 kcal/mol above the most stable structure, which is likely responsible for slightly higher log *K*_s_ = 5 with **3^+^** compared to log *K*_s_ = 4 with **3^2+^**.

In concluding this section, let us mention that the computational results aided in the interpretation of the relative trends in the observed reactivities of **2** and **3** towards ss-polynucleotides. Since the conformational analysis revealed that in all cases there is not a single structure below 5–6 kcal mol^−1^ having nucleobase fragment not interacting intramolecularly with the rest of the system, this leads us to conclude that in the investigated cases nucleobase moiety is less important for the interaction with ss-polynucleotides. As such, this analysis underlined the important role of the GCP fragment, particularly its guanidine moiety, in successful polynucleotide recognition. It turned out that lowering the pH conditions promotes the interactions with ss-polynucleotides, since protonated guanidine group interacts better, being in agreement with previous experiments [21,22,23,24,25]. Yet, in a more rigid system, this effect is overshadowed by the propensity of **2^2+^** to form strong charge-dipole intramolecular interactions with protonated guanidine group, therefore hindering its ability to interact with other systems. This is why **2^+^** outnumbers **2^2+^** in intermolecular interactions.

## 3. Materials and Methods

### 3.1. General Information

Solvents were distilled from appropriate drying agents shortly before use. TLC was carried out on DC-plastikfolien Kieselgel 60 F254 and preparative thick layer (2 mm) chromatography was done on Merck 60 F254 plates (Merck KGaA, Darmstadt, Germany). (Merck, Merck KGaA, Darmstadt, Germany). NMR spectra were recorded on AV600 and AV300 MHz spectrometers (Bruker BioSpin GmbH, Rheinstetten, Germany), operating at 150.92 or 75.47 MHz for ^13^C and 600.13 or 300.13 MHz for ^1^H nuclei using DMSO-*d*_6_ as the internal standard. Mass spectrometry was performed on the Agilent 6410 Triple Quad mass spectrometer (Agilent Technologies, Santa Clara, CA, USA) and high resolution mass spectra (HRMS) were obtained using a Q-Tof2 hybrid quadrupole time-of-flight mass spectrometer (Micromass, Cary, NC, USA). The electronic absorption spectra were obtained on a Cary 100 Bio spectrometer and CD spectra on a JASCO J815 spectrophotometer (JASCO, Oklahoma City, OK, USA), all in quartz cuvettes (1 cm). The spectroscopic studies were performed in aqueous buffer solution (pH 7.0 and pH 5.0, sodium cacodylate buffer, *I* = 0.05 mol dm^−3^). Under the experimental conditions absorbance of **2** and **3** were proportional to its concentration. Polynucleotides were dissolved in sodium cacodylate buffer, *I* = 0.05 mol dm^−3^, pH = 7. Calf thymus (ct-)DNA was additionally sonicated and filtered through a 0.45 µm filter [48,49]. Polynucleotide concentration was determined spectroscopically as the concentration of phosphates. Spectroscopic titrations were performed at pH 7.0 and pH 5.0 (*I* = 0.05 mol dm^−3^, sodium cacodylate buffer) by adding portions of polynucleotide solution into the solution of the studied compound for UV/Vis experiments and for CD experiments were done by adding portions of compound stock solution into the solution of polynucleotide. Titration data were processed by Scatchard equation. Values for *K*_s_ and *n* given in Table 1 all have satisfactory correlation coefficients (>0.999). Thermal melting curves for DNA and their complexes with studied compounds were determined as previously described [50] by following the absorption change at 260 nm as a function of temperature. Absorbance of the ligands was subtracted from every curve, and the absorbance scale was normalized. The *T*m values are the midpoints of the transition curves, determined from the maximum of the first derivative and checked graphically by the tangent method. ∆*T*m values were calculated subtracting *T*m of the free nucleic acid from *T*m of the complex. Every ∆*T*m value here reported was the average of at least two measurements, the error in ∆*T*m is ± 0.5 °C.

### 3.2. Synthesis

*N*^4^*-Benzoyl-5-iodocytosine* **5**: To a suspension of 5-iodocytosine **4** (2.9 g, 12.24 mmol) and DMAP (196 mg, 1.6 mmol) in anhydrous CH_3_CN (26.2 mL) at room temperature, benzoic anhydride (3.05 g, 13.46 mmol) was added. The reaction mixture was heated to reflux for 18 h and then EtOH (50 mL) was added dropwise. After standing at room temperature overnight, the solid was filtered off, washed with EtOH (3 × 20 mL) dried in vacuo to afford **5** (3.42 g, 82%) as a white solid: ^1^H-NMR (300 MHz, DMSO-*d*_6_) *δ*/ppm: 12.87 (s, 1H, NH-4), 11.93 (br. s, 1H, NH-1), 8.24 (d, *J* = 6.4 Hz, 2H, Bz), 8.17 (s, 1H, H-6), 7.61 (m, 1H, Bz), 7.51 (m, 2H, Bz); ^13^C-NMR (151 MHz, DMSO-*d*_6_) *δ*/ppm: 178.1 (C=O, Bz), 158.3 (C-2), 149.9 (C-6), 148.1 (C-4), 136.6 (Bz), 132.6 (Bz), 129.4 (Bz), 128.3 (Bz), 67.6 (C-5). ESI-MS: *m*/*z* calcd. for C_11_H_7_IN_3_O_2_ [M − H]^−^ 339.96, found 339.80.

*N*^4^*-Benzoyl-1-(2-phthalimidoethyl)-5-iodocytosine* **6**: *N*^4^-benzoyl-5-iodocytosine **5** (198 mg, 0.58 mmol), potassium carbonate (80.1 mg, 0.58 mmol) and *N*-(2-bromoethyl)phthalimide (294.6 mg, 1.16 mmol) and dry DMF (12 mL) were put in a 20 mL glass vial equipped with a small magnetic stirring bar. Then the mixture was put into the cavity of the MW reactor (Monowave 3000, Anton Paar^®^ GmbH, A-8054 Graz, Austria) and irradiated at 140 °C for 35 min. After evaporation of the solvent, CH_2_Cl_2_ and MeOH were added and the resulting solid was filtered off. The filtrate was evaporated and the residue was purified by preparative chromatography (hexane/ethyl acetate 1:1) to give 152 mg (51%) of yellow powder **6**: ^1^H-NMR (300 MHz, DMSO-*d*_6_) *δ*/ppm: 12.87 (br. s, 1H, NH-4), 8.41 (s, 1H, H-6), 8.14 (br. s, 2H, Bz), 7.90–7.82 (m, 4H, Pht), 7.58–7.46 (m, 3H, Bz), 4.05–3.95 (br. s, 2H, CH_2_Pht), 3.94–3.86 (br. s, 2H, CH_2_Pht); ^13^C-NMR (75 MHz, DMSO-*d*_6_) *δ*/ppm: 173.8 (C=O, Bz), 167.7 (C=O, Pht), 163.9 (C-2), 155.9 (C-4), 151.8 (C-6), 137.9 (Bz), 134.4 (Pht), 134.3 (Bz), 131.6 (Pht), 129.2 (Bz), 128.0 (Bz), 123.1 (Pht), 59.9 (C-5), 47.5 (CH_2_Pht), 36.6 (CH_2_Pht). ESI-MS: *m*/*z* calcd. for C_21_H_14_IN_4_O_4_ [M − H]^−^ 513.01, found 512.60.

*Pyrr-C Amino acid analogue* **8**: To a solution of *N*^4^-benzoyl-1-(2-phtalimidoethyl)-5-iodocytosine **6** (40 mg, 0.078 mmol) in anhydrous DMF (546 µL), CuI (3 mg, 0.016 mmol), Et_3_N (22 µL, 0.156 mmol), protected propargylglycine **7** (53.2 mg, 0.234 mmol) and Pd(PPh_3_)_4_ (9 mg, 0.008 mmol) were sequentially added. The reaction mixture was stirred in the dark at 50 °C for 24 h after which the solvent was removed under vacuum. The residue was purified by preparative chromatography (CH_2_Cl_2_/CH_3_OH 9:1) to give the product **8** (25 mg, 64%) as a yellow powder: ^1^H-NMR (300 MHz, DMSO-*d*_6_) *δ*/ppm: 10.99 (s, 1H, NH-7), 8.27 (s, 1H, H-4), 7.81 (s, 4H, Pht), 7.32 (d, *J* = 8.0 Hz, 1H, NH), 5.85 (s, 1H, H-5), 4.38–4.30 (m, 1H, CHCH_2_), 4.17–4.11 (m, 2H, CH_2_Pht), 3.95–3.92 (m, 2H, CH_2_Pht), 3.60 (s, 3H, OCH_3_), 2.97–2.73 (m, 2H, CH_2_CH), 1.38–1.23 (m, 9H, C(CH_3_)_3_); ^13^C-NMR (75 MHz, DMSO-*d*_6_) *δ*/ppm: 172.2 (C=O), 167.7 (C=O, Pht), 159.5 (C-2), 155.3 (C=O), 154.7 (C-6), 140.3 (C-4), 137.2 (C-7a), 134.3 (Pht), 131.6 (Pht), 123.1 (Pht), 108.6 (C-4a), 98.1 (C-5), 78.4 (C(CH_3_)_3_), 52.5 (CHCH_2_), 51.9 (OCH_3_), 49.3 (CH_2_Pht), 37.0 (CH_2_Pht), 29.4 (CH_2_CH), 28.1 (C(CH_3_)_3_). ESI-MS: *m*/*z* calcd. for C_25_H_28_N_5_O_7_ [M + H]^+^ 510.20, found 510.30.

*Pyrr-C Amino acid analogue* **1**: Compound **8** (160 mg, 0.314 mmol) was dissolved in a 1:1 mixture of TFA/CH_2_Cl_2_ (4 mL) and stirred at room temperature for 72 h. After evaporation of volatiles, the residue was purified by preparative chromatography (CH_2_Cl_2_/MeOH 9:1). Product **1** (144 mg, 88%) was obtained as a yellow foam: ^1^H-NMR (300 MHz, DMSO-*d*_6_) *δ*/ppm: 10.94 (br. s, 1H, NH-7), 8.24 (s, 1H, H-4), 7.82 (s, 4H, Pht), 7.70–7.31 (m, 2H, NH_2_), 5.84 (s, 1H, H-5), 4.14–4.12 (m, 2H, CH_2_Pht), 3.95–3.93 (m, 2H, CH_2_Pht), 3.74–3.65 (m, 1H, CHCH_2_), 3.58 (s, 3H, OCH_3_), 2.89–2.69 (m, 2H, CH_2_CH); ^13^C-NMR (75 MHz, DMSO-*d*_6_) *δ*/ppm: 175.1 (C=O), 167.7 (C=O), 159.5 (C-2), 154.8 (C-6), 140.0 (C-4), 138.2 (C-7a), 134.3 (Pht), 131.6 (Pht), 123.1 (Pht), 108.7 (C-4a), 97.7 (C-5), 53.5 (CHCH_2_), 51.5 (OCH_3_), 49.3 (CH_2_Pht), 37.0 (CH_2_Pht), 33.2 (CH_2_CH). HRMS (MALDI-TOF/TOF): *m*/*z* calcd. for C_20_H_20_N_5_O_5_ [M]^+^ 410.1464, found 410.1458.

*Z-d-Lys(Boc)-Gly-OMe* **9**: Z-d-Lys(Boc)OH (300 mg, 0.789 mmol) and H-Gly-OMe hydrochloride (98.6 mg, 0.789 mmol) were dissolved in dry CH_3_CN (10 mL) under argon and HOBt (105.4 mg, 0.789 mmol), HBTU (295.8 mg, 0.789 mmol) and dry Et_3_N (435 µL, 3.16 mmol) were added. The reaction mixture was stirred at room temperature overnight. Product **9** (348 mg, 98%) was isolated by preparative chromatography (CH_2_Cl_2_/CH_3_OH 9:1) as a white powder: ^1^H-NMR (300 MHz, DMSO-*d*_6_) *δ*/ppm: 8.31 (t, *J* = 5.8 Hz, 1H, NH), 7.40–7.29 (m, 6H, Ph, NH), 6.75 (t, *J* = 5.1 Hz, 1H, NH), 5.02 (s, 2H, OCH_2_Ph), 4.02–3.95 (m, 1H, CHCH_2_), 3.92–3.75 (m, 2H, COCH_2_NH), 3.62 (s, 3H, OCH_3_), 2.91–2.84 (m, 2H, CH_2_NH), 1.68–1.49 (m, 2H, CH_2_CH), 1.46–1.23 (m, 13H, 2 × CH_2_, C(CH_3_)_3_); ^13^C-NMR (75 MHz, DMSO-*d*_6_) *δ*/ppm: 172.6 (C=O), 170.2 (C=O), 156.0 (C=O, Cbz), 155.6 (C=O, Boc), 137.0 (Ph), 128.3 (Ph), 127.8 (Ph), 127.7 (Ph), 77.4 (C(CH_3_)_3_), 65.4 (OCH_2_Ph), 54.5 (CHCH_2_), 51.7 (OCH_3_), 40.5 (COCH_2_NH), 39.8 (CH_2_NH), 31.6 (CH_2_CH), 29.2 (CH_2_), 28.3 (C(CH_3_)_3_), 22.7 (CH_2_). ESI-MS: *m*/*z* calcd. for C_22_H_33_N_3_NaO_7_ [M + Na]^+^ 474.22, found 474.22. 

*Z-d-Lys(Boc)-Gly-OH* **10**: Compound **9** (170 mg, 0.377 mmol) was dissolved in a 3:1 mixture of dioxane/water (4 mL), 2M NaOH (470 µL, 0.942 mmol) was added and reaction mixture was stirred at room temperature for 24 h. The solvent was evaporated, the residue was dissolved in water (~20 mL) and acidified with 1 M HCl to pH 5. The product was extracted with EtOAc (5 × 20 mL) dried over Na_2_SO_4_ yielding **10** (90 mg, 55%) as a colourless soil: ^1^H-NMR (600 MHz, DMSO-*d*_6_) *δ*/ppm: 7.55 (d, *J* = 8.1 Hz, 1H, NH), 7.37–7.23 (m, 6H, Ph, NH), 6.75–6.73 (m, 1H, NH), 5.03 (q, *J* = 12.6 Hz, 2H, OCH_2_Ph), 3.90–3.86 (m, 1H, CHCH_2_), 3.25–3.21 (m, 2H, COCH_2_NH), 2.89–2.83 (m, 2H, CH_2_NH), 1.64–1.24 (m, 15H, 3 × CH_2_, C(CH_3_)_3_); ^13^C-NMR (75 MHz, DMSO-*d*_6_) *δ*/ppm: 170.6 (C=O), 170.0 (C=O), 156.1 (C=O, Cbz), 155.6 (C=O, Boc), 137.1 (Ph), 128.4 (Ph), 127.7 (Ph), 127.6 (Ph), 77.3 (C(CH_3_)_3_), 65.3 (OCH_2_Ph), 55.1 (CHCH_2_), 44.1 (COCH_2_NH), 39.9 (CH_2_NH), 31.4 (CH_2_CH), 29.1 (CH_2_), 28.3 (C(CH_3_)_3_), 22.8 (CH_2_). ESI-MS: *m*/*z* calcd. for C_21_H_30_N_3_O_7_ [M − H]^−^ 436.21, found 436.0. 

*Pyrr-C Tripeptide* **11**: Pyrr-C amino acid analogue **1** (46 mg, 0.088 mmol) and dipeptide **10** (38.4 mg, 0.088 mmol) were dissolved in dry CH_3_CN (5 mL) under argon and HOBt (11.9 mg, 0.088 mmol), HBTU (33.3 mg, 0.088 mmol) and dry Et_3_N (49 µL, 0.352 mmol) were added. Reaction was stirred at room temperature overnight. Product **11** (36 mg, 49%) was isolated by preparative chromatography (CH_2_Cl_2_/CH_3_OH 9:1) as a yellow powder: ^1^H-NMR (300 MHz, DMSO-*d*_6_) *δ*/ppm: 11.03 (br. s, 1H, NH-7), 8.34–8.32 (m, 1H, NH), 8.26 (s, 1H, H-4), 8.17–8.12 (m, 1H, NH), 7.81 (s, 4H, Pht), 7.48–7.42 (m, 1H, NH), 7.35–7.26 (m, 5H, Ph), 6.75 (t, *J* = 5.6 Hz, 1H, NH), 5.87 (s, 1H, H-5), 5.05–4.95 (m, 2H, OCH_2_Ph), 4.62 (br. s, 1H, CHCH_2_), 4.20–4.08 (m, 2H, CH_2_ Pht), 4.00–3.89 (m, 3H, CHCH_2_, CH_2_Pht), 3.74–3.65 (m, 2H, COCH_2_NH), 3.58 (s, 3H, OCH_3_) 2.97–2.85 (m, 4H, CH_2_CH, CH_2_NH), 1.62–1.49 (m, 2H, CH_2_CH), 1.44–1.16 (m, 15H, 3 × CH_2_, C(CH_3_)_3_); ^13^C-NMR (151 MHz, DMSO-*d*_6_) *δ*/ppm: 172.3 (C=O), 171.4 (C=O), 168.8 (C=O), 167.7 (C=O), 159.5 (C-2), 156.1 (C=O, Cbz), 155.6 (C=O, Boc), 154.8 (C-6), 140.3 (C-4), 139.0 (C-7a), 136.8 (Ph), 134.3 (Pht), 131.6 (Pht), 128.3 (Ph), 127.8 (Ph), 127.7 (Ph), 123.1 (Pht), 108.6 (C-4a), 98.1 (C-5), 77.3 (C(CH_3_)_3_), 65.4 (OCH_2_Ph), 54.7 (CHCH_2_), 52.0 (OCH_3_), 51.2 (CHCH_2_), 49.3 (CH_2_Pht), 41.6 (COCH_2_NH), 39.8 (CH_2_NH), 37.0 (CH_2_Pht), 31.4 (CH_2_CH), 29.6 (CH_2_), 29.2 (CH_2_), 28.3 (C(CH_3_)_3_), 22.8 (CH_2_). ESI-MS: *m*/*z* calcd. for C_41_H_48_N_8_NaO_11_[M + Na]^+^ 851.33, found 851.50.

*Pyrr-C Tripeptide* **12**: Compound **11** (160 mg, 0.193 mmol) was dissolved in 1:1 mixture of TFA/CH_2_Cl_2_ (4 mL) and stirred at room temperature for 20 h. After removal of remaining TFA under reduced pressure, product **12** (162.7 mg, 100%) was obtained as a yellow foam: ^1^H-NMR (300 MHz, DMSO-*d*_6_) *δ*/ppm: 11.19 (br. s, 1H, NH-7), 8.38–8.35 (m, 2H, NH, H-4), 8.19–8.14 (m, 1H, NH), 7.81 (s, 4H, Pht), 7.63 (br. s, 3H, NH_3_), 7.50–7.46 (m, 1H, NH), 7.36–7.31 (m, 5H, Ph), 5.92 (s, 1H, H-5), 5.04–4.97 (m, 2H, OCH_2_Ph), 4.64–4.61 (m, 1H, CHCH_2_), 4.20–3.59 (m, 10H, 2 × CH_2_Pht, CHCH_2_, COCH_2_NH, OCH_3_ + H_2_O), 3.02–2.86 (m, 2H, CH_2_CH), 2.74 (br. s, 2H, CH_2_), 1.62–1.17 (m, 6H, 3 × CH_2_); ^13^C-NMR (151 MHz, DMSO-*d*_6_) *δ*/ppm: 172.5 (C=O), 172.4 (C=O), 172.4 (C=O), 168.0 (C=O), 159.8 (C-2), 158.5, 158.4, 158.3, 157.8 (4 × CF_3_C=O), 156.4 (C=O, Cbz), 154.5 (C-6), 140.8 (C-4), 139.9 (C-7a), 137.3 (Ph), 134.6 (Pht), 131.7 (Pht), 128.6 (Ph), 127.9 (Ph), 123.3 (Pht), 119.7 (CF_3_C=O), 109.2 (C-4a), 98.9 (C-5), 66.0 (OCH_2_Ph), 52.4 (CHCH_2_), 51.53 (OCH_3_), 51.51 (CHCH_2_), 49.7 (CH_2_Pht), 40.2 (COCH_2_NH), 38.9 (CH_2_NH), 37.2 (CH_2_Pht), 31.2 (CH_2_CH), 29.9 (CH_2_), 26.7 (CH_2_), 22.4 (CH_2_). ESI-MS: *m*/*z* calcd. for C_36_H_41_N_8_O_9_ [M]^+^ 729.3, found 729.3.

*Pyrr-C Tripeptide GCP conjugate* **14**: Compound **12** (30 mg, 0.035 mmol) and Boc-GCP-OH **13** (14.1 mg, 0.035 mmol) were dissolved in dry CH_3_CN (3 mL) under argon and HOBt (4.7 mg, 0.035 mmol), HBTU (13.3 mg, 0.035 mmol) and dry Et_3_N (19 µL, 0.14 mmol) were added. Reaction was stirred at room temperature overnight. Product **14** (20 mg, 57%) was isolated by preparative chromatography (CH_2_Cl_2_/CH_3_OH 9:1) as a yellow powder: ^1^H-NMR (300 MHz, DMSO-*d*_6_) *δ*/ppm: 11.03 (m, 2H, NH-7, NH), 9.27 (br. s, 2H, 2 × NH), 8.59–8.42 (m, 1H, NH), 8.40–8.29 (m, 1H, NH), 8.27 (s, 1H, H-4), 8.18 (m, 1H, NH), 7.80 (s, 4H, Pht), 7.50 (m, 1H, NH), 7.34–7.20 (m, 6H, Ph, NH), 6.79–6.67 (m, 2H, CH-Pyrr), 5.87 (s, 1H, H-5), 5.03–4.96 (m, 2H, OCH_2_Ph), 4.63–4.59 (m, 1H, CHCH_2_), 4.18–4.09 (m, 2H, CH_2_Pht), 4.01–3.92 (m, 3H, CHCH_2_, CH_2_Pht), 3.77–3.67 (m, 2H, COCH_2_NH), 3.58 (s, 3H, OCH_3_), 3.19–3.16 (m, 2H, CH_2_NH), 3.00–2.86 (m, 1H, CH_2_CH), 1.63–1.17 (m, 15H, 3 × CH_2_, C(CH_3_)_3_); ^13^C-NMR (151 MHz, DMSO-*d*_6_) *δ*/ppm: 172.9 (C=O), 172.1 (C=O), 171.8 (C=O), 170.1 (C=O), 169.4 (C=O), 168.2 (C=O), 160.2, 159.7 (C-2), 158.8, 156.7 (C=O, Cbz), 155.3 (C=O, Boc), 154.4 (C=O, C-6), 140.8 (C-4), 137.3 (C-7a), 134.8 (Pht), 131.8 (Pht), 128.7 (Ph), 127.8 (Ph), 123.5 (Pht), 121.0 (Pyrr), 112.2 (CH-Pyrr), 109.3 (C-4a), 98.7 (C-5), 78.1 (C(CH_3_)_3_), 65.9 (OCH_2_Ph), 55.1 (CHCH_2_), 52.5 (OCH_3_), 51.6 (CHCH_2_), 49.7 (CH_2_Pht), 42.0 (COCH_2_NH), 38.9 (CH_2_NH), 37.4 (CH_2_Pht), 31.6 (CH_2_CH), 29.9 (CH_2_), 29.0 (CH_2_), 28.1 (C(CH_3_)_3_), 23.2 (CH_2_). ESI-MS: *m*/*z* calcd. for C_48_H_53_N_12_O_13_ [M − H]^−^ 1005.39, found 1004.9. 

*Lys(Boc)-Gly-OMe* **16**: Z-d-Lys(Boc)-Gly-OMe **9** (430 mg, 0.95 mmol) was dissolved in methanol (50 mL) and 10% Pd/C (43 mg) was added. The reaction mixture was stirred under a hydrogen atmosphere for 24 h at room temperature, after which time the catalyst was filtered off through a Celite pad and washed several times with methanol. The solvent was evaporated under reduced pressure to provide the product **16** (302 mg, 100%) as a colourless oil: ^1^H-NMR (300 MHz, DMSO-*d*_6_) *δ*/ppm: 8.50–8.46 (m, 1H, NH), 7.64–7.56 (m, 1H, NH), 6.79–6.73 (m, 2H, NH_2_), 4.09 (br. s, 1H, CHCH_2_), 3.96–3.82 (m, 2H, COCH_2_NH), 3.63 (s, 3H, OCH_3_), 2.96–2.82 (m, CH_2_NH), 1.69–1.56 (m, 2H, CH_2_CH), 1.42–1.33 (m, 13H, 2 × CH_2_, C(CH_3_)_3_); ^13^C-NMR (75 MHz, DMSO-*d*_6_) *δ*/ppm: 170.2 (C=O), 168.0(C=O), 166.1 (C=O), 155.6 (C=O), 77.4 (C(CH_3_)_3_), 54.1 (CHCH_2_), 51.7 (OCH_3_), 44.3 (COCH_2_NH), 40.5 (CH_2_NH), 33.3 (CH_2_CH), 29.3 (CH_2_), 28.3 (C(CH_3_)_3_), 22.0 (CH_2_). ESI-MS: *m*/*z* calcd. for C_14_H_28_N_3_O_5_ [M + H]^+^ 318.2, found 318.3. 

*GCP Dipeptide* **17**: Dipeptide **16** (61 mg, 0.192 mmol) and Boc-GCP-OH **13** (76.2 mg, 0.192 mmol) were dissolved in dry CH_3_CN (3 mL) under argon and HOBt (26 mg, 0.192 mmol), HBTU (73 mg, 0.192 mmol) and dry Et_3_N (107 µL, 0.768 mmol) were added. Reaction was stirred at room temperature overnight. Product **17** (67 mg, 58%) was isolated by preparative chromatography (CH_2_Cl_2_/CH_3_OH 9:1) as a yellow powder: ^1^H-NMR (300 MHz, DMSO-*d*_6_) *δ*/ppm: 11.62 (br. s, 1H, NH), 10.88 (br. s, 1H, NH), 9.33 (s, 1H, NH), 8.57 (s, 1H, NH), 8.46–8.41 (m, 2H, 2 × NH), 6.83–6.80 (m, 2H, CH-Pyrr), 6.75 (t, *J* = 5.0 Hz, 1H, NH), 4.47–4.40 (m, 1H, CHCH_2_), 3.85–3.80 (m, 2H, COCH_2_NH), 3.62 (s, 3H, OCH_3_), 2.90–2.88 (m, 2H, CH_2_NH), 1.75–1.64 (m, 2H, CH_2_CH), 1.61–1.23 (m, 22H, 2 × CH_2_, 2 × C(CH_3_)_3_); ^13^C-NMR (75 MHz, DMSO-*d*_6_) *δ*/ppm: 172.4 (C=O), 170.2 (C=O), 159.4, 158.4, 155.5 (C=O, Boc), 113.6 (CH-Pyrr), 112 (CH-Pyrr), 77.3 (C(CH_3_)_3_), 52.5 (CHCH_2_), 51.6 (OCH_3_), 40.5 (COCH_2_NH), 39.8 (CH_2_NH), 31.6 (CH_2_CH), 29.2 (CH_2_), 28.2 (C(CH_3_)_3_), 27.8 (C(CH_3_)_3_), 22.9 (CH_2_). ESI-MS: *m*/*z* calcd. for C_26_H_42_N_7_O_9_ [M + H]^+^ 596.3, found 596.3. 

*GCP Dipeptide* **18**: To a solution of dipeptide **17** (90 mg, 0.147 mmol) in a 4:1 mixture of THF/water (25 mL), lithium hydroxide (37.8 mg, 0.882 mmol) was added and reaction mixture was stirred at room temperature overnight. The solvent was evaporated and a concentrated citric acid solution was added to the water phase (pH ~ 5). White precipitate which formed was filtered and washed several times with water yielding product **18** (64 mg, 75%) as a white soil: ^1^H-NMR (300 MHz, DMSO-*d*_6_) *δ*/ppm: 11.64 (br. s, 3H, 2 × NH, COOH), 9.32 (s, 1H, NH), 8.57 (s, 1H, NH), 8.45 (d, *J* = 8.1 Hz, 1H, NH), 8.24 (t, *J* = 5.7 Hz, 1H, NH), 6.83 (s, 2H, CH-Pyrr), 6.74 (t, *J* = 5.5 Hz, 1H, NH), 4.45–4.41 (m, 1H, CHCH_2_), 3.80–3.66 (m, 2H, COCH_2_NH), 2.89–2.87 (m, 2H, CH_2_NH), 1.83–1.63 (m, 2H, CH_2_CH), 1.60–1.24 (m, 22H, 2 × CH_2_, 2 × C(CH_3_)_3_); ^13^C-NMR (75 MHz, DMSO-*d*_6_) *δ*/ppm: 172.1 (C=O), 171.1 (C=O), 159.4, 158.4, 155.5 (C=O, Boc), 129.4 (Pyrr), 113.7 (CH-Pyrr), 112.9 (CH-Pyrr), 77.3 (C(CH_3_)_3_), 52.5 (CHCH_2_) 40.4 (COCH_2_NH), 39.8 (CH_2_NH), 31.6 (CH_2_CH), 29.2 (CH_2_), 28.2 (C(CH_3_)_3_), 27.8 (C(CH_3_)_3_), 22.9 (CH_2_). ESI-MS: *m*/*z* calcd. for C_25_H_40_N_7_O_9_ [M + H]^+^ 582.29, found 582.4.

*Pyrr-C Tripeptide GCP conjugate* **19**: Compound **1** (27 mg, 0.052 mmol) and dipeptide **18** (30 mg, 0.052 mmol) were dissolved in dry CH_3_CN (5 mL) under argon and HOBt (7 mg, 0.052 mmol), HBTU (19.6 mg, 0.052 mmol) and dry Et_3_N (29 µL, 0.208 mmol) were added. Reaction was stirred at room temperature overnight. Product **19** (25 mg, 50%) was isolated by preparative chromatography (CH_2_Cl_2_/CH_3_OH 9:1) as a yellow powder: ^1^H-NMR (300 MHz, DMSO-*d*_6_) *δ*/ppm: 11.01 (br. s, 2H, NH, NH-7), 9.29 (br. s, 2H, 2 × NH), 8.61–8.23 (m, 5H, 4 × NH, H-4), 7.81 (s, 4H, Pht), 6.80–6.73 (m, 3H, NH, 2 × CH-Pyrr), 5.87 (d, *J* = 12.2 Hz, 1H, H-5), 4.64–4.57 (m, 1H, CHCH_2_), 4.33 (br. s, 1H, CHCH_2_), 4.20–4.07 (m, 2H, CH_2_Pht), 3.97–3.88 (m, 2H, CH_2_Pht), 3.74–3.65 (m, 2H, COCH_2_NH), 3.57 (m, 3H, OCH_3_), 3.03–2.87 (m, 4H, CH_2_CH, CH_2_NH), 1.80–1.56 (m, 2H, CH_2_CH), 1.49–1.23 (m, 22H, 2 × CH_2_, 2 × C(CH_3_)_3_); ^13^C-NMR (75 MHz, DMSO-*d*_6_) *δ*/ppm: 172.1 (C=O), 171.4 (C=O), 169.9 (C=O), 168.9 (C=O), 167.7 (C=O), 167.0, 159.5 (C-2), 158.5, 155.5 (C=O, Boc), 154.7 (C-6), 140.3 (C-4), 136.8 (C-7a), 134.3 (Pht), 131.6 (Pht), 123.1 (Pht), 113.1 (CH-Pyrr), 111.9 (CH-Pyrr), 108.6 (C-4a), 98.1 (C-5), 77.3 (C(CH_3_)_3_), 68.3 (OCH_2_Ph), 53.1 (CHCH_2_), 52.0 (OCH_3_), 51.1 (CHCH_2_), 49.3 (CH_2_Pht), 41.6 (COCH_2_NH), 39.7 (CH_2_NH), 37.0 (CH_2_Pht), 31.3 (CH_2_CH), 29.6 (CH_2_), 29.2 (CH_2_), 28.2 (C(CH_3_)_3_), 27.8 (C(CH_3_)_3_), 22.9 (CH_2_). ESI-MS: *m*/*z* calcd. for C_45_H_55_N_12_O_13_ [M − H]^−^ 971.4, found 971.0.

*Conjugate Pyrr-C Tripeptide* **2**: Compound **19** (14 mg, 0.014 mmol) was dissolved in 1:1 mixture of TFA/CH_2_Cl_2_ (4 mL) and stirred at room temperature for 20 h. After removal of remaining TFA under reduced pressure, product **2** (15 mg, 100%) was obtained as a yellow foam: ^1^H-NMR (300 MHz, DMSO-*d*_6_) *δ*/ppm: 12.57–12.28 (m, 1H, NH), 11.37 (br. s, 1H, NH), 10.98–10.92 (m, 1H, NH-7), 8.59–8.26 (m, 8H, 4 × NH, NH_3_, H-4), 7.81–7.70 (m, 7H, Pht, NH_3_), 7.12 (s, 1H, CH-Pyrr), 6.89 (s, 1H, CH-Pyrr), 5.91–5.88 (m, 1H, H-5), 4.63–4.61 (m, 1H, CHCH_2_), 4.43 (s, 1H, CHCH_2_), 4.18–4.13 (m, 2H, CH_2_Pht), 3.94 (m, 2H, CH_2_Pht), 3.76–3.67 (m, 2H, COCH_2_NH), 3.59 (s, 3H, OCH_3_), 3.01–2.77 (m, 4H, CH_2_CH, CH_2_), 1.76–1.23 (m, 6H, 3 × CH_2_); ^13^C-NMR (151 MHz, DMSO-*d*_6_) *δ*/ppm: 171.8 (C=O), 171.4 (C=O), 168.8 (C=O), 167.7 (C=O), 159.4 (C-2), 159.1, 158.4, 158.2, 158.0, 157.8 (4 × CF_3_C=O), 154.8 (C-6), 140.4 (C-4), 136.9 (C-7a), 134.3 (Pht), 131.6 (Pht), 123.1 (Pht), 118.2 (CF_3_C=O), 114.9 (CH-Pyrr), 113.6 (CH-Pyrr), 108.7 (C-4a), 98.3 (C-5), 68.3 (OCH_2_Ph), 52.7 (CHCH_2_), 52.0 (CHCH_2_), 51.3 (OCH_3_), 49.3 (CH_2_Pht), 41.5 (COCH_2_NH), 38.7 (CH_2_NH), 37.0 (CH_2_Pht), 31.2 (CH_2_CH), 29.6 (CH_2_), 26.6 (CH_2_), 22.4 (CH_2_). HRMS (MALDI-TOF/TOF): *m*/*z* calcd. for C_35_H_40_N_12_O_9_ [M + H]^+^ 773.3119; found 773.3137. 

*Boc-d-Lys(Z)-Gly-OMe* **20**: Boc-d-Lyz(Z)-OH (400 mg, 1.051 mmol) and H-Gly-OMe hydrochloride (131.5 mg, 1.051 mmol) were dissolved in dry CH_3_CN (10 mL) under argon and HOBt (141.9 mg, 1.051 mmol), HBTU (398.2 mg, 1.051 mmol) and dry Et_3_N (585 µL, 4.2 mmol) were added. Reaction was stirred at room temperature overnight. Product **20** (455 mg, 96%) was isolated by preparative chromatography (CH_2_Cl_2_/CH_3_OH 9:1) as a colorless oil: ^1^H-NMR (300 MHz, DMSO-*d*_6_) *δ*/ppm: 8.21 (t, *J* = 5.8 Hz, 1H, NH), 7.39–7.32 (m, 5H, Ph), 7.22 (t, *J* = 5.3 Hz, 1H, NH), 6.83 (d, *J* = 8.1 Hz, 1H, NH), 5.00 (s, 2H, OCH_2_Ph), 3.92–3.74 (m, 3H, CHCH_2_, COCH_2_NH), 3.61 (s, 3H, OCH_3_), 3.00–2.96 (m, 2H, CH_2_NH), 1.47–1.24 (m, 15H, 3 × CH_2_, C(CH_3_)_3_); ^13^C-NMR (75 MHz, DMSO-*d*_6_) *δ*/ppm: 172.8 (C=O), 170.3 (C=O), 156.1 (C=O, Cbz), 155.3 (C=O, Boc), 137.3 (Ph), 128.4 (Ph), 127.7 (Ph), 78.0 (C(CH_3_)_3_), 65.1 (OCH_2_Ph), 54.1 (CHCH_2_), 51.6 (OCH_3_), 40.5 (COCH_2_NH), 40.1 (CH_2_NH), 31.6 (CH_2_CH), 29.1 (CH_2_), 28.2 (C(CH_3_)_3_), 22.7 (CH_2_). ESI-MS: *m*/*z* calcd. for C_22_H_33_N_3_NaO_7_ [M + Na]^+^ 474.22, found 474.22.

*Boc-d-Lys-Gly-OMe* **21**: Cbz-protected compound **20** (380 mg, 0.842 mmol) was dissolved in methanol (50 mL) and 10% Pd/C (38 mg) was added. The reaction mixture was stirred under the hydrogen for 16 h at room temperature, after which time the catalyst was filtered off through a Celite pad and washed several times with methanol. The solvent was evaporated under reduced pressure to provide the product **21** (267 mg, 100%) as a colourless oil: ^1^H-NMR (300 MHz, DMSO-*d*_6_) *δ*/ppm: 8.29 (t, *J* = 5.8 Hz, 1H, NH), 6.89 (d, *J* = 8.1 Hz, 1H, NH), 4.50 (br. s, 2H, NH_2_), 3.96–3.74 (m, 3H, CHCH_2_, COCH_2_NH), 3.62 (s, 3H, OCH_3_), 2.60 (t, *J* = 6.9 Hz, 2H, CH_2_NH_2_), 1.66–1.16 (m, 15H, 3 × CH_2_, C(CH_3_)_3_); ^13^C-NMR (75 MHz, DMSO-*d*_6_) *δ*/ppm: 172.5 (C=O), 167.0 (C=O), 155.0 (C=O, Boc), 77.7 (C(CH_3_)_3_), 53.7 (CHCH_2_), 51.4 (OCH_3_), 40.5 (COCH_2_NH), 39.8 (CH_2_NH), 31.2 (CH_2_CH), 29.3 (CH_2_), 27.9 (C(CH_3_)_3_), 22.2 (CH_2_). ESI-MS: *m*/*z* calcd. for C_14_H_28_N_3_O_5_ [M + H]^+^ 318.2, found 318.2.

*GCP Dipeptide* **22**: Compound **21** (103 mg, 0.325 mmol) and Boc-GCP-OH **13** (128.9 mg, 0.325 mmol) were dissolved in dry CH_3_CN (8 mL) under argon and HOBt (43.9 mg, 0.325 mmol), HBTU (123.2 mg, 0.325 mmol) and dry Et_3_N (181 µL, 1.3 mmol) were added. Reaction was stirred at room temperature overnight. Product **22** (78 mg, 40%) was isolated by preparative chromatography (eluent: CH_2_Cl_2_/CH_3_OH 9:1) as a slightly yellow oil: ^1^H-NMR (300 MHz, DMSO-*d*_6_) *δ*/ppm: 11.30 (br. s, 2H, 2 × NH), 9.27 (s, 1H, NH), 8.53–8.22 (m, 3H, 3 × NH), 6.86 (d, *J* = 8.2 Hz, 1H, NH), 6.75 (d, *J* = 3.6 Hz, 1H, CH-Pyrr), 6.66 (d, *J* = 3.6 Hz, 1H, CH-Pyrr), 3.96–3.74 (m, 3H, CHCH_2_, COCH_2_NH), 3.61 (s, 3H, OCH_3_), 3.22–3.16 (m, 2H, CH_2_NH), 1.50–1.29 (m, 24H, 3 × CH_2_, 2 × C(CH_3_)_3_); ^13^C-NMR (75 MHz, DMSO-*d*_6_) *δ*/ppm: 172.8 (C=O), 170.2 (C=O), 158.5, 155.2 (C=O, Boc), 113.6 (CH-Pyrr), 111.9 (CH-Pyrr), 77.9 (C(CH_3_)_3_), 54.0 (CHCH_2_), 51.6 (OCH_3_), 40.5 (COCH_2_NH), 38.3 (CH_2_NH), 31.6 (CH_2_CH), 29.0 (CH_2_), 28.2 (C(CH_3_)_3_), 27.8 (C(CH_3_)_3_), 22.9 (CH_2_). ESI-MS: *m*/*z* calcd. for C_26_H_42_N_7_O_9_ [M + H]^+^ 596.3, found 596.3.

*GCP Dipeptide* **23**: To a solution of **22** (69 mg, 0.116 mmol) in a 4:1 mixture of THF/water (25 mL), lithium hydroxide (29.2 mg, 0.696 mmol) was added and reaction mixture was stirred at room temperature overnight. The solvent was evaporated and a concentrated citric acid solution was added to the water phase (pH ~ 5). The product was extracted with EtOAc (5 × 20 mL), dried over Na_2_SO_4_ yielding **23** (47 mg, 70%) as a white foam: ^1^H-NMR (300 MHz, DMSO-*d*_6_) *δ*/ppm: 12.07–11.84 (m, 2H, NH, COOH), 11.32 (s, 1H, NH), 9.32 (s, 1H, NH), 8.57 (s, 1H, NH), 8.30 (t, *J* = 5.3 Hz, 1H, NH), 8.06 (t, *J* = 5.4 Hz, 1H, NH), 6.84–6.72 (m, 3H, NH, 2 × CH-Pyrr), 3.97–3.89 (m, 1H, CHCH_2_), 3.84–3.65 (m, 2H, COCH_2_NH), 3.24–3.17 (m, 2H, CH_2_NH), 1.68–1.23 (m, 24H, 3 × CH_2_, 2 × C(CH_3_)_3_); ^13^C-NMR (75 MHz, DMSO-*d*_6_) *δ*/ppm: 172.5 (C=O), 171.1 (C=O), 159.4, 159.2, 158.4, 155.3 (C=O, Boc), 129.7, 125.2, 112.3 (CH-Pyrr), 111.6 (CH-Pyrr), 77.9 (C(CH_3_)_3_), 54.3 (CHCH_2_), 40.6 (COCH_2_NH), 38.5 (CH_2_NH), 31.7 (CH_2_CH), 28.8 (CH_2_), 28.2 (C(CH_3_)_3_), 27.8 (C(CH_3_)_3_), 22.9 (CH_2_). ESI-MS: *m*/*z* calcd. for C_25_H_38_N_7_O_9_ [M − H]^−^ 580.27, found 580.1.

*Pyrr-C Tripeptide GCP conjugate* **24**: Compound **1** (32 mg, 0.061 mmol) and GCP-dipeptide **23** (35.55 mg, 0.061 mmol) were dissolved in dry CH_3_CN (7 mL) under argon and HOBt (8.27 mg, 0.061 mmol), HBTU (23.20 mg, 0.061 mmol) and dry Et_3_N (34 µL, 0.245 mmol) were added. Reaction was stirred at room temperature overnight. Product **24** (28 mg, 47%) was isolated by preparative chromatography (CH_2_Cl_2_/CH_3_OH 9:1) as a white powder: ^1^H-NMR (300 MHz, DMSO-*d*_6_) *δ*/ppm: 11.03 (br. s, 2H, NH, NH-7), 9.59 (br. s, 1H, NH), 9.29 (br. S, 1H, NH), 8.58–8.50 (m, 2H, 2 × NH), 8.34–8.26 (m, 2H, NH, H-4), 8.04 (br. s, 1H, NH), 7.81 (s, 4H, Pht), 6.95–6.73 (m, 3H, CH-Pyrr, 2 × NH), 6.45 (d, *J* = 3.7 Hz, 1H, CH-Pyrr), 5.87 (s, 1H, H-5), 4.64–4.57 (m, 1H, CHCH_2_), 4.20–4.08 (m, 3H, CH_2_Pht, CHCH_2_), 3.95–3.89 (m, 2H, CH_2_Pht), 3.71–3.67 (m, 2H, COCH_2_NH), 3.58 (s, 3H, OCH_3_), 3.03–2.69 (m, 4H, CH_2_CH, CH_2_NH), 1.66–1.23 (m, 24H, 3 × CH_2_, 2 × C(CH_3_)_3_); ^13^C-NMR (75 MHz, DMSO-*d*_6_) *δ*/ppm: 172.4 (C=O), 171.4 (C=O), 168.9 (C=O), 168.8, 167.7 (C=O), 159.5, 158.9 (C-2), 158.6, 158.5, 155.4 (C=O, Boc), 154.8 (C-6), 140.4 (C-4), 136.8 (C-7a), 134.3 (Pht), 131.6 (Pht), 123.1 (Pht), 113.6 (CH-Pyrr), 111.7 (CH-Pyrr), 108.6 (C-4a), 98.2 (C-5), 78.1 (C(CH_3_)_3_), 63.8 (CH_2_), 54.3 (CHCH_2_), 52.0 (OCH_3_), 51.2 (COCH_2_NH), 49.3 (CH_2_Pht), 41.6 (COCH_2_NH), 40.6 (CH_2_NH), 37.0 (CH_2_Pht), 31.5 (CH_2_CH), 29.6 (CH_2_), 29.0 (CH_2_), 28.2 (C(CH_3_)_3_), 27.7 (C(CH_3_)_3_), 22.8 (CH_2_). ESI-MS: *m*/*z* calcd. for C_45_H_57_N_12_O_13_ [M + H]^+^ 973.42, found 973.4. 

*Pyrr-C Tripeptide GCP conjugate* **3**: Compound **24** (19 mg, 0.02 mmol) was dissolved in 1:1 mixture of TFA/CH_2_Cl_2_ (4 mL) and stirred at room temperature for 20 h. After removal of remaining TFA under reduced pressure, the residue was purified by preparative chromatography (CH_2_Cl_2_/MeOH 9:1) and product **3** (19 mg, 95%) was obtained as a yellow foam; ^1^H-NMR (300 MHz, DMSO-*d*_6_) *δ*/ppm: 12.20 (br. s, 1H, NH), 11.18–10.97 (m, 2H, NH-7, NH), 8.66 (br. s, 1H, NH), 8.55–8.11 (m, 9H, 2 × NH, 2 × NH_3_, H-4), 7.81 (s, 4H, Pht), 7.54 (s, 1H, NH), 7.02 (br. s, 1H, CH-Pyrr), 6.53 (s, 1H, CH-Pyrr), 5.88 (s, 1H, H-5), 4.63 (br. s, 1H, CHCH_2_), 4.17–4.14 (m, 3H, CH_2_Pht, CHCH_2_), 3.95–3.91 (m, 2H, CH_2_Pht), 3.81–3.79 (m, 2H, COCH_2_NH), 3.59 (m, 3H, OCH_3_), 2.95–2.82 (m, 4H, CH_2_CH, CH_2_NH), 1.73–1.24 (m, 6H, 3 × CH_2_); ^13^C-NMR (75 MHz, DMSO-*d*_6_) *δ*/ppm: 169.7 (C=O), 168.9, 168.2, 167.7, 158.1, 157.6, 154.7 (C-6), 140.4 (C-4), 136.8 (C-7a), 134.3 (Pht), 131.6 (Pht), 123.0 (Pht), 113.8 (CH-Pyrr), 113.0 (CH-Pyrr), 108.6 (C-4a), 98.2 (C-5), 62.6 (CH_2_), 52.3 (CHCH_2_), 52.1 (OCH_3_), 51.2 (COCH_2_NH), 49.3 (CH_2_Pht), 41.5 (COCH_2_NH), 38.4 (CH_2_NH), 37.0 (CH_2_Pht), 30.8 (CH_2_CH), 28.9 (CH_2_), 27.2 (CH_2_), 21.4 (CH_2_). HRMS (MALDI-TOF/TOF): *m*/*z* calcd. for C_35_H_40_N_12_O_9_ [M + H]^+^ 773.3114; found 773.3102.

### 3.3. Computational Details

In order to sample the conformational flexibility of investigated systems and probe their intrinsic dynamical features in the aqueous solution, classical molecular dynamics (MD) simulations were performed employing standard generalized AMBER force fields (ff14SB and GAFF) [51] as implemented within the AMBER16 program package [52]. All structures were subsequently solvated in a truncated octahedral box of TIP3P water molecules spanning a 10 Å thick buffer of solvent molecules around each system. Upon gradual heating from 0 K, MD simulations were performed at 400 K for a period of 100 ns, maintaining the temperature constant using the Langevin thermostat with a collision frequency of 1 ps^−1^. Following the clustering analysis of the structures in the obtained MD trajectories, around 20–25 different structures in each case were subjected to geometry re-optimization in Gaussian09 [53] at the M06–2X/6–31G(d) level of theory with aqueous solution being modelled through the implicit SMD solvation. The idea behind this computational strategy was to investigate whether intrinsic dynamical features of investigated conjugates both affect and can explain their tendency to interact with ss-polynucleotides, which avoids difficulties and inaccuracies associated with the computational prediction of the structure of single-stranded polynucleotides, as very recently emphasized by Jeddi and Saiz [54]. p*K*_a_ values were calculated in a relative fashion with the MP2/6–311++G(2df,2pd)//(SMD)/M06–2X/6–31+G(d) model, using AH^+^ + B_REF_ → A + B_REF_H^+^ equation, and employing the following reference bases (B_REF_): succinimide (p*K*_a_ = 3.9) [55] for the *O*-protonation in **I**, cytosine (p*K*_a_ = 4.5) [55] for the N-protonation in **II**, and guanidine (p*K*_a_ = 13.6) [55] for the *N*-protonation in **III**. The choice of such computational setup was prompted by its recent success in evaluating solution-phase geometries, reactivities and p*K*_a_ values of a large variety of systems [56,57,58,59].

## 4. Conclusions

Two structural isomers of fluorescent cytosine derivative conjugated with guanidiniocarbonyl-pyrrole (GCP) were prepared, whereby they differ in the position of GCP: **2** being more rigid due to the proximity of GCP to the peptide backbone, while **3** is more flexible with GCP attached at the end of a long aliphatic chain of lysine.

In comparison with previously studied GCP derivatives [21,22,23,24,25], here studied **2**, **3** did not induce thermal stabilisation of ds-DNA/RNA nor showed ICD bands of GCP about 300 nm, which supported random aggregation of compounds along double stranded helix of polynucleotide, based on non-specific electrostatic interactions of positive charge with DNA/RNA backbone and hydrophobic interactions. However, even such non-defined binding was highly sensitive to ds-polynucleotide secondary structure, at pH 7 giving fluorescence increase only for ds-DNA, but not ds-RNA. 

Intriguingly, **2** and **3** interacted with single stranded (ss) RNA with similar affinity or in case of **3**/poly G order of magnitude higher affinity than with ds-DNA/RNA. With ss-polynucleotides fluorescence emission of nucleobase-phthalimide fluorophore in **2** and **3** became very selective: at pH 7 **2** revealed highly selective emission increase only for poly A, while emission of **3** was selectively quenched only by addition of poly G. Observed selectivities can only be attributed to the positioning of the GCP unit, whereby more flexible combination in **3** prefers poly G as a Watson-Crick partner for cytosine, while more rigid **2** responds only to the mismatched poly A. Molecular modelling confirmed the important role of the GCP fragment in sensing of ss-polynucleotides in solution and showed that these interactions can be rationalized by intrinsic dynamical features of **2** and **3** themselves. Additionally, the evidence is provided that successive recognition of ss-polynucleotides is influenced by a fine interplay between two factors: (a) the protonation state of the GCP moiety; and (b) the ability of the GCP unit to form hydrogen bonds with the rest of the molecule. In general, protonated GCP groups are slightly more susceptible towards ss-polynucleotides (as in **3^2+^**) unless these monocationic units are stabilized intramolecularly, which then diminishes their sensing ability (as in **2^2+^**).

At pH 5 the fluorimetric response of the cytosine-phthalimide fluorophore changed significantly. The poly A selectivity of **2** was completely lost, which could be attributed either to the rearrangement of the polynucleotide into the ds-form poly AH^+^-poly AH^+^ or according to a molecular modelling study (Figure 6), to the stronger intramolecular packing of **2**, in which GCP unit is strongly involved. However, same poly AH^+^-poly AH^+^ induced the fluorescence increase of more flexible **3**, which has less intramolecularly blocked GCP unit and therefore was able to adapt to a new binding motif within ds-RNA and direct nucleobase fluorophore in interactions causing strong fluorescence change.

The general conclusion is that due to the fine differences in intramolecular interactions of **2** or **3** and their interactions with ss-RNA at different pH it is possible to finely tune recognition of adenine versus guanine sequences, while cytosine and uracil did not cause measurable fluorimetric changes.

Here presented results demonstrated for the first time that the intriguing GCP unit can be applied for the recognition of single stranded RNA sequences—an intuitively unexpected result since so far binding of GCP unit to ds-DNA or ds-RNA relied strongly on minor or major groove interactions. Since ss-polynucleotides do not have structurally defined grooves, clearly GCP unit can take part in H-bonding and/or electrostatic interactions in a selective manner, thus expanding its uses in the design of novel DNA or RNA targeting small molecules. Thus, nucleobase–GCP conjugates can be considered as novel lead compounds for the design of ss-RNA or ss-DNA selective fluorimetric probes.

We have performed the preliminary screening of in vitro activity of **2** and **3** against the human tumor cell lines “HeLa Kyoto” and “HEK293T” by standard MTT test. Cytotoxicity of compounds was tested in the *c* = 10^−4^–10^−6^ M range and no effect on cell proliferation was observed nor cell morphology was changed. These results indicate negligible cytotoxicity of studied compounds and encourage further studies of compounds as in vitro markers.

## Figures and Tables

**Table 1:** Binding constants (log *K*_s_) ^a^ calculated from the fluorescence titrations (λ_exc_ = 355 nm) of **2** and **3** with ds- and ss-polynucleotides at pH = 7.0 and pH = 5.0 (buffer sodium cacodylate, *I* = 0.05 mol dm^−3^).

Polynucleotide	2		3	
	pH 7	pH 5	pH 7	pH 5
	log *K*_s_/ ^c^ *I*/*I*_0_	log *K*_s_/ ^c^ *I*/*I*_0_	log *K*_s_/ ^c^ *I*/*I*_0_	log *K*_s_/ ^c^ *I*/*I*_0_
ct-DNA	3.7/1.8	4.0/1.5	3.7/1.6	4.4/1.5
poly A–poly U	^b^	3.5/1.8	^b^	^b^
poly A	4.1/2.4	^b^	^b^	^d^ 4/1.2
poly G	^b^	^b^	^d^ 5/0.8	^d^ 4/0.9
poly C	^b^	^b^	^b^	^b^
poly U	^b^	^b^	^b^	^b^

^a^ Processing of titration data by means of Scatchard equation [40,41] gave values of ratio *n*[bound **2**, **3**]/[polynucleotide] = 0.1–0.3, for easier comparison all log *K*_s_ values were re-calculated for fixed *n* = 0.2. Correlation coefficients were >0.99 for all calculated *K*_s_; ^b^ Too small changes for accurate processing by Scatchard eq; ^c^ *I*_0_—starting fluorescence intensity of dye; *I*—fluorescence intensity of dye/polynucleotide complex calculated by Scatchard equation; ^d^ Small changes allowed only the estimation of log *K*_s_.

## Supplementary Materials

Supplementary Materials are available online, Additional characterization data, DNA/RNA binding data.
