# Supplementary material for: Nucleobase–Guanidiniocarbonyl-Pyrrole Conjugates as Novel Fluorimetric Sensors for Single Stranded RNA
Source: Molecules. 2017 Dec 13;22(12):2213. doi: 10.3390/molecules22122213 (PMC6149679; doi:10.3390/molecules22122213)
Supplement: Supplementary file 1 [file molecules-22-02213-s001.pdf]

Supporting information to Ban et al:

Nucleobase - Guanidiniocarbonyl-pyrrole conjugates as novel fluorimetric sensors for single stranded RNA

Željka Ban[a], Biserka Žinić[a], Robert Vianello[a], Carsten Schmuck[b], Ivo Piantanida[a]*

[a] *Division of Organic Chemistry and Biochemistry, Ruđer Bošković Institute, Bijenička c. 54, 10000 Zagreb, Croatia*

[b] *Institute for Organic Chemistry, University of Duisburg-Essen, Universitätsstraße 7, 45141 Essen (Germany)*

Content | Page

^{1}H NMR and ^{13}C NMR spectra of final compounds 1- 3 and intermediates 8, 9, 18, 19, 20, 23, 24 S2-S11

Spectroscopic experiments S12-S33

Structures of the most representative conformations of 2^+, 2^{2+}, 3^+ and 3^{2+} S34-S37

References S37

The compound 3*H*-pyrrolo[2,3-*d*] pyrimidin-2(7*H*)-one (pyrrolocytosine) is abbreviated as Pyrr-C, with numbering of rings shown in **Figure S1**

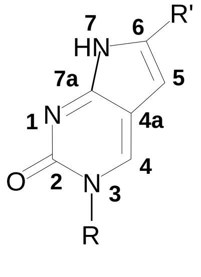

Figure S1

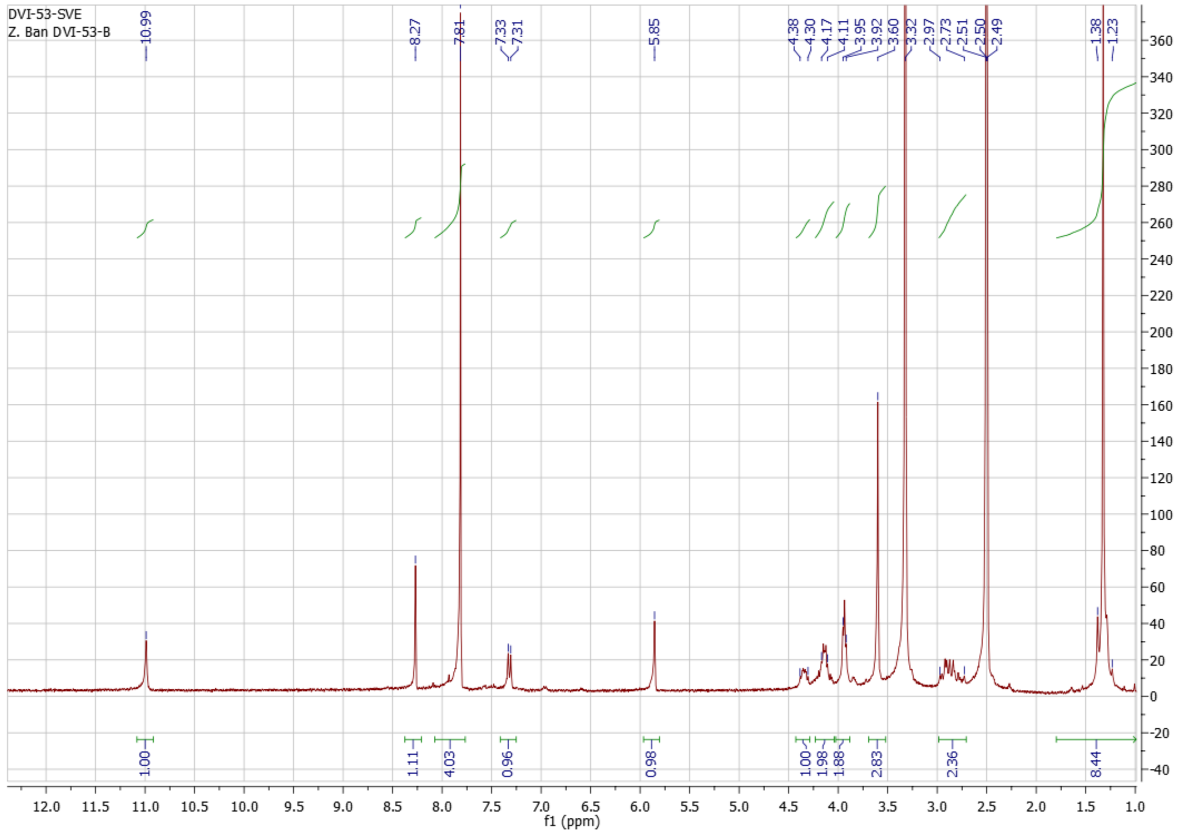

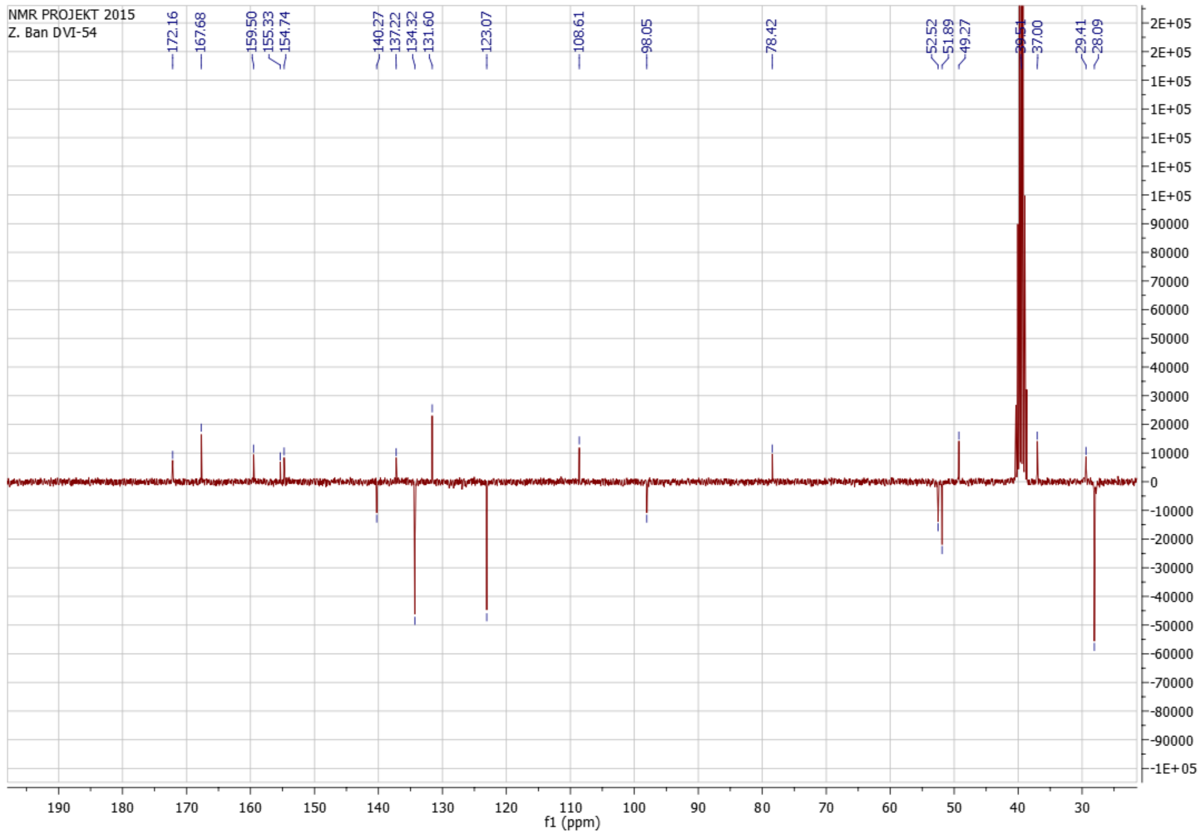

Figure S2 ^{1}H NMR (300 MHz, DMSO-*d*6) and ^{13}C NMR spectra (75 MHz, APT, DMSO-*d*6) of compound **8**.

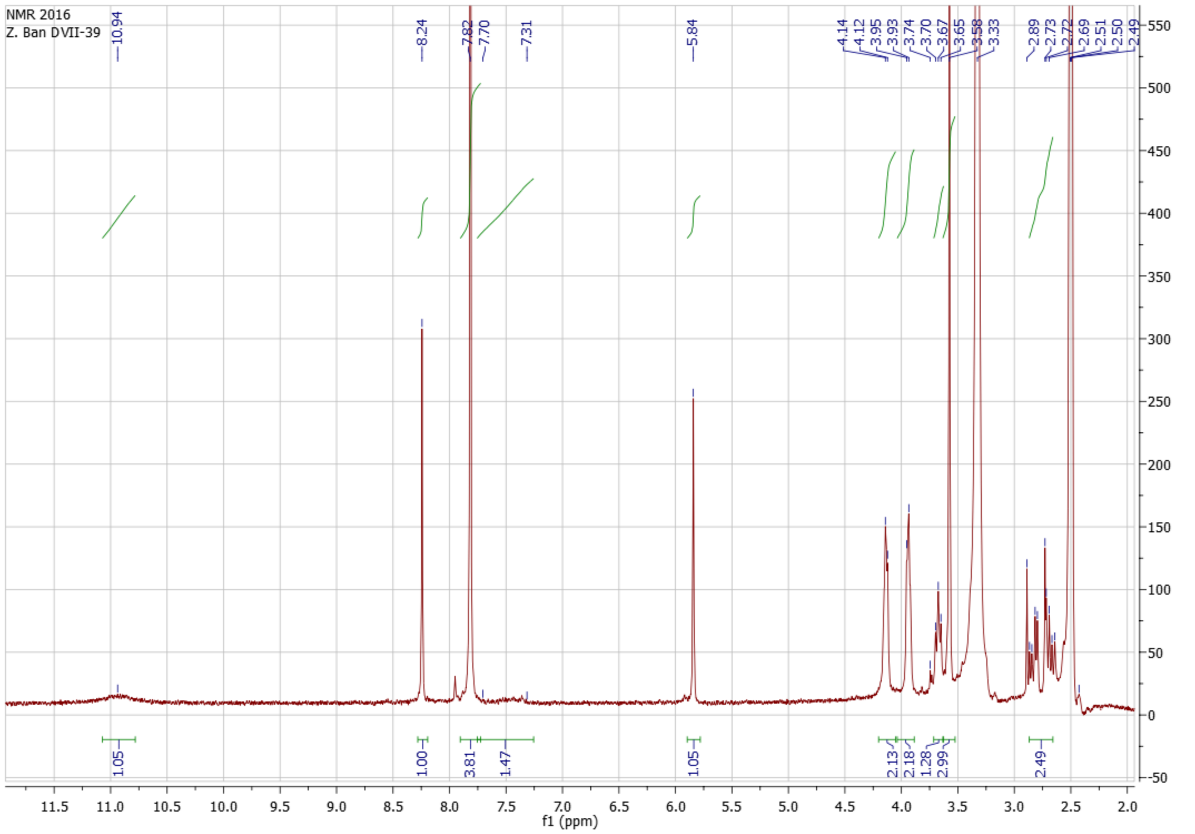

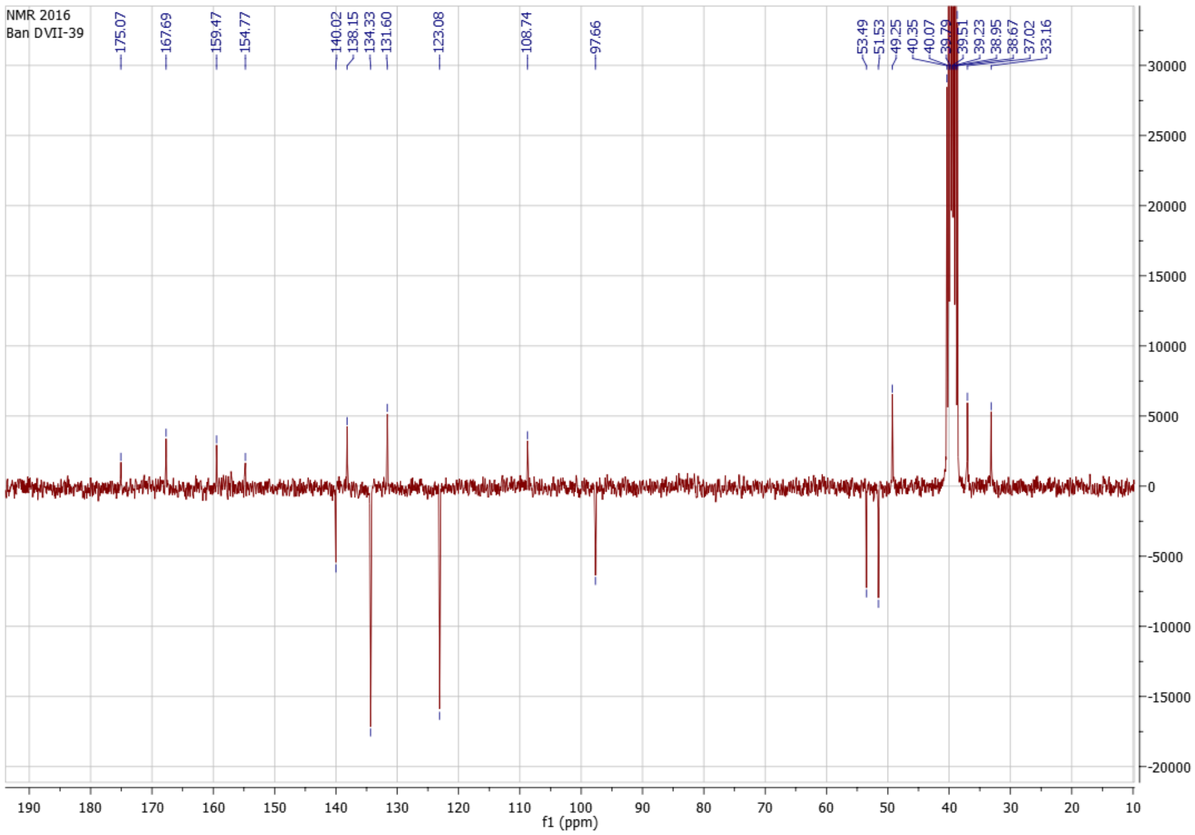

Figure S3 ^{1}H NMR (300 MHz, DMSO-*d*6) and ^{13}C NMR spectra (75 MHz, APT, DMSO-*d*6) of compound **1**.

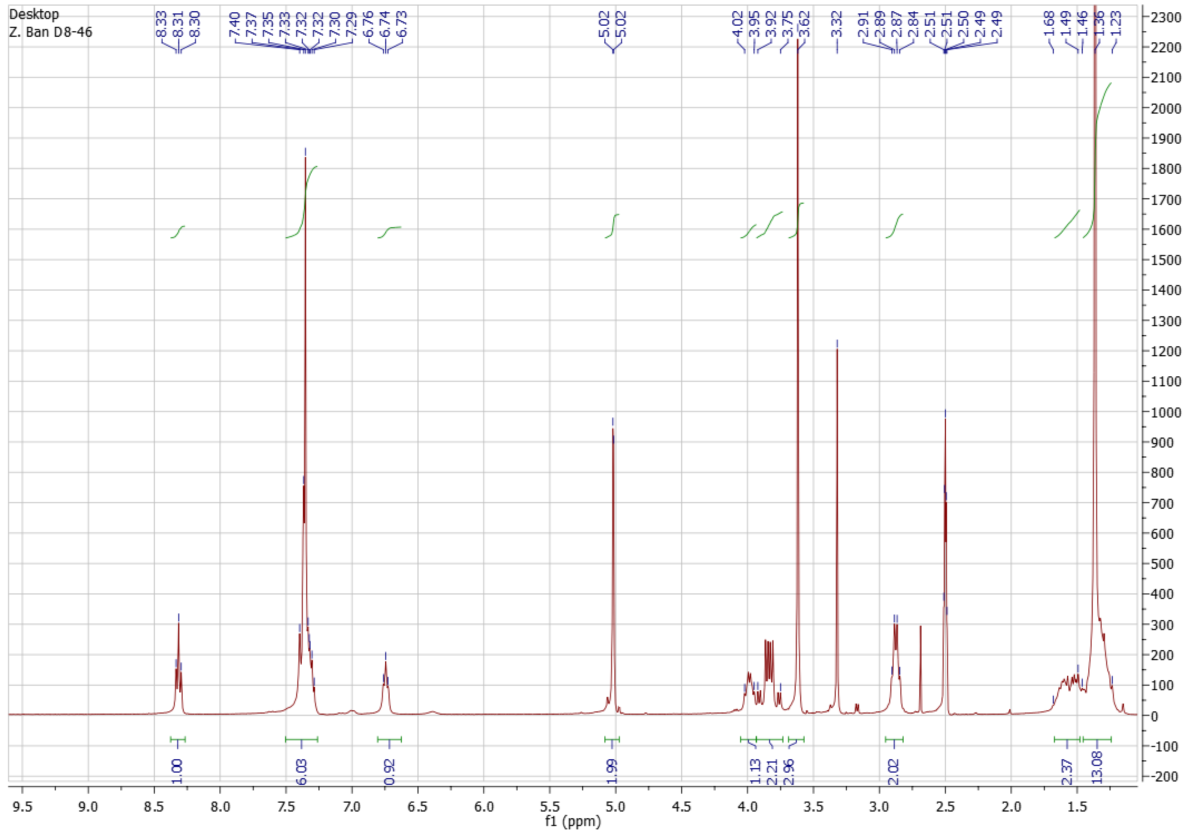

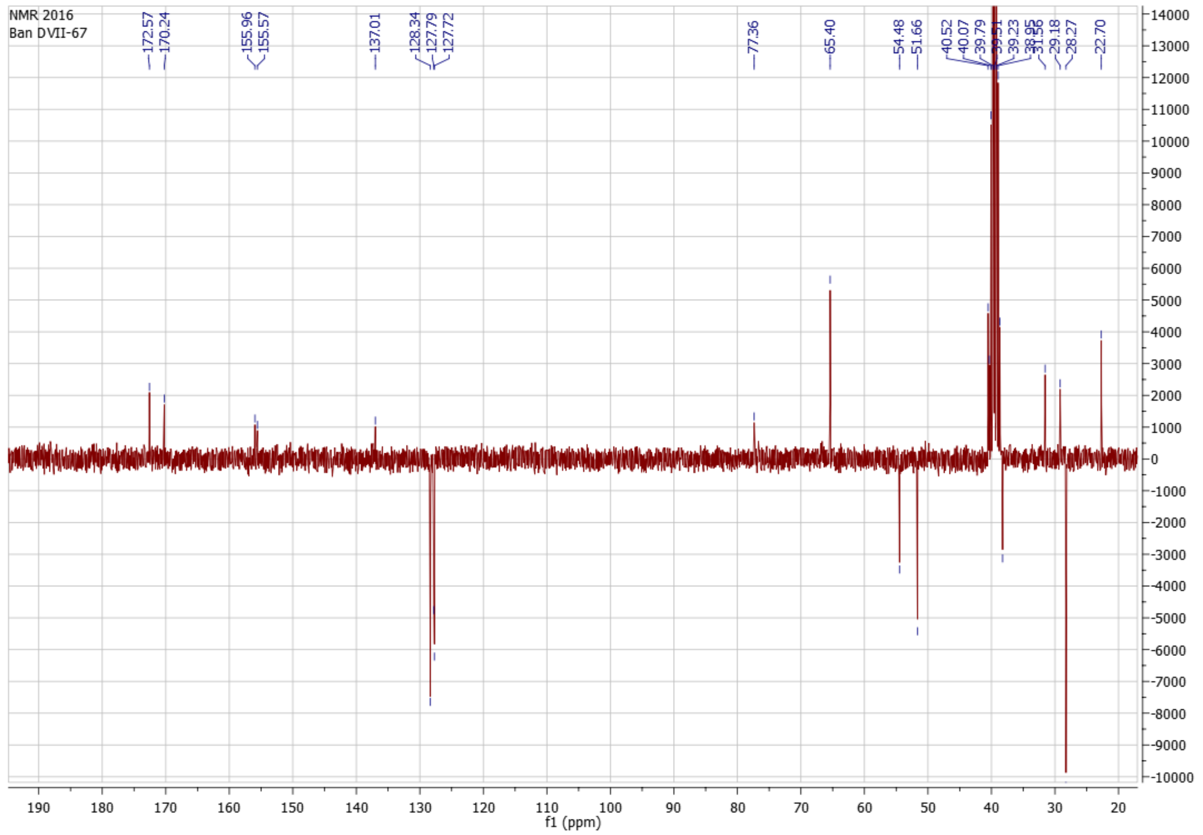

Figure S4 ^{1}H NMR (300 MHz, DMSO-*d*6) and ^{13}C NMR spectra (75 MHz, APT, DMSO-*d*6) of compound **9**.

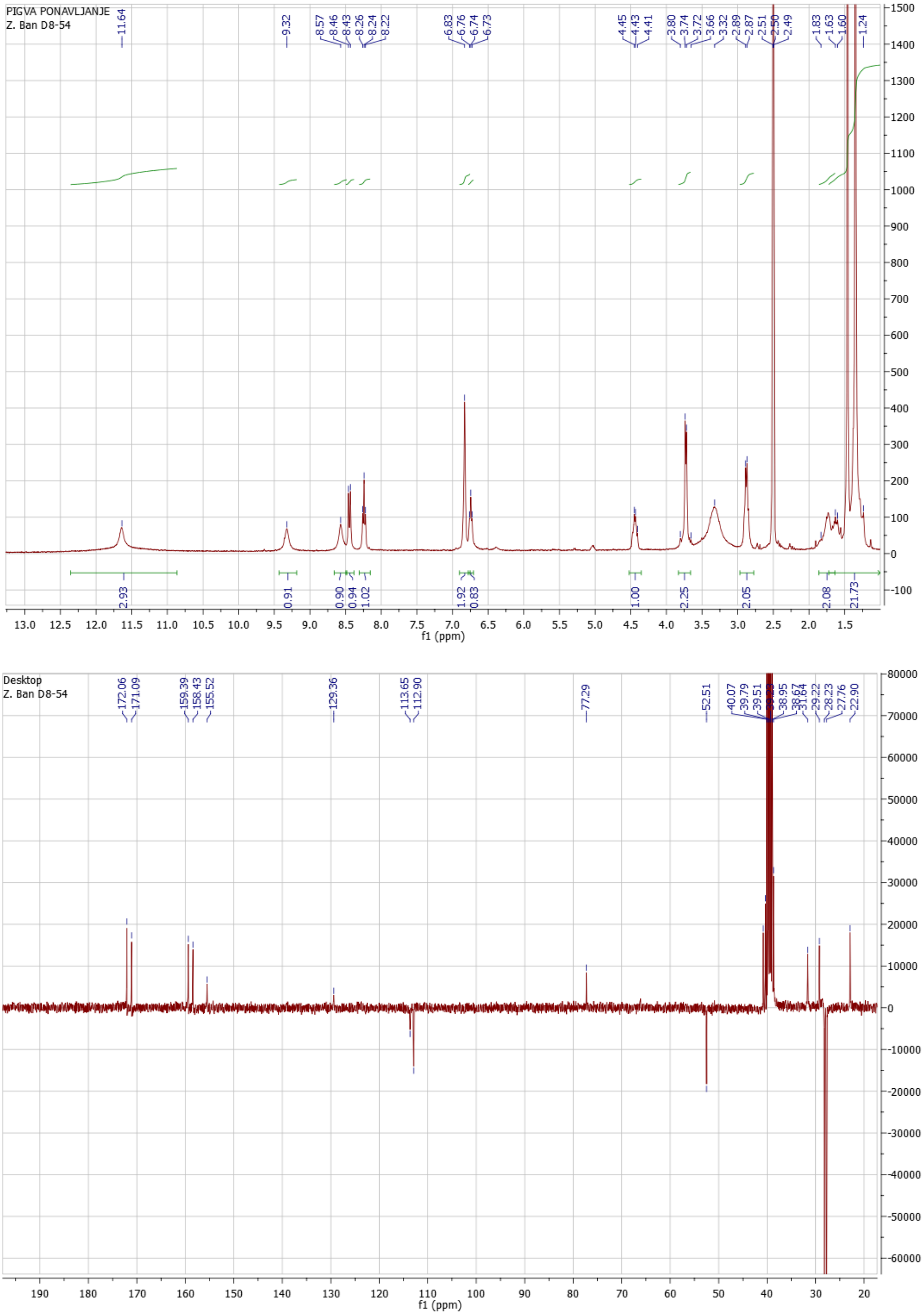

Figure S5 ^{1}H NMR (300 MHz, DMSO-*d*6) and ^{13}C NMR spectra (75 MHz, APT, DMSO-*d*6) of compound **18**.

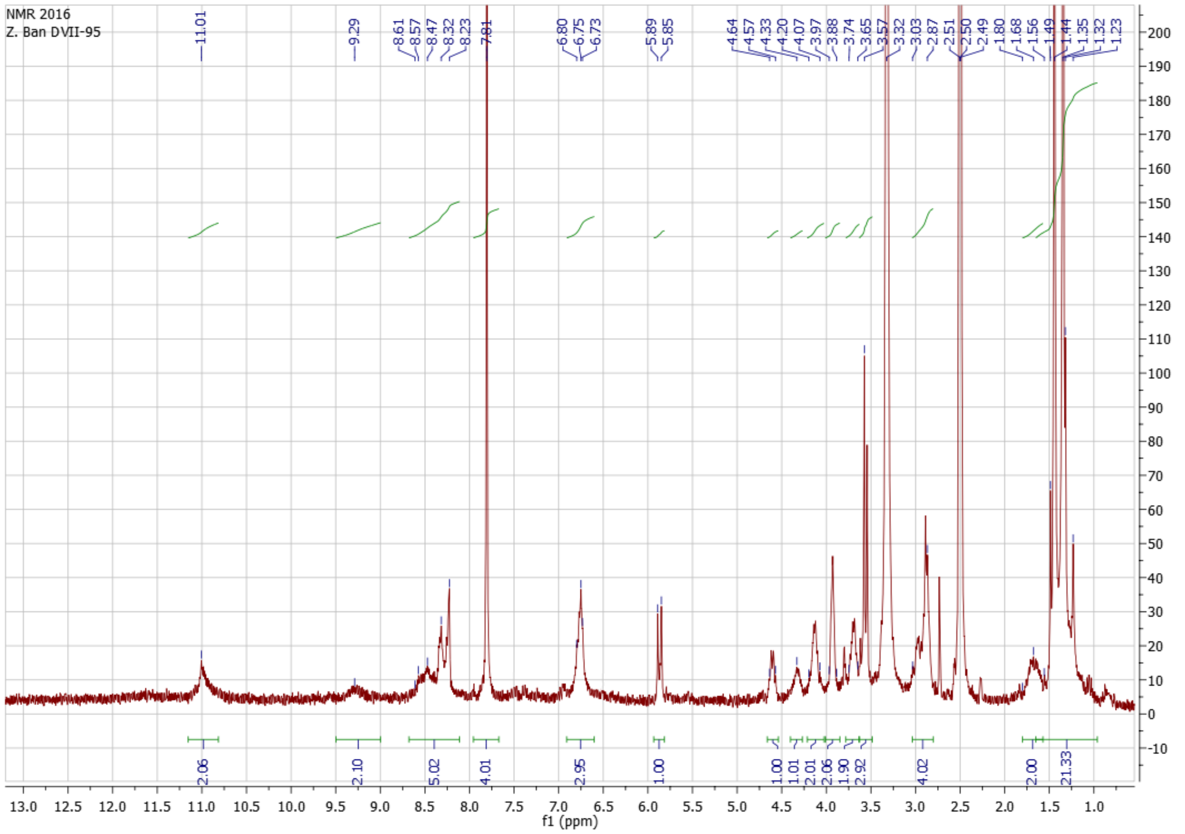

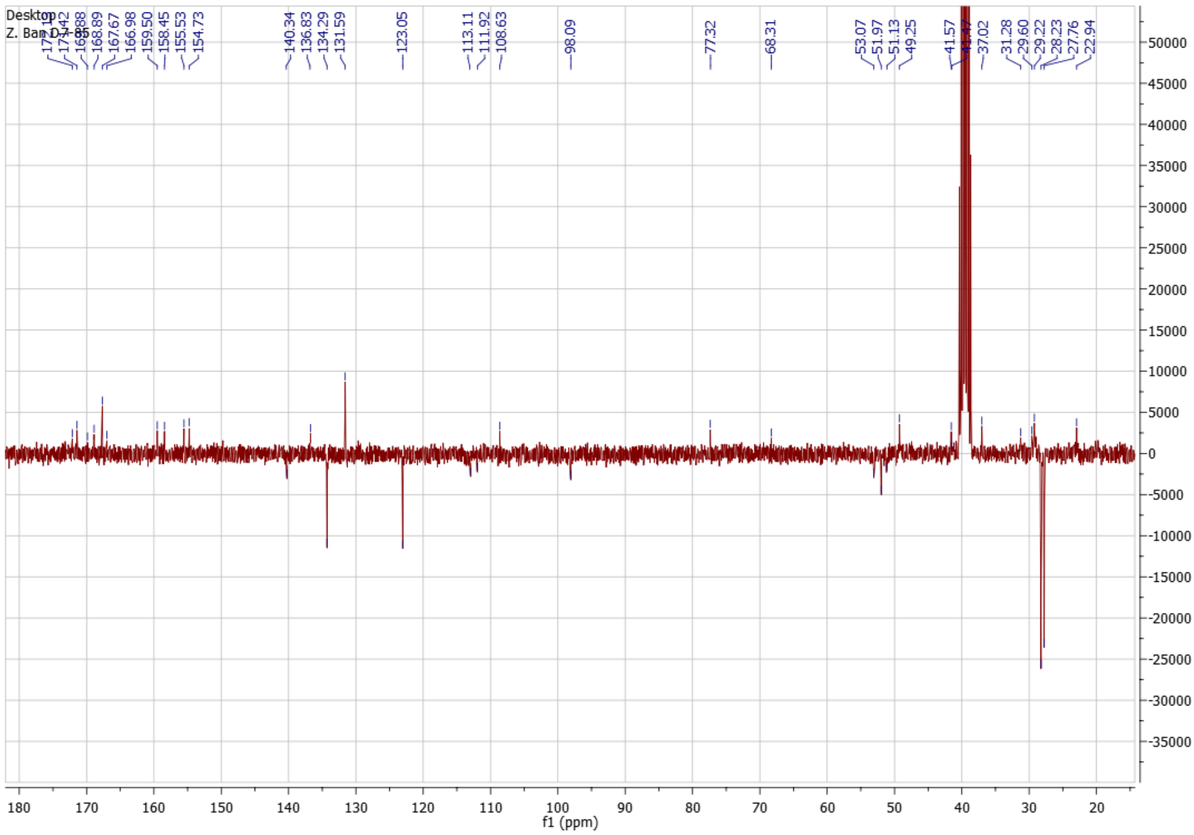

Figure S6 ^{1}H NMR (300 MHz, DMSO-*d*6) and ^{13}C NMR spectra (75 MHz, APT, DMSO-*d*6) of compound **19**.

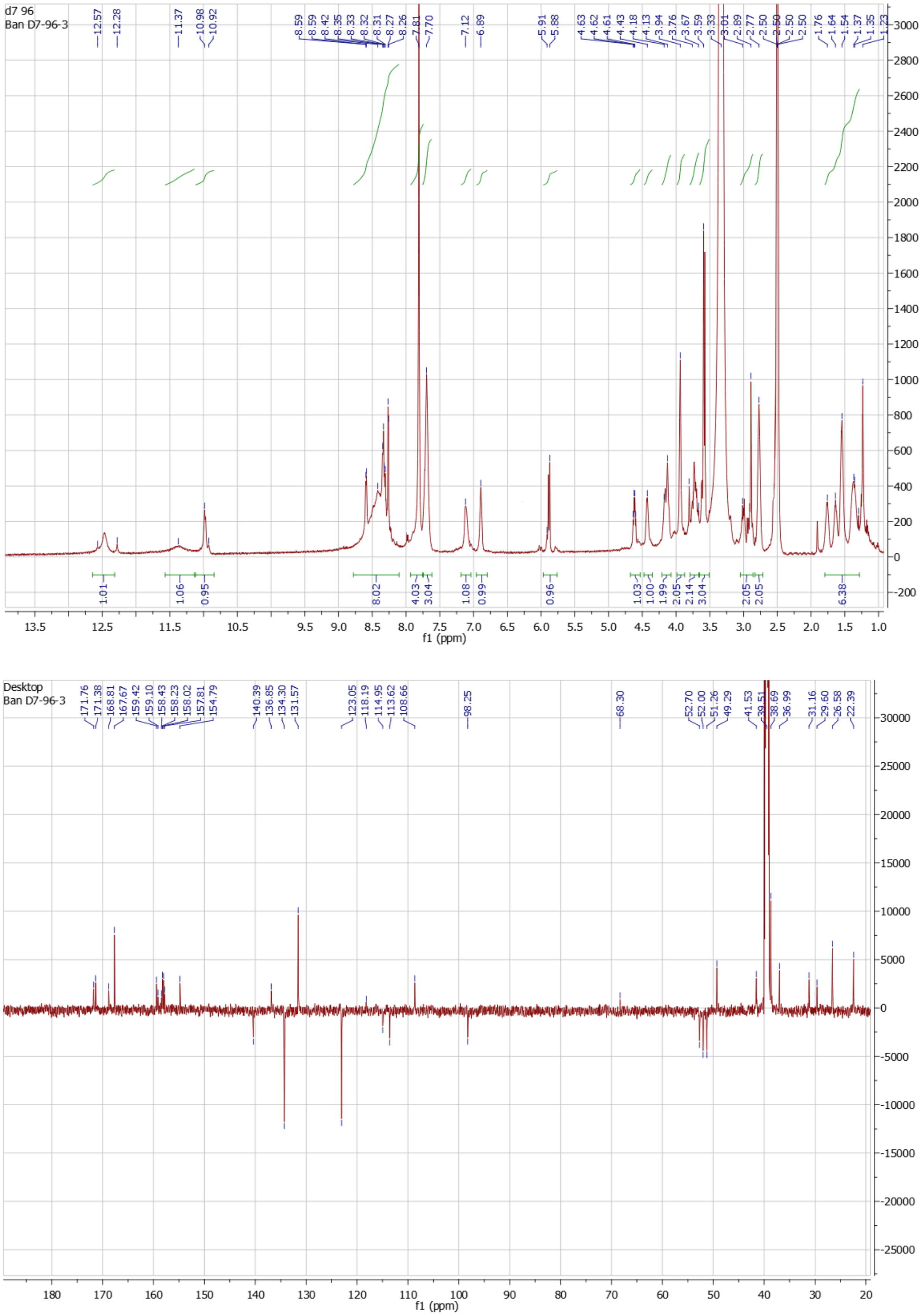

Figure S7 ^{1}H NMR (300 MHz, DMSO-*d*6) and ^{13}C NMR spectra (151 MHz, APT, DMSO-*d*6) of compound **2**.

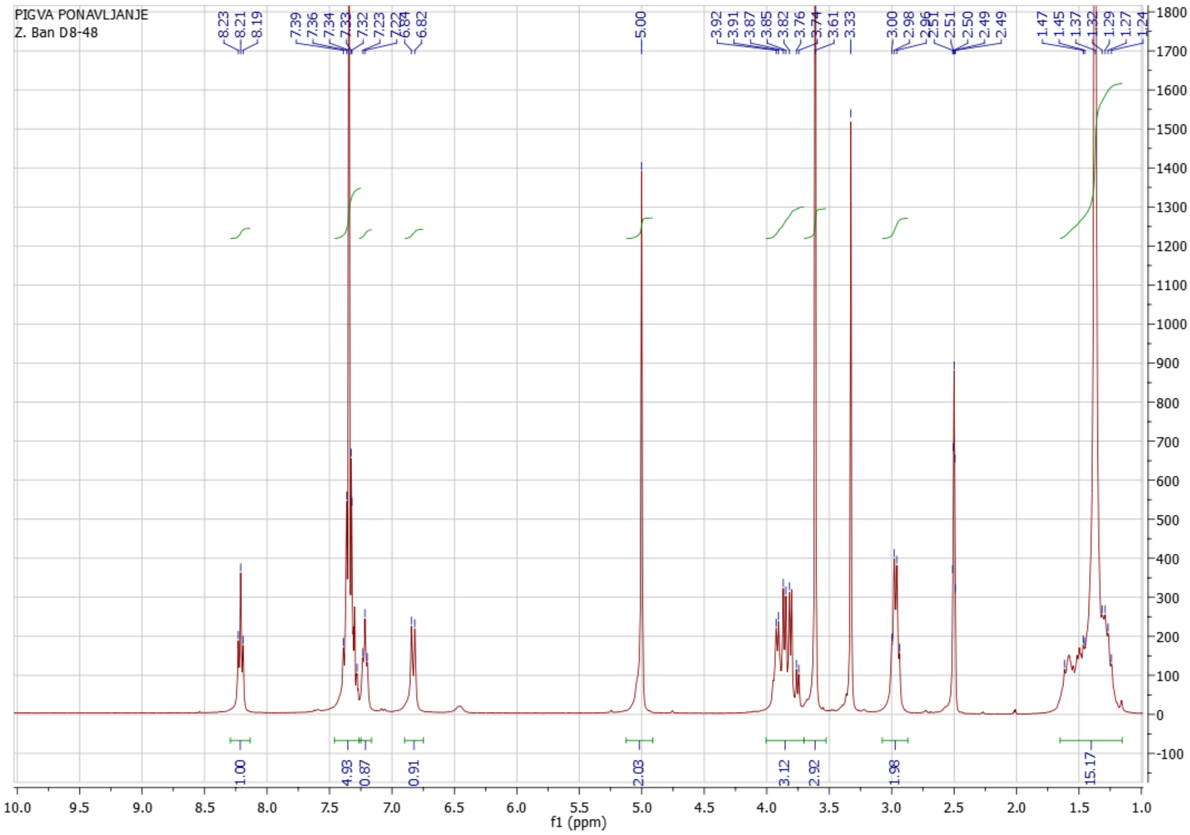

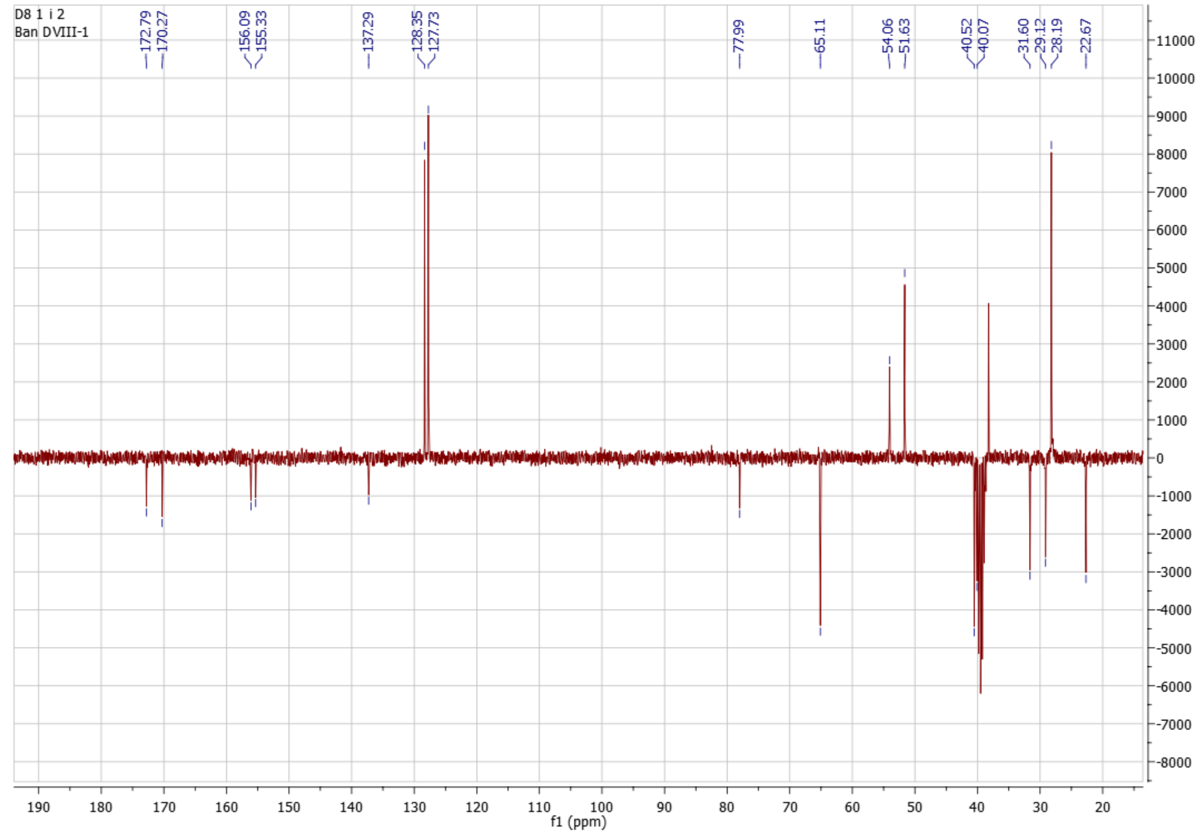

Figure S8 ^{1}H NMR (300 MHz, DMSO-*d*6) and ^{13}C NMR spectra (75 MHz, APT, DMSO-*d*6) of compound **20**.

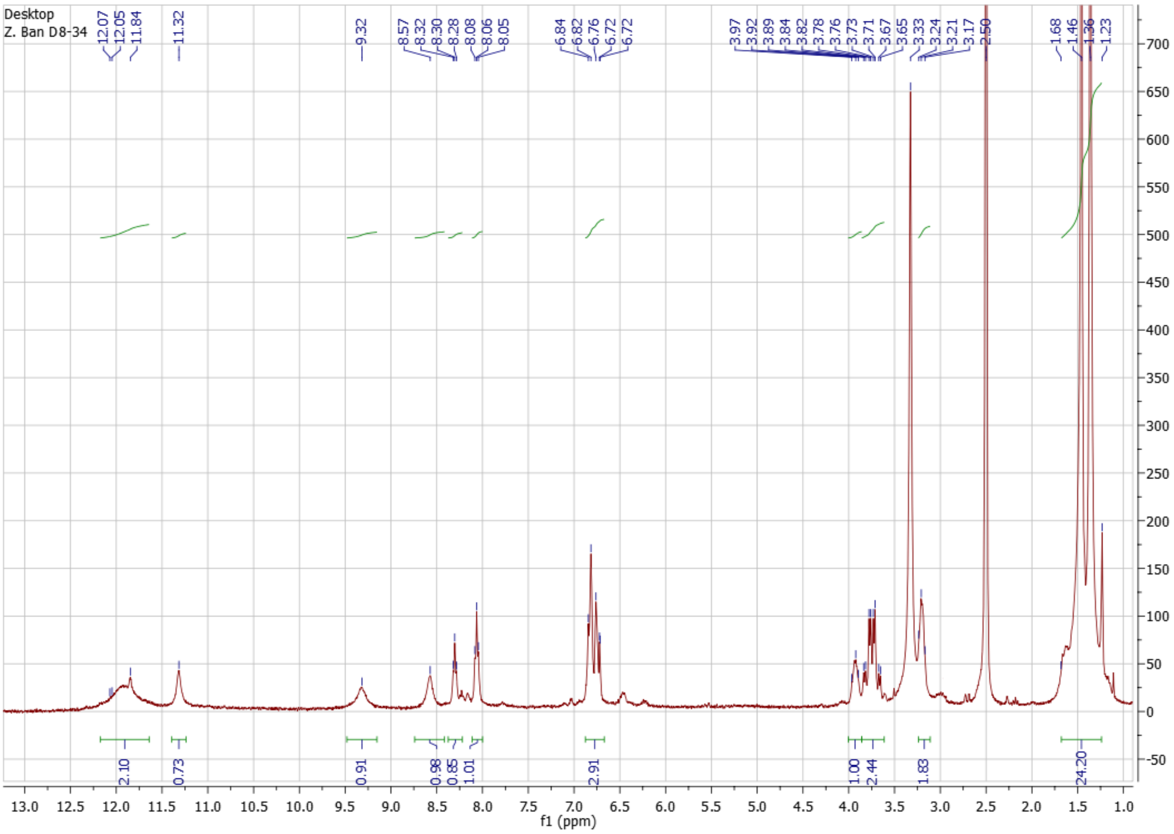

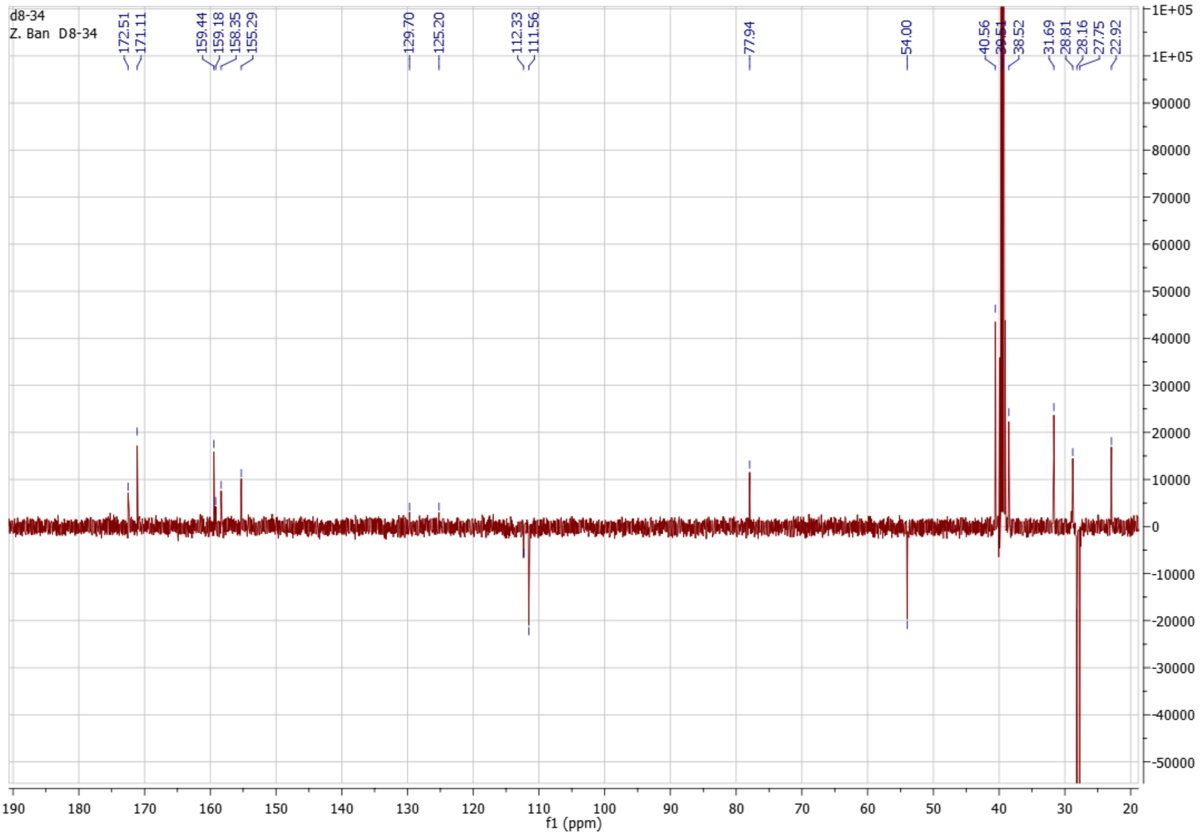

Figure S9 ^{1}H NMR (300 MHz, DMSO-*d*6) and ^{13}C NMR spectra (75 MHz, APT, DMSO-*d*6) of compound **23**.

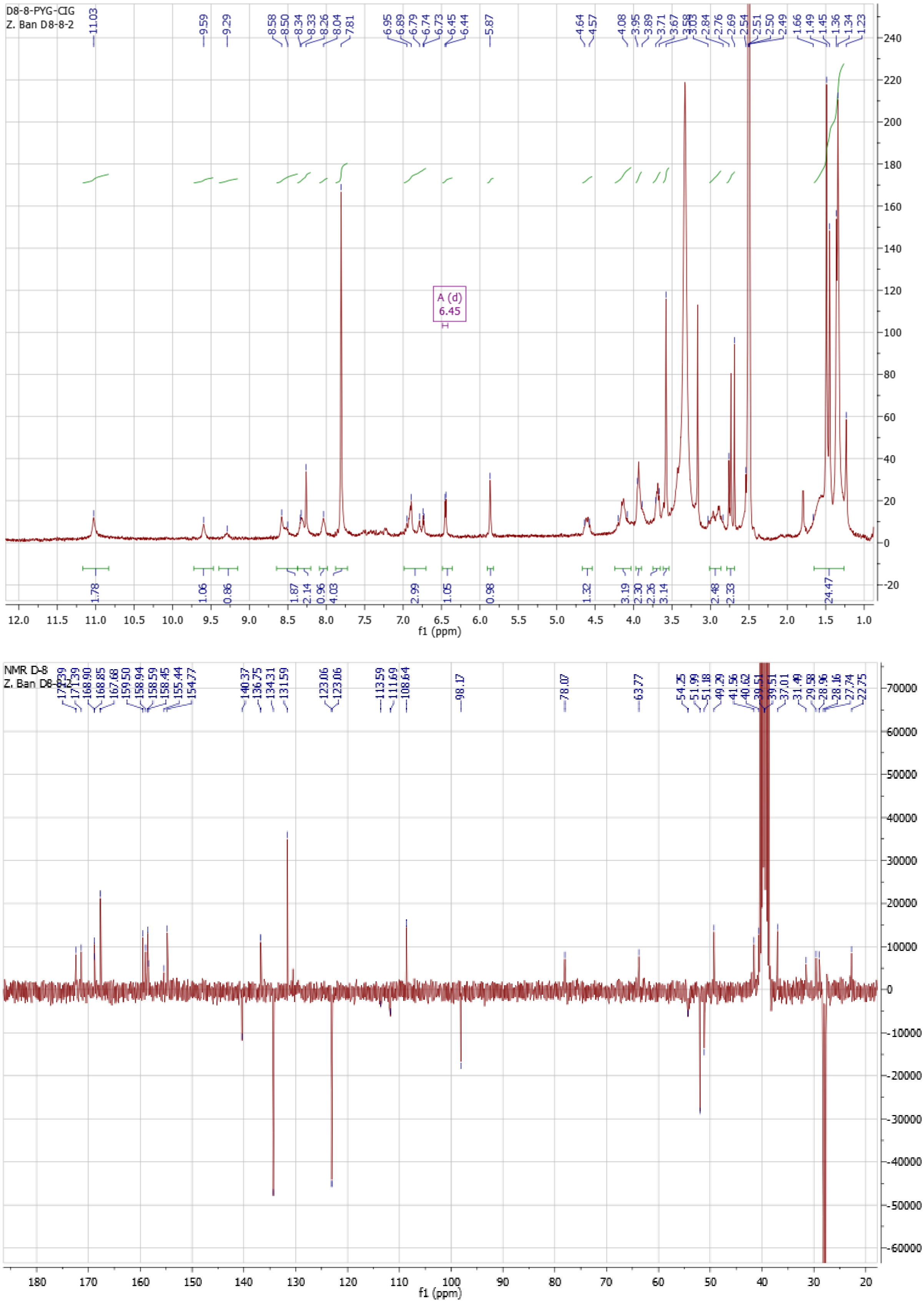

Figure S10 ^{1}H NMR (300 MHz, DMSO-*d*6) and ^{13}C NMR spectra (75 MHz, APT, DMSO-*d*6) of compound **24**

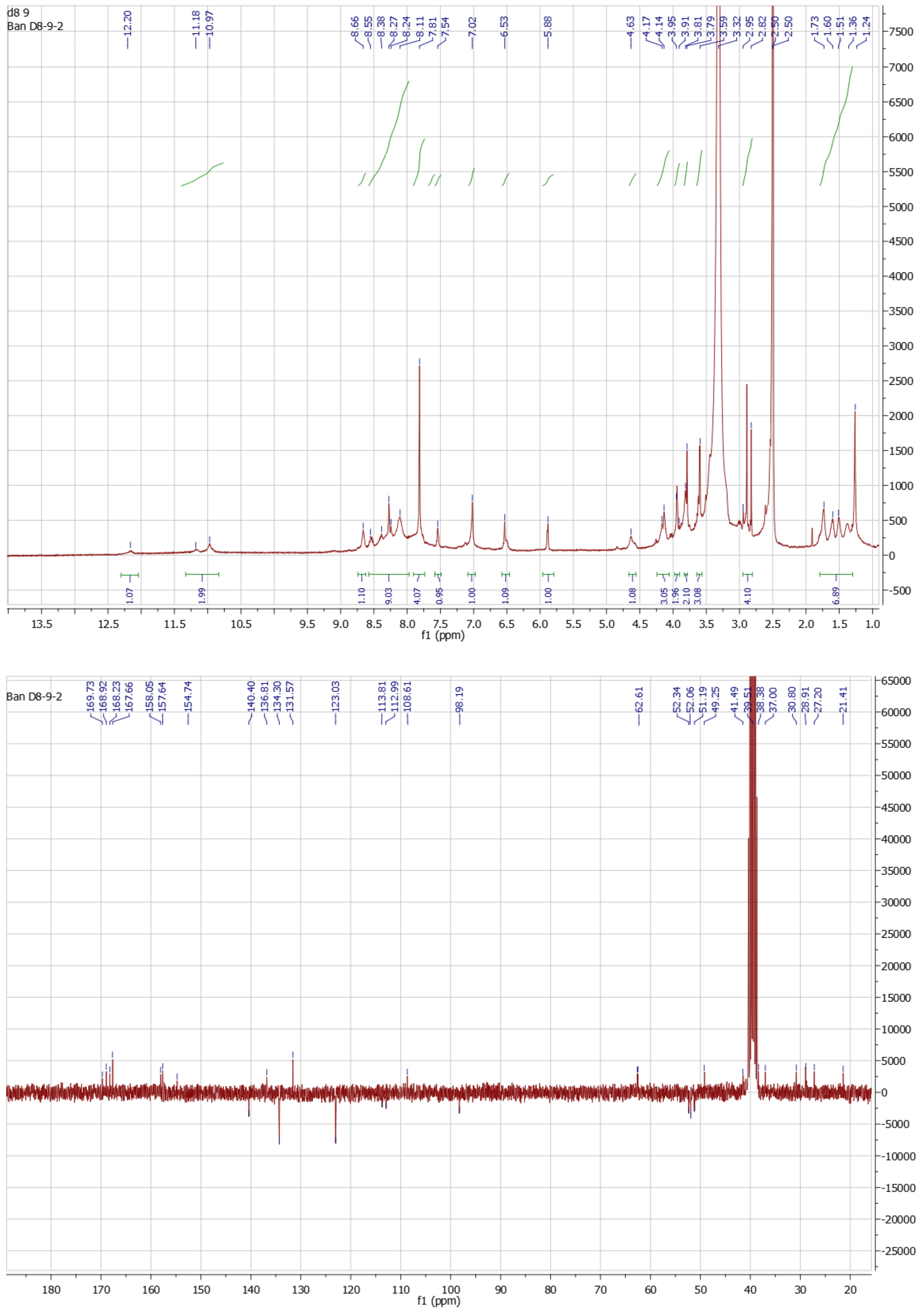

Figure S11. ^{1}H NMR (300 MHz, DMSO-*d*6) and ^{13}C NMR spectra (75 MHz, APT, DMSO-*d*6) of compound **3**.

Spectroscopic experiments

The electronic absorption spectra were obtained on Varian Cary 100 Bio spectrometer and CD spectra on JASCO J815 spectrophotometer all in quartz cuvettes (1 cm). The spectroscopic studies were performed in aqueous buffer solution (pH 7.0 and pH 5.0, sodium cacodylate buffer, I=0.05 mol dm^{-3}). Under the experimental conditions absorbance of **2** and **3** were proportional to its concentration. Polynucleotides were dissolved in sodium cacodylate buffer, I = 0.05 mol dm^{-3}, pH=7. Calf thymus (*ct*-) DNA was additionally sonicated and filtered through a 0.45 µm filter.[1,2] Polynucleotide concentration was determined spectroscopically as the concentration of phosphates. Spectroscopic titrations were performed at pH 7.0 and pH 5.0 (I=0.05 mol dm^{-3}, sodium cacodylate buffer) by adding portions of polynucleotide solution into the solution of the studied compound for UV/Vis experiments and for CD experiments were done by adding portions of compound stock solution into the solution of polynucleotide. Titration data were processed by Scatchard equation. Values for K_s and n all have satisfactory correlation coefficients (>0.999). Thermal melting curves for DNA and RNA and their complexes with studied compounds were determined as previously described[3] by following the absorption change at 260 nm and as a function of temperature. Absorbance of the ligands was subtracted from every curve, and the absorbance scale was normalized. The Tm values are the midpoints of the transition curves, determined from the maximum of the first derivative and checked graphically by the tangent method. ΔTm values were calculated subtracting Tm of the free nucleic acid from Tm of the complex. Every ΔTm value here reported was the average of at least two measurements, the error in ΔTm is ± 0.5 °C.

UV/Vis data:

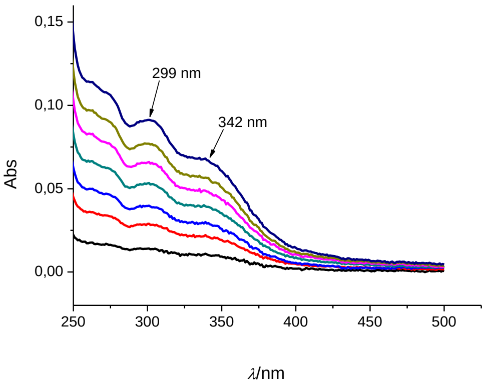

Figure S12. Concentration dependence (concentration range from 3×10^{-6}– 2×10^{-5} mol dm^{-3}) of **1** in buffered solution pH 7, I = 0.05 mol dm^{-3}.

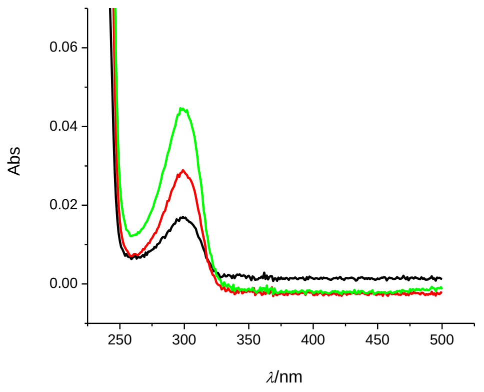

Figure S13. Concentration dependence (concentration range from 6×10^{-6}– 1.8×10^{-5} mol dm^{-3}) of **N-(2-bromoethyl)phtalimide)** in buffered solution pH 7, I = 0.05 mol dm^{-3}.

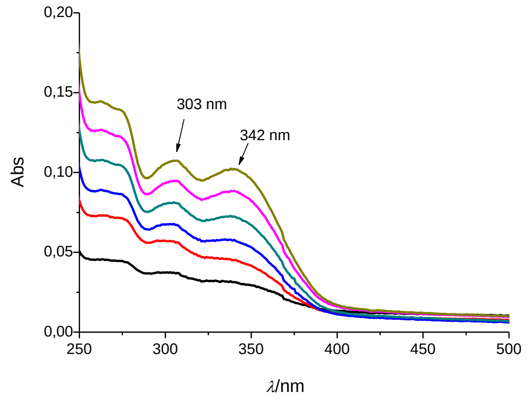

Figure S14. Concentration dependence (concentration range from 3×10^{-6}– 2×10^{-5} mol dm^{-3}) of **12** in buffered solution pH 7, I = 0.05 mol dm^{-3}.

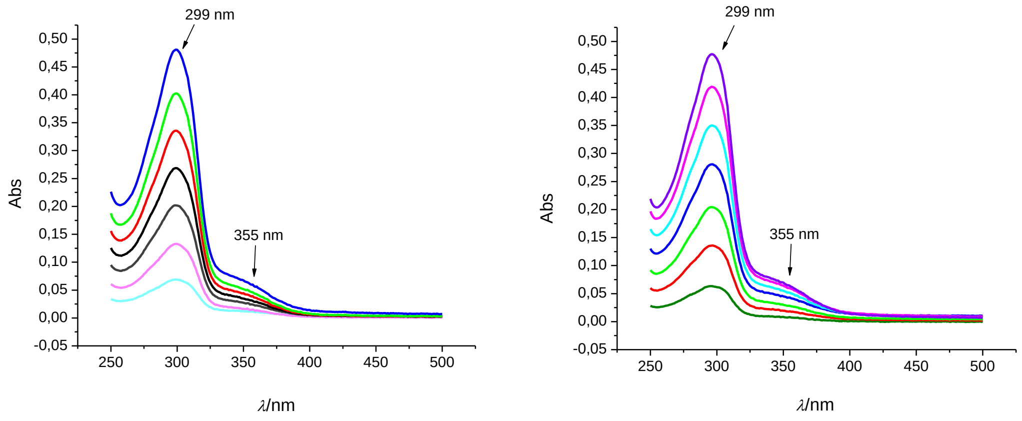

Figure S15. Concentration dependence (concentration range from 3×10^{-6}– 2×10^{-5} mol dm^{-3}) of **2** in different buffered solution (LEFT: pH 7.0 and RIGHT: pH 5.0, I = 0.05 mol dm^{-3}).

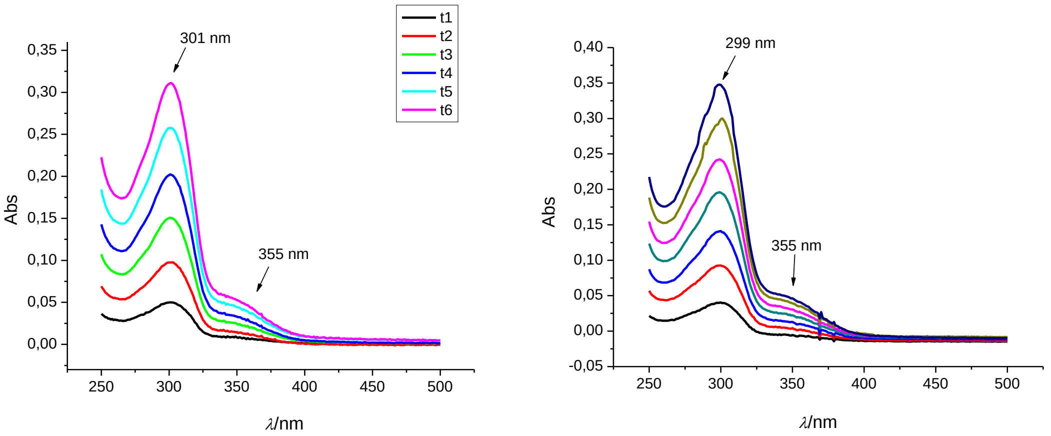

Figure S16. Concentration dependence (concentration range from 3×10^{-6}– 2×10^{-5} mol dm^{-3}) of **3** in different buffered solution (LEFT: pH 7.0 and RIGHT: pH 5.0, I = 0.05 mol dm^{-3}).

Table S1. Electronic absorbance data of **GCP**, **1, 2**, **3** and **12**

Compound	pH	λ_{max}/nm	$\varepsilon\times10^3$/mmol^{-1} cm^2
**GCP**	7	299	16.87
**1**	7	299	4.3±0.1
		342	3.1±0.1
**2**	7	299	22.9±0.3
		355	2.7±0.2
**2**	5	299	23.3±0.3
		355	3.3±0.2
**3**	7	301	17.5±0.2
		355	2.8±0.1
**3**	5	299	17.2±0.1
		355	2.8±0.1
**12**	7	303	4.2±0.2
		342	4.4±0.1

Stock solutions of compounds **1, 2**, **3** and **12** were prepared in dimethyl sulfoxide (about c = 3×10^{-3} mol dm^{-3}).

Fluorimetric spectra:

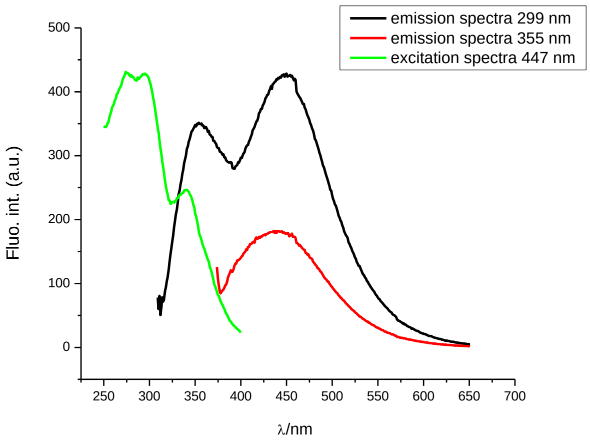

Figure S17. Excitation (▬λ_{em} = 447 nm) and emission (λ_{exc} = 299 nm ▬, λ_{exc} = 355 nm ▬) spectra of **2**. Done at pH 5.0, sodium cacodylate buffer, I = 0.05 M.

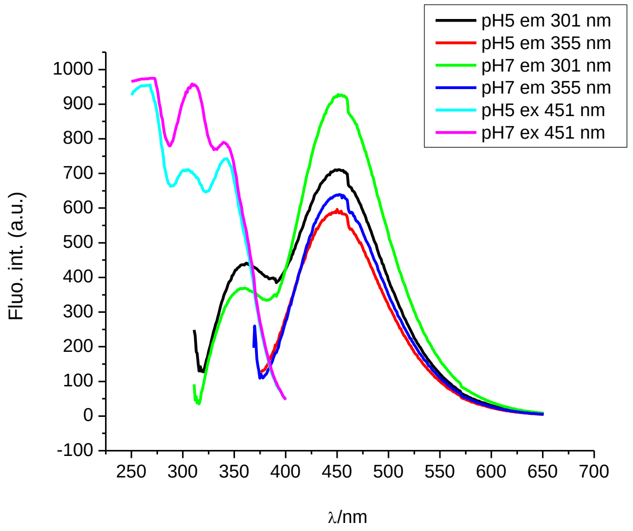

Figure S18. Comparison of emission and excitation spectra of **3** in buffer sodium cacodylate (pH 5.0 and pH 7.0, I = 0.05 mol dm^{-3}). Compound is protonated at pH 5.0: excitation spectra have different forms.

Fluorimetric titrations of compounds 2 and 3 with DNA/RNA:

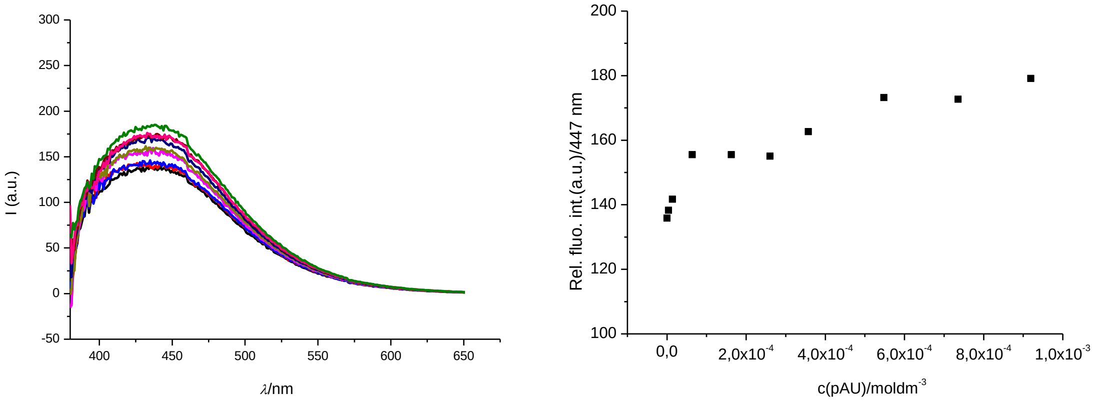

Figure S19. Fluorimetric titration of **2** (c = 2×10^{-6} mol dm^{-3}; λ_{exc} = 355 nm) with **poly A – poly U**. RIGHT: dependence of fluorescence at λ_{max}= 447 nm on c(RNA). **Too small** changes didn't allow accumulation of enough data points for accurate analysis by Scatchard eq. Done at pH 7, sodium cacodylate buffer, I = 0.05 mol dm^{-3}.

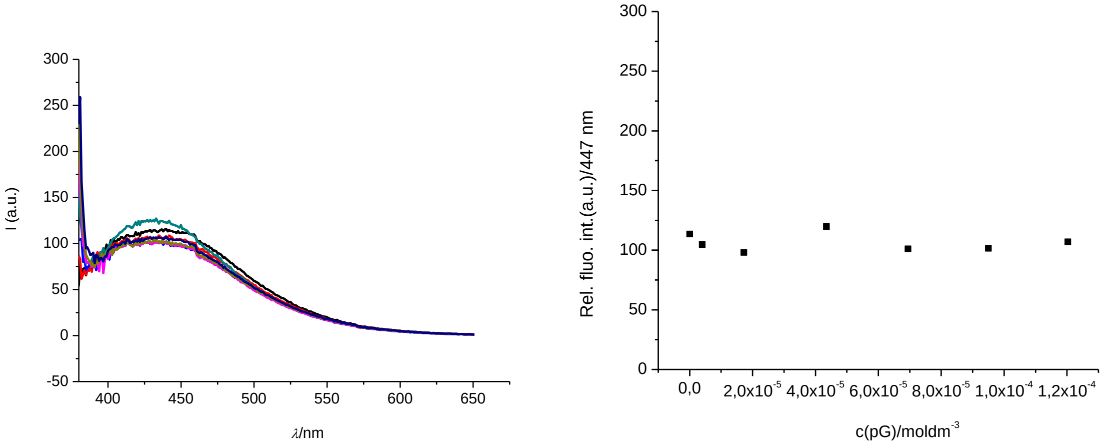

Figure S20. Fluorimetric titration of **2** (c = 2×10^{-6} mol dm^{-3}; λ_{exc}= 355 nm) with **poly G**. RIGHT: dependence of fluorescence at λ_{max} = 447 nm on c(RNA). **Too small** changes didn't allow accumulation of enough data points for accurate analysis by Scatchard eq. Done at pH 7, sodium cacodylate buffer, I = 0.05 mol dm^{-3}.

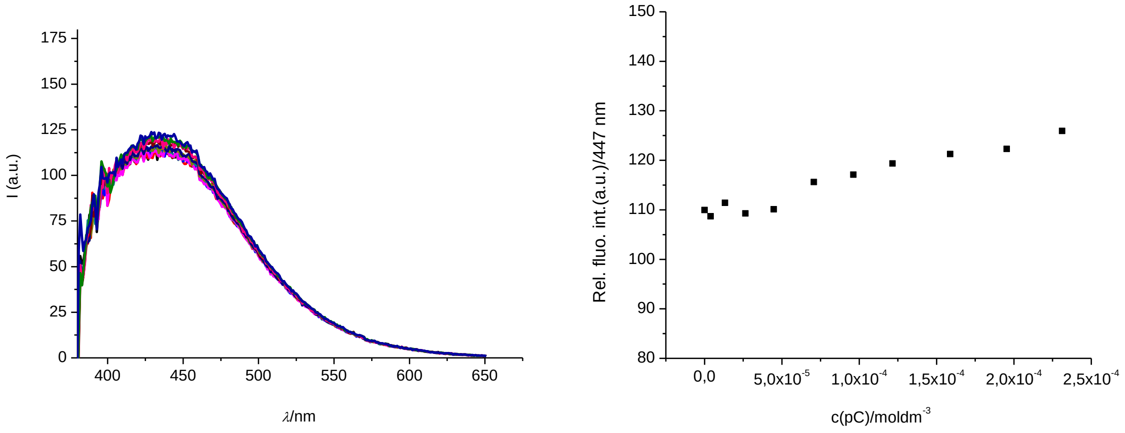

Figure S21. Fluorimetric titration of **2** (c = 2×10^{-6} mol dm^{-3}; λ_{exc} = 355 nm) with **poly C**. RIGHT: dependence of fluorescence at λ_{max} = 447 nm on c(RNA). **Too small** changes didn't allow accumulation of enough data points for accurate analysis by Scatchard eq. Done at pH 7, sodium cacodylate buffer, I = 0.05 mol dm^{-3}.

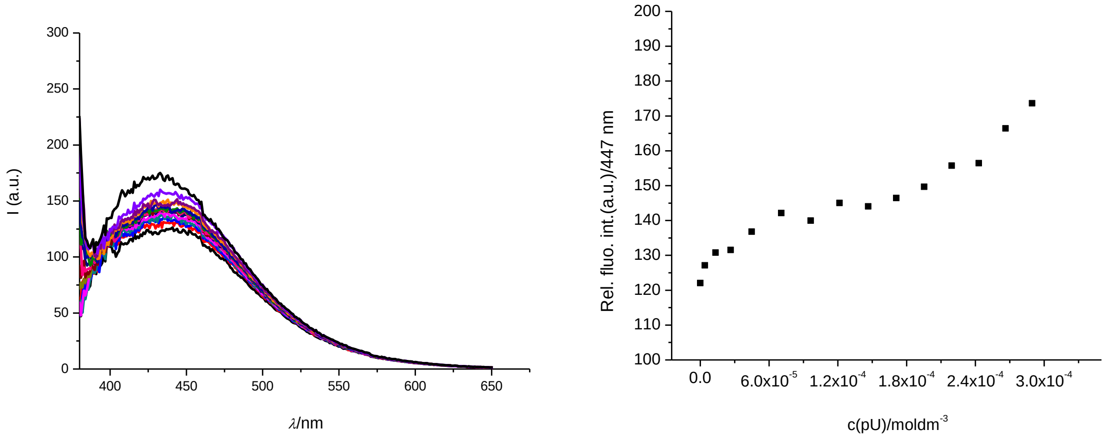

Figure S22. Fluorimetric titration of **2** (c = 2×10^{-6} mol dm^{-3}; λ_{exc}= 355 nm) with **poly U**. RIGHT: dependence of fluorescence at λ_{max} = 447 nm on c(RNA). **Too small** changes didn't allow accumulation of enough data points for accurate analysis by Scatchard eq. Done at pH 7, sodium cacodylate buffer, I = 0.05 mol dm^{-3}.

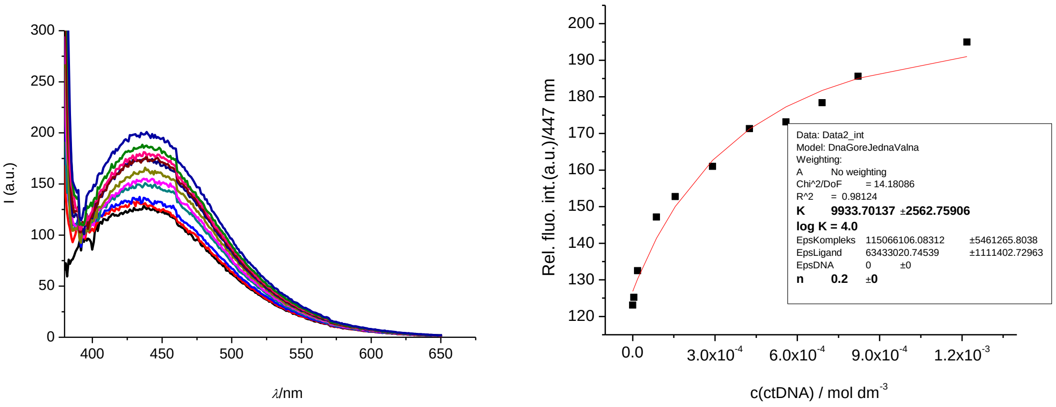

Figure S23. Fluorimetric titration of **2** (c = 2×10^{-6} mol dm^{-3}; λ_{exc}= 355 nm) with **ctDNA**. RIGHT: dependence of fluorescence at λ_{max} = 447 nm on c(DNA), red line is non-linear least square fitting of Scatchard eq. (McGhee, vonHippel formalism) on experimental data. Done at pH 5, sodium cacodylate buffer, I = 0.05 mol dm^{-3}.

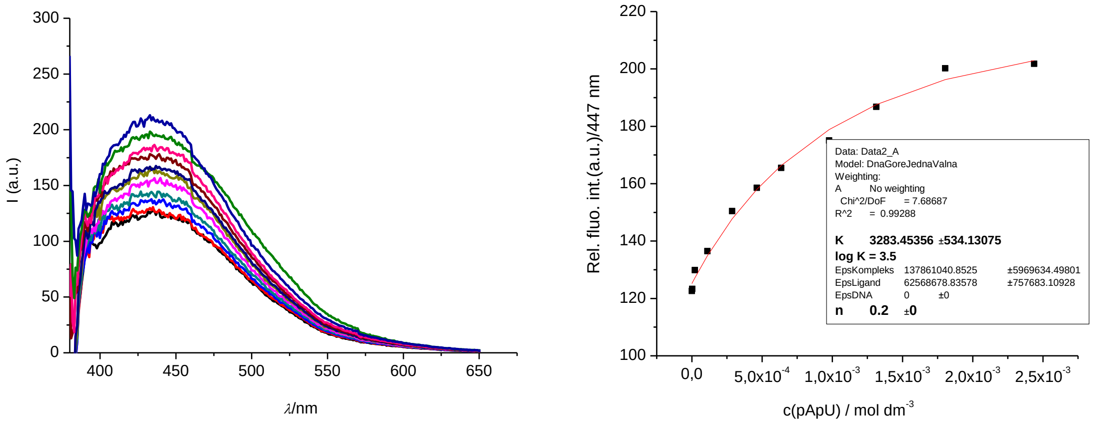

Figure S24. Fluorimetric titration of **2** (c = 2×10^{-6} mol dm^{-3}; λ_{exc} = 355 nm) with **poly A – polyU**. RIGHT: dependence of fluorescence at λ_{max} = 447 nm on c(RNA), red line is non-linear least square fitting of Scatchard eq. (McGhee, vonHippel formalism) on experimental data. Done at pH 5, sodium cacodylate buffer, I = 0.05 mol dm^{-3}.

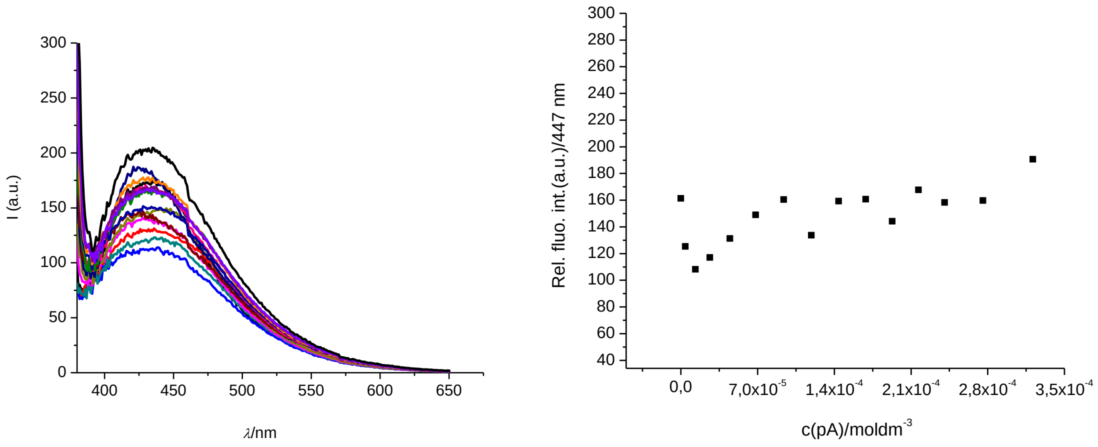

Figure S25. Fluorimetric titration of **2** (c = 2×10^{-6} mol dm^{-3}; λ_{exc} = 355 nm) with **poly A.** RIGHT: dependence of fluorescence at λ_{max} = 447 nm on c(RNA). **Too small** changes didn't allow accumulation of enough data points for accurate analysis by Scatchard eq. Done at pH 5, sodium cacodylate buffer, I = 0.05 mol dm^{-3}.

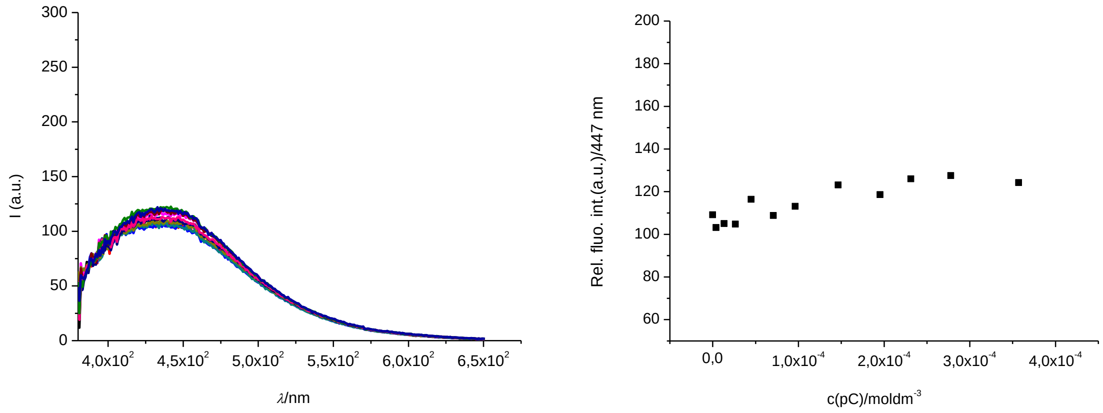

Figure S26. Fluorimetric titration of **2** (c = 2×10^{-6} mol dm^{-3}; λ_{exc} = 355 nm) with **poly C.** RIGHT: dependence of fluorescence at λ_{max} = 447 nm on c(RNA). **Too small** changes didn't allow accumulation of enough data points for accurate analysis by Scatchard eq. Done at pH 5, sodium cacodylate buffer, I = 0.05 mol dm^{-3}.

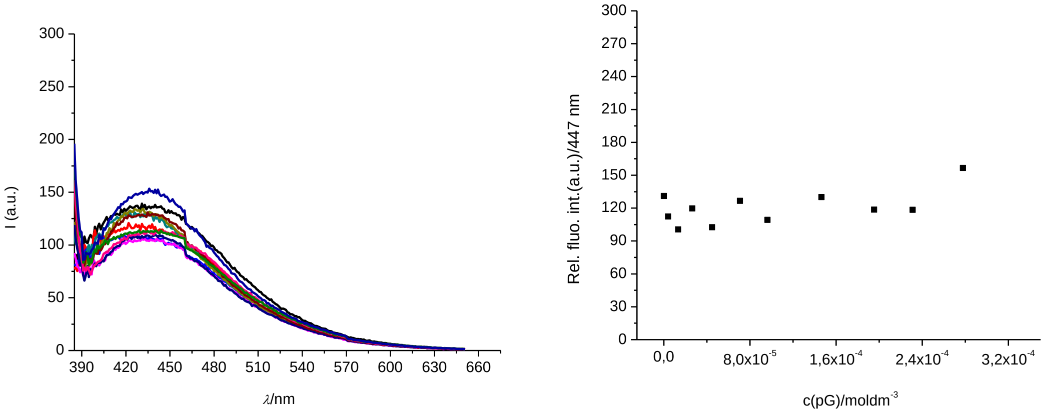

Figure S27. Fluorimetric titration of **2** (c = 2×10^{-6} mol dm^{-3}; λ_{exc} = 355 nm) with **poly G.** RIGHT: dependence of fluorescence at λ_{max} = 447 nm on c(RNA). **Too small** changes didn't allow accumulation of enough data points for accurate analysis by Scatchard eq. Done at pH 5, sodium cacodylate buffer, I = 0.05 mol dm^{-3}.

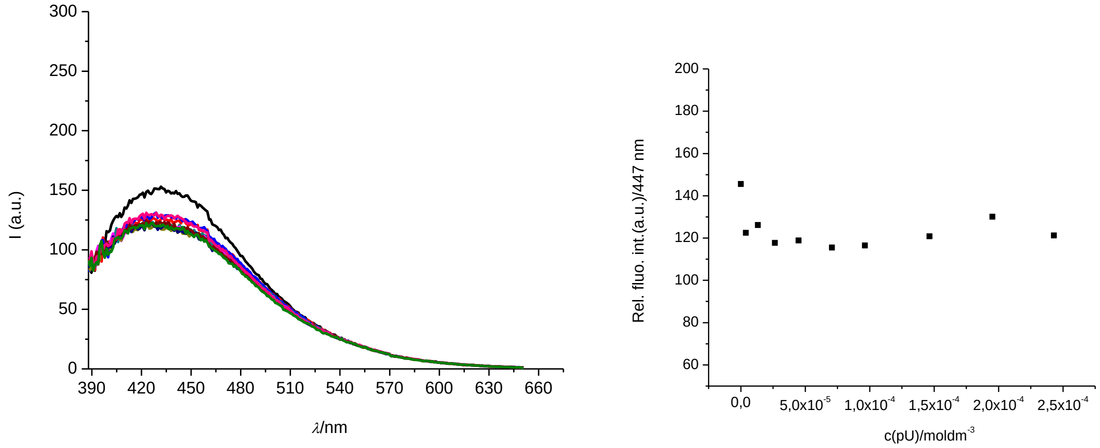

Figure S28. Fluorimetric titration of **2** (c = 2×10^{-6} mol dm^{-3}; λ_{exc} = 355 nm) with **poly U.** RIGHT: dependence of fluorescence at λ_{max} = 447 nm on c(RNA). **Too small** changes didn't allow accumulation of enough data points for accurate analysis by Scatchard eq. Done at pH 5, sodium cacodylate buffer, I = 0.05 mol dm^{-3}.

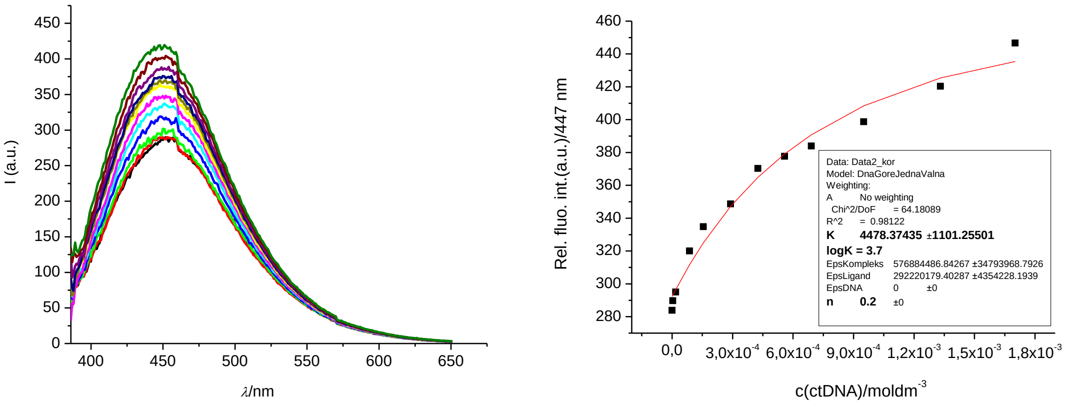

Figure S29. Fluorimetric titration of **3** (c = 2×10^{-6} mol dm^{-3}; λ_{exc} = 355 nm) with **ctDNA**. RIGHT: dependence of fluorescence at λ_{max} = 447 nm on c(DNA), red line is non-linear least square fitting of Scatchard eq. (McGhee, vonHippel formalism) on experimental data. Done at pH 7, sodium cacodylate buffer, I = 0.05 mol dm^{-3}.

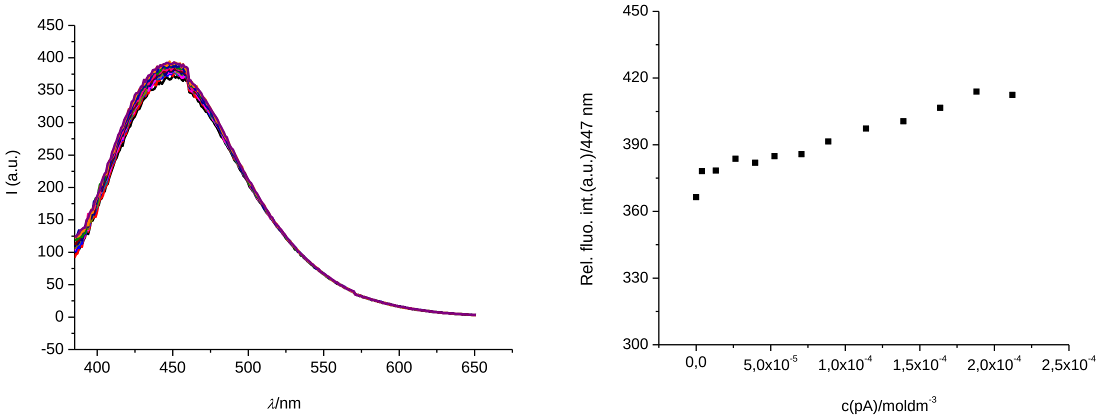

Figure S30. Fluorimetric titration of **3** (c = 2×10^{-6} mol dm^{-3}; λ_{exc} = 355 nm) with **poly A.** RIGHT: dependence of fluorescence at λ_{max} = 447 nm on c(RNA). **Too small** changes didn't allow accumulation of enough data points for accurate analysis by Scatchard eq. Done at pH 7, sodium cacodylate buffer, I = 0.05 mol dm^{-3}.

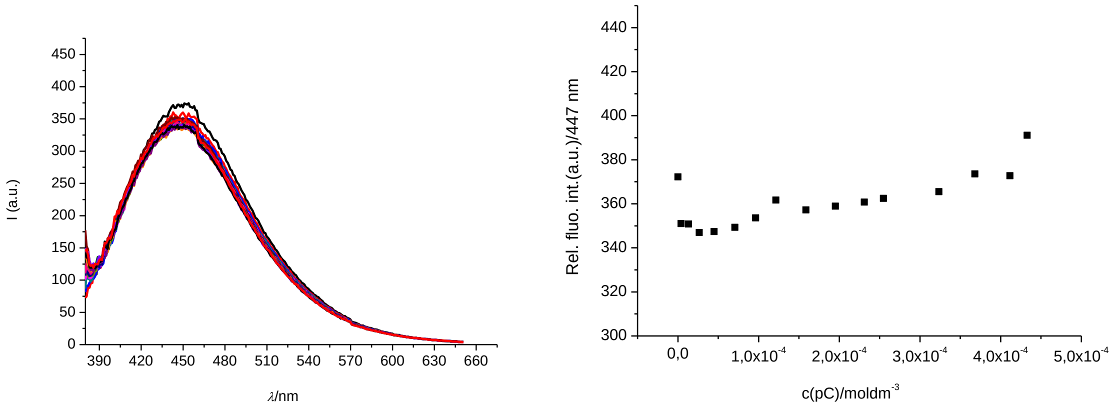

Figure S31. Fluorimetric titration of **3** (c = 2×10^{-6} mol dm^{-3}; λ_{exc}= 355 nm) with **poly C.** RIGHT: dependence of fluorescence at λ_{max} = 447 nm on c(RNA). **Too small** changes didn't allow accumulation of enough data points for accurate analysis by Scatchard eq. Done at pH 7, sodium cacodylate buffer, I = 0.05 mol dm^{-3}.

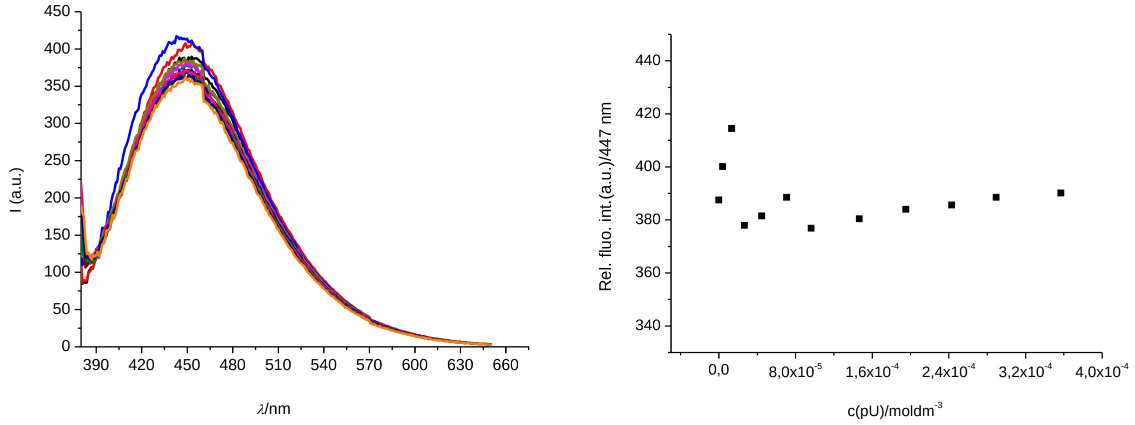

Figure S32. Fluorimetric titration of **3** (c = 2×10^{-6} mol dm^{-3}; λ_{exc} = 355 nm) with **poly U.** RIGHT: dependence of fluorescence at λ_{max} = 447 nm on c(RNA). **Too small** changes didn't allow accumulation of enough data points for accurate analysis by Scatchard eq. Done at pH 7, sodium cacodylate buffer, I = 0.05 mol dm^{-3}

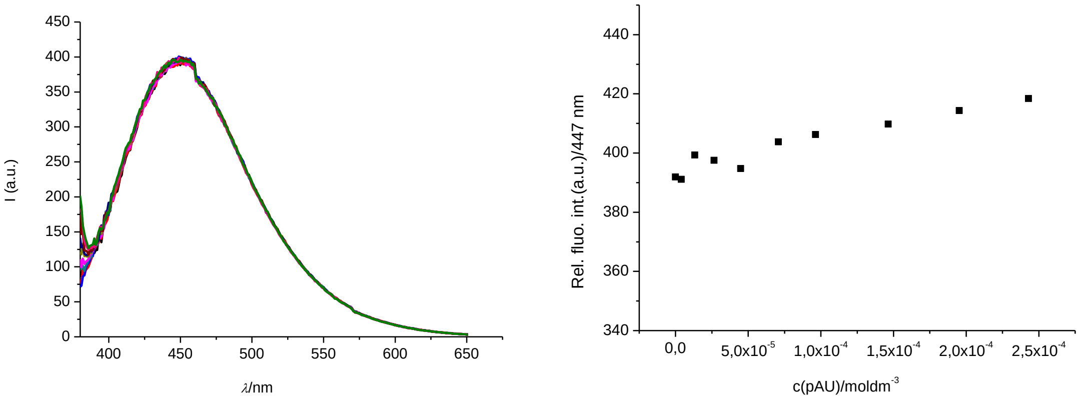

Figure S33. Fluorimetric titration of **3** (c = 2×10^{-6} mol dm^{-3}; λ_{exc} = 355 nm) with **poly A - poly U**. RIGHT: dependence of fluorescence at λ_{max} = 447 nm on c(RNA). **Too small** changes didn't allow accumulation of enough data points for accurate analysis by Scatchard eq. Done at pH 7, sodium cacodylate buffer, I = 0.05 mol dm^{-3}

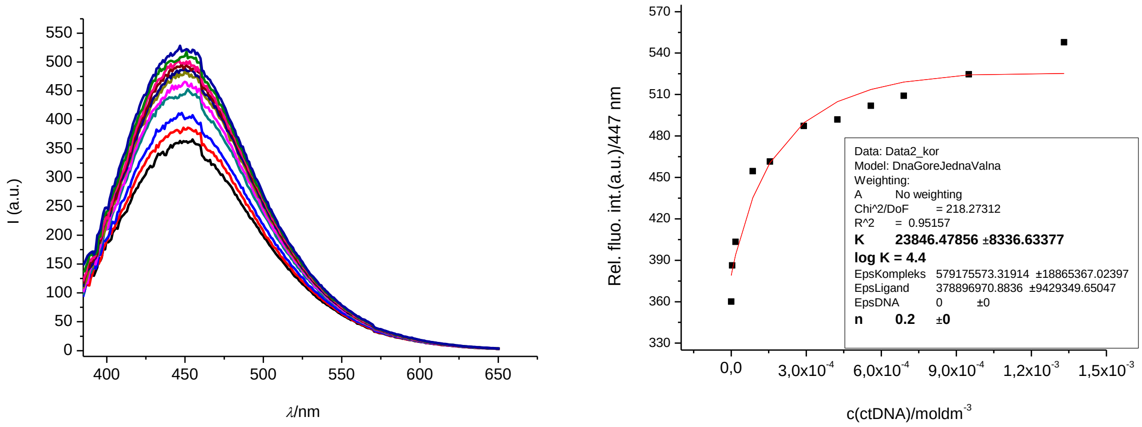

Figure S34. Fluorimetric titration of **3** (c = 2×10^{-6} mol dm^{-3}; λ_{exc} = 355 nm) with **ctDNA**. RIGHT: dependence of fluorescence at λ_{max} = 447 nm on c(DNA), red line is non-linear least square fitting of Scatchard eq. (McGhee, vonHippel formalism) on experimental data. Done at pH 5, sodium cacodylate buffer, I = 0.05 mol dm^{-3}.

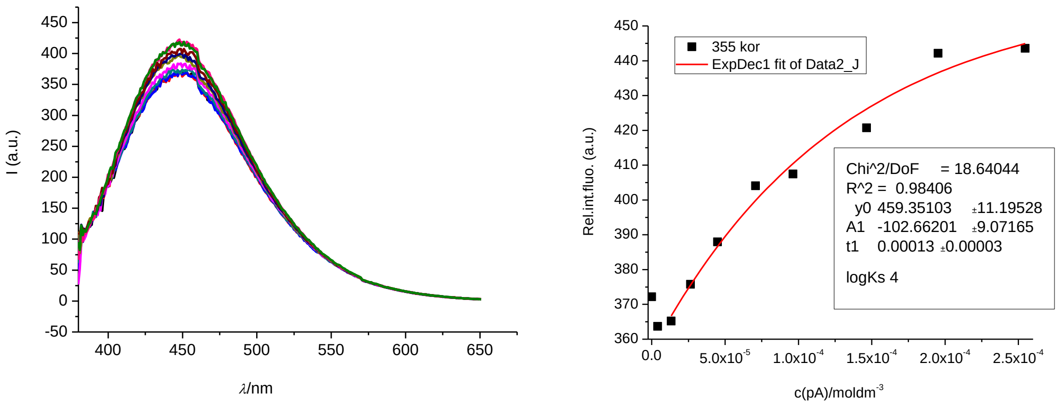

Figure S35. Fluorimetric titration of **3** (c = 2×10^{-6} mol dm^{-3}; λ_{exc} = 355 nm) with **poly A.** RIGHT: dependence of fluorescence at λ_{max} = 447 nm on c(RNA). Done at pH 5, sodium cacodylate buffer, I = 0.05 mol dm^{-3}

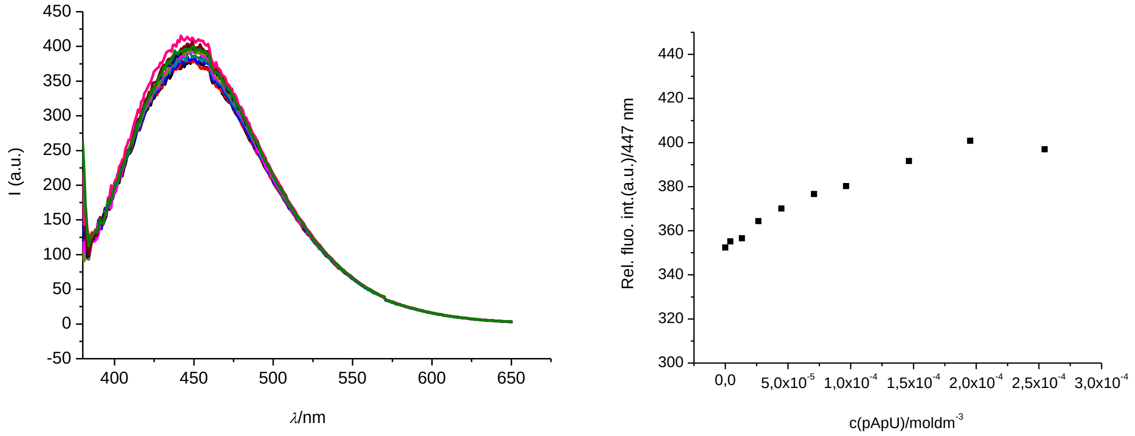

Figure S36. Fluorimetric titration of **3** (c = 2×10^{-6} mol dm^{-3}; λ_{exc} = 355 nm) with **poly A-poly U** RIGHT: dependence of fluorescence at λ_{max} = 447 nm on c(RNA). **Too small** changes didn't allow accumulation of enough data points for accurate analysis by Scatchard eq. Done at pH 5, sodium cacodylate buffer, I = 0.05 mol dm^{-3}

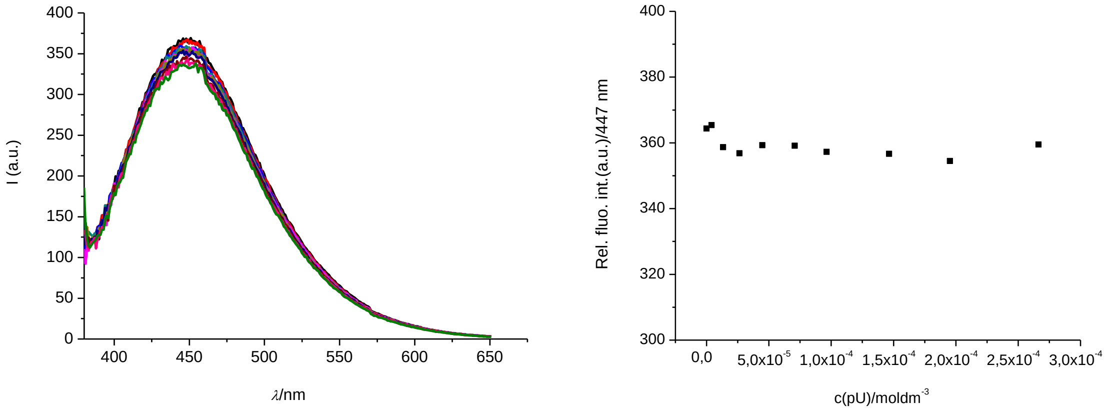

Figure S37. Fluorimetric titration of **3** (c = 2×10^{-6} mol dm^{-3}; λ_{exc} = 355 nm) with of **poly U** RIGHT: dependence of fluorescence at λ_{max} = 447 nm on c(RNA). **Too small** changes didn't allow accumulation of enough data points for accurate analysis by Scatchard eq. Done at pH 5, sodium cacodylate buffer, I = 0.05 mol dm^{-3}

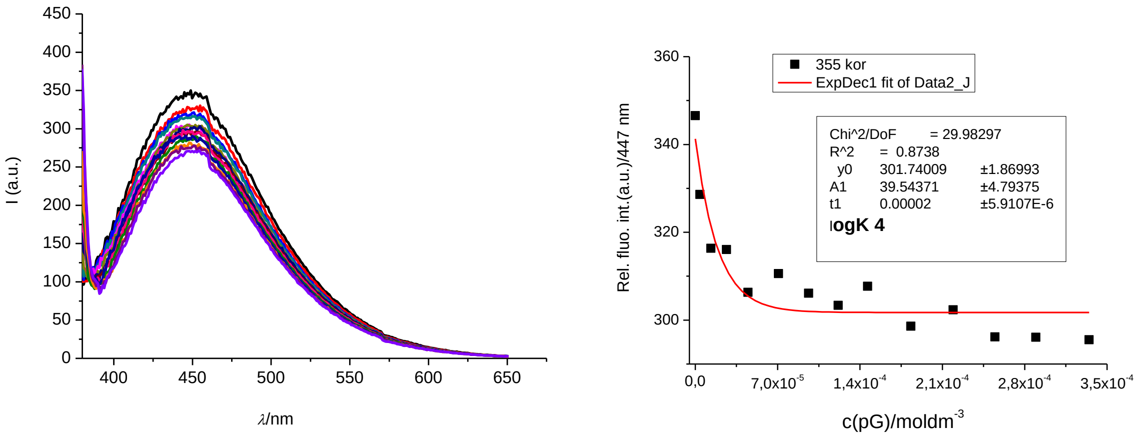

Figure S38. Fluorimetric titration of **3** (c = 2×10^{-6} mol dm^{-3}; λ_{exc} = 355 nm) with of **poly G.** RIGHT: dependence of fluorescence at λ_{max}= 447 nm on c(RNA). Done at pH 5, sodium cacodylate buffer, I = 0.05 mol dm^{-3}

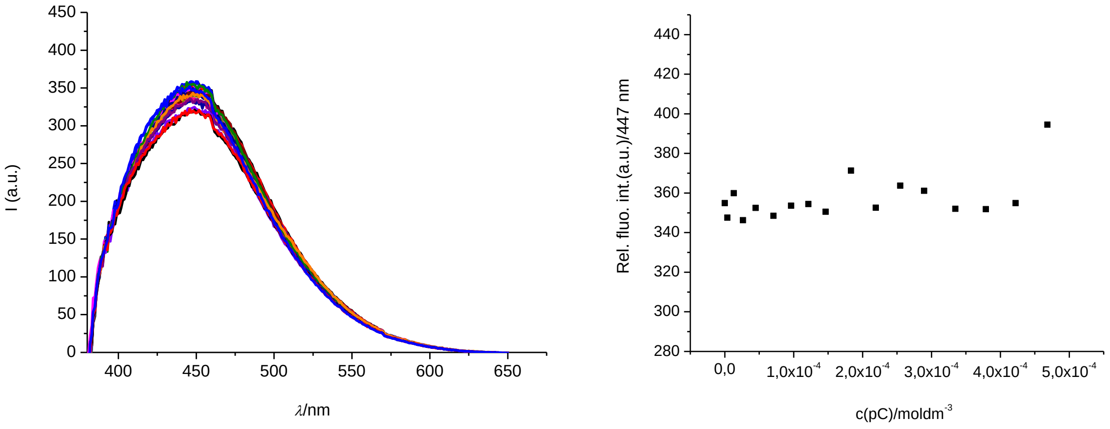

Figure S39. Fluorimetric titration of **3** (c = 2×10^{-6} mol dm^{-3}; λ_{exc} = 355 nm) with of **poly C.** RIGHT: dependence of fluorescence at λ_{max} = 447 nm on c(RNA). **Too small** changes didn't allow accumulation of enough data points for accurate analysis by Scatchard eq. Done at pH 5, sodium cacodylate buffer, I = 0.05 mol dm^{-3}

Fluorimetric titrations of compounds 1 and 12 with DNA/RNA:

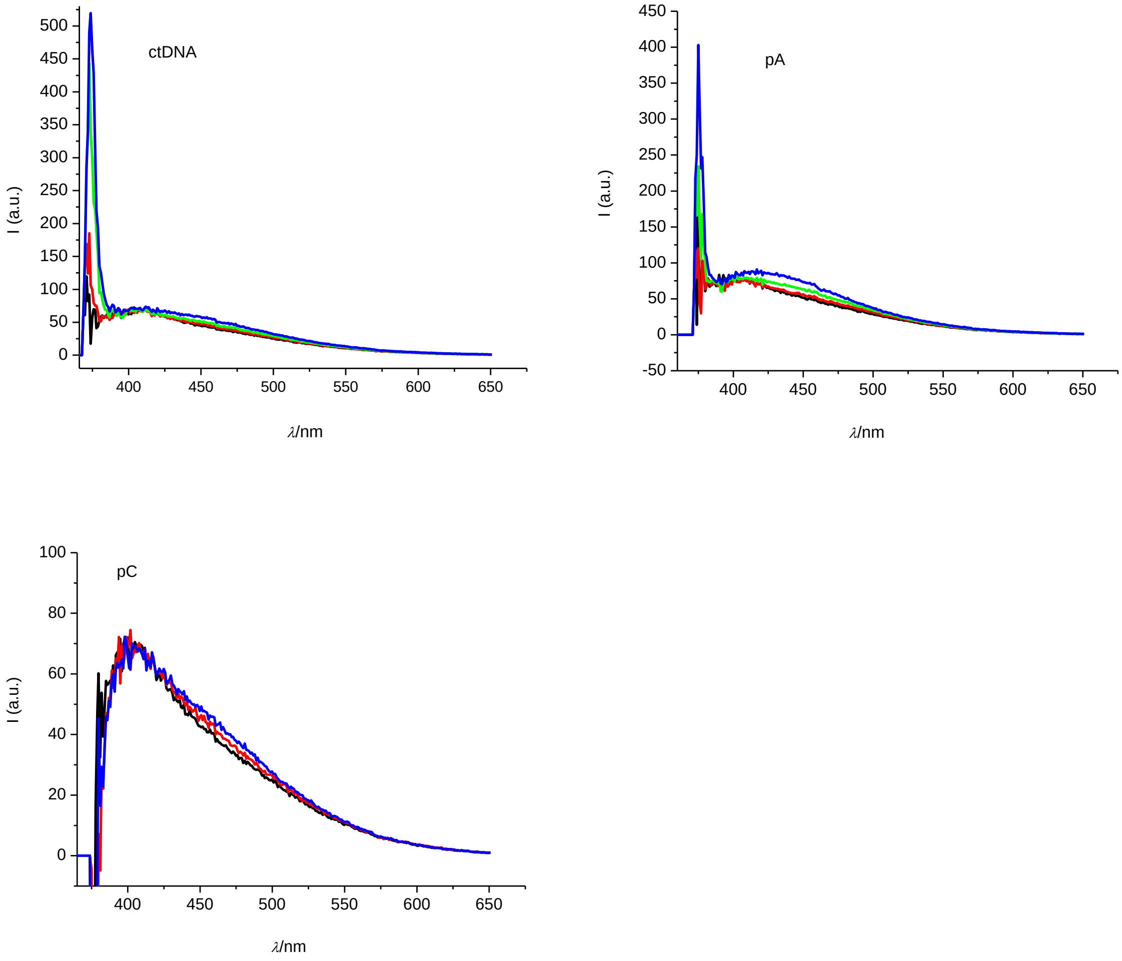

Figure S40. Fluorescence spectra changes of **1** (c = 2×10^{-6} mol dm^{-3}) upon addition of **ctDNA**, **poly A** and **poly C** (concentration range from 2×10^{-5}–2×10^{-4} mol dm^{-3}; λ_{exc} = 355 nm) at pH 7.0, sodium cacodylate buffer, I = 0.05 M. **Too small** changes didn't allow accumulation of enough data points for accurate analysis by Scatchard eq.

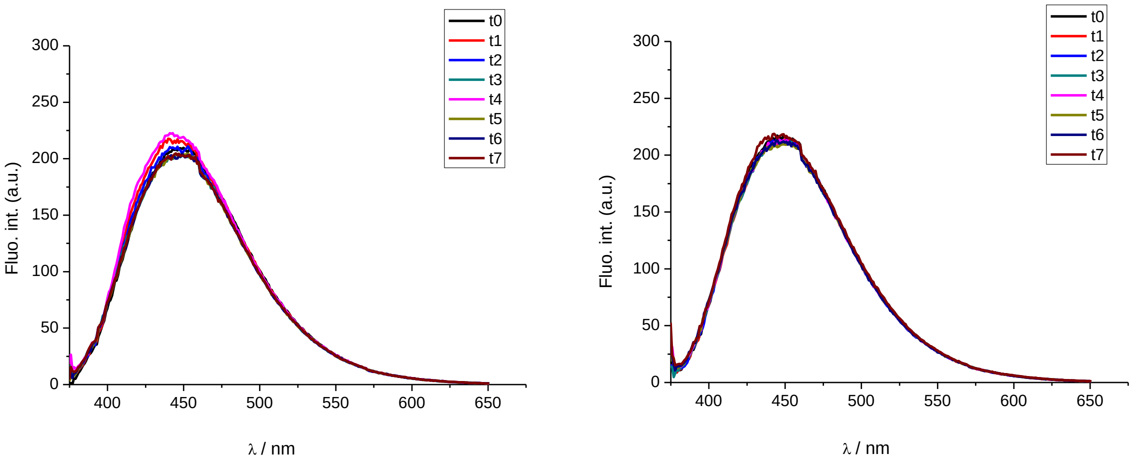

Figure S41. Fluorescence spectra changes of **12** (c= 2×10^{-6} mol dm^{-3}) upon addition of **poly A** (concentration range from 4×10^{-6}–7×10^{-5} mol dm^{-3}) at pH 7.0 (LEFT) and pH 5.0 (RIGHT), sodium cacodylate buffer, I=0.05 M, λ_{ex} = 355 nm

CD results:

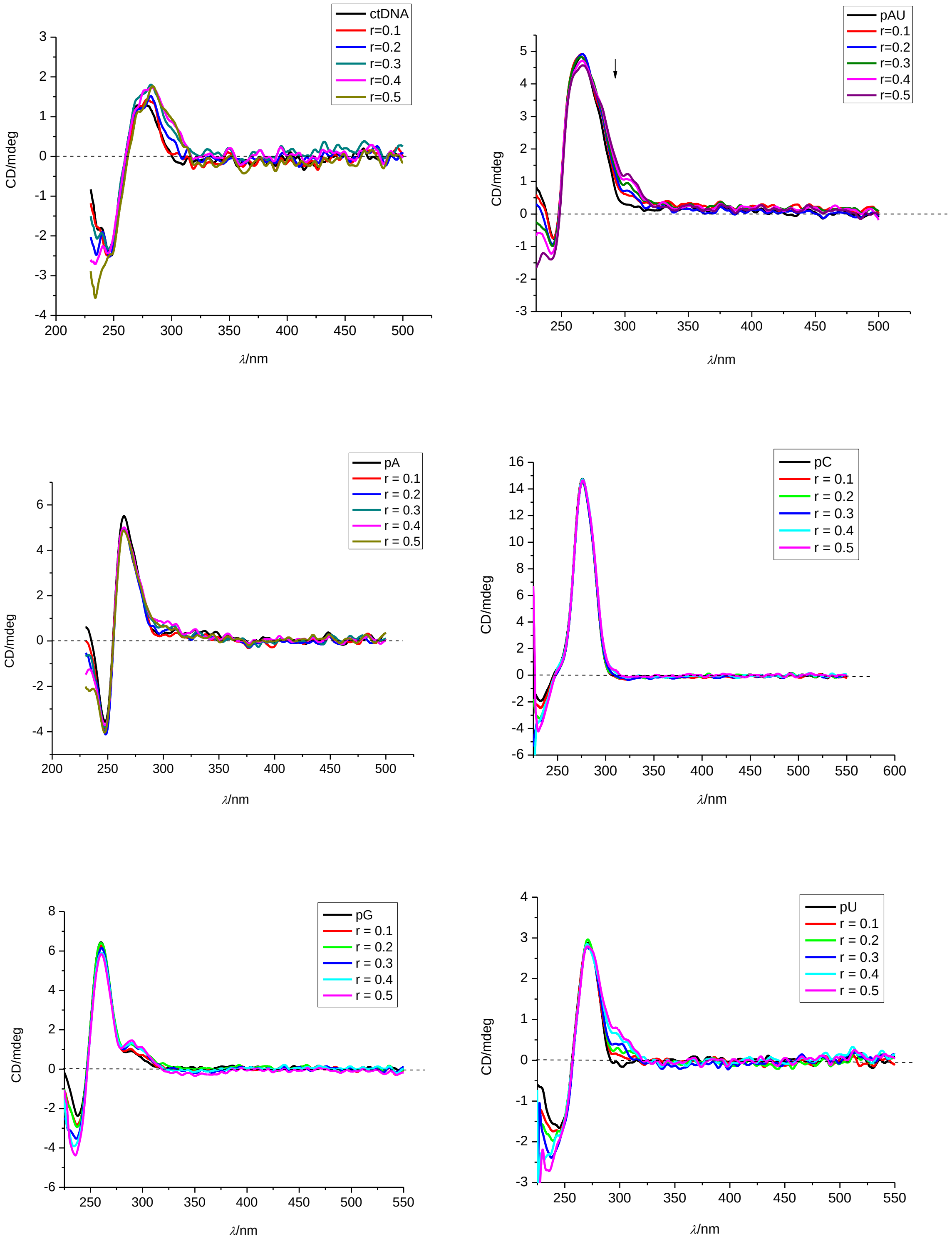

Figure S42. CD titration of **ctDNA**, **poly A – poly U**, **poly A**, **poly C**, **poly G** and **poly U** (DNA and all RNA $c = 2\times10^{-5}$ mol dm^{-3}) with **2** at molar ratios $\boldsymbol{r}_{[\mathbf{2}]\,/\,[\text{polynucleotide}]} = 0.1 - 0.5$. Done at pH 7, sodium cacodylate buffer, $I = 0.05$ mol dm^{-3}.

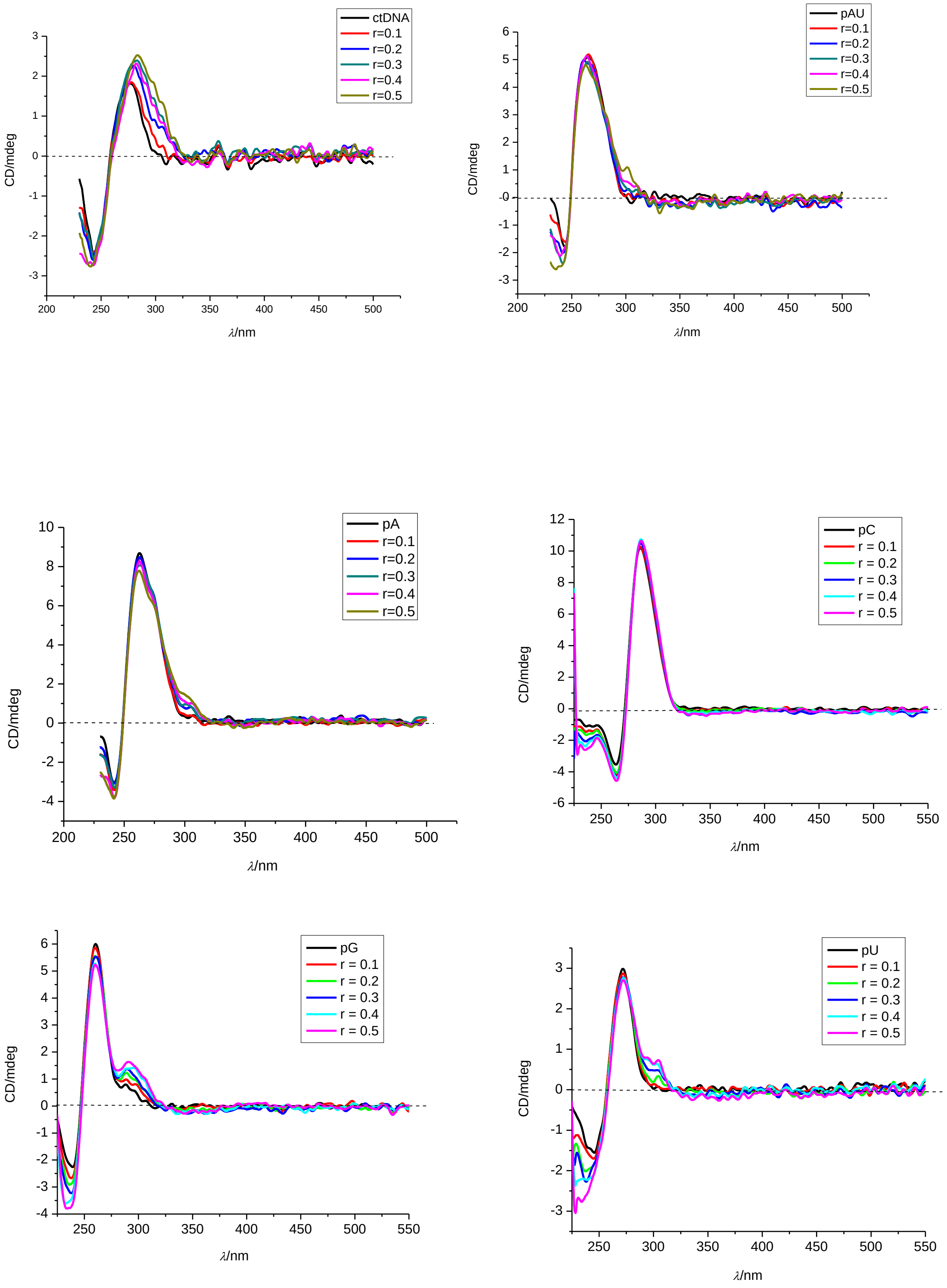

Figure S43. CD titration of **ctDNA**, **poly A – poly U**, **poly A**, **poly C**, **poly G** and **poly U** (DNA and all RNA c = 2×10^{-5} mol dm^{-3}) with **2** at molar ratios $\boldsymbol{r}$ $_{[\mathbf{2}]\,/\,[\text{polynucleotide}]}$ = 0.1 - 0.5. Done at pH 5, sodium cacodylate buffer, I = 0.05 mol dm^{-3}.

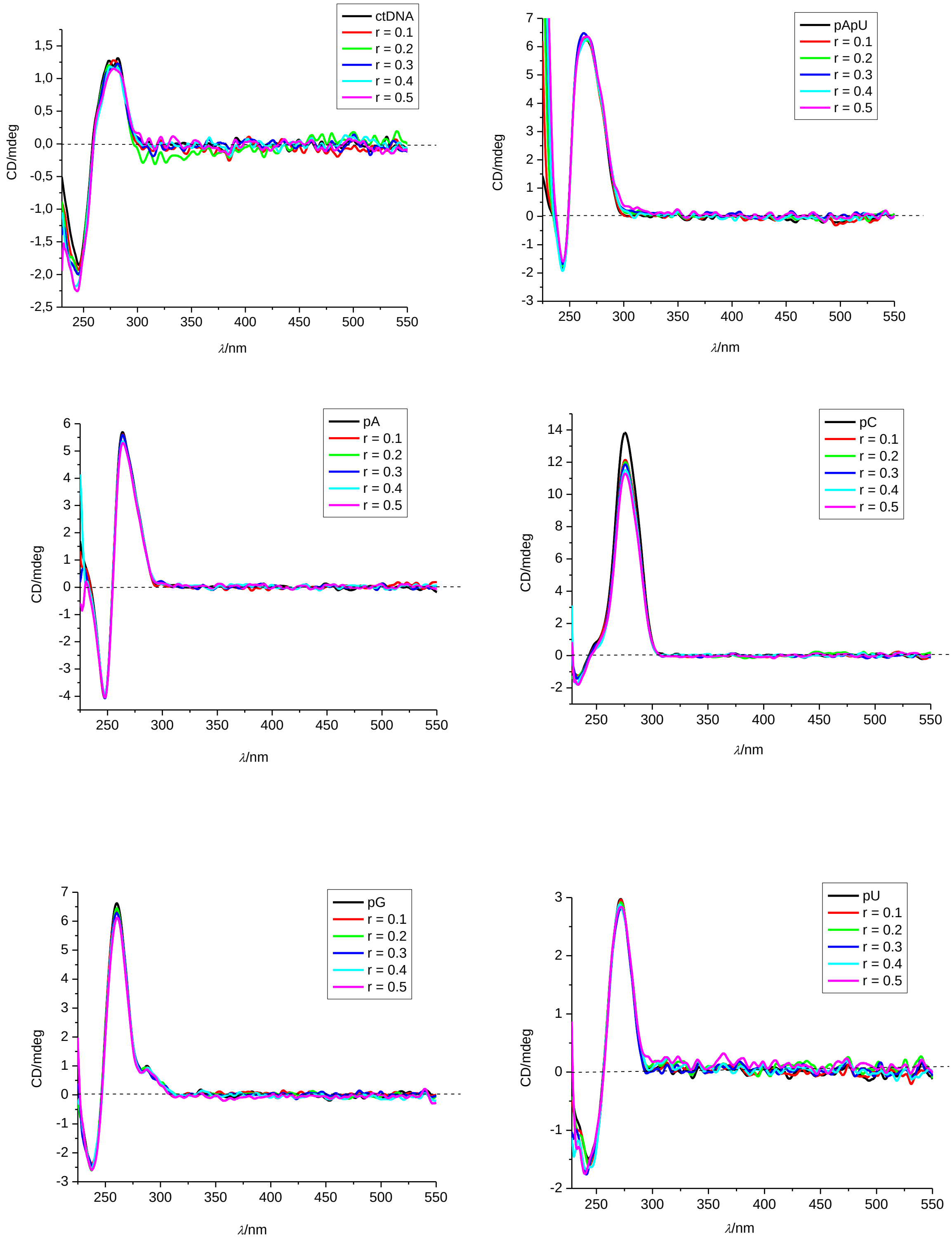

Figure S44. CD titration of **ctDNA**, **poly A – poly U**, **poly A**, **poly C**, **poly G** and **poly U** (DNA and all RNA $c = 2\times10^{-5}$ mol dm^{-3}) with **3** at molar ratios $\boldsymbol{r}_{[3]\,/\,[\mathrm{polynucleotide}]} = 0.1 - 0.5$. Done at pH 7, sodium cacodylate buffer, $I = 0.05$ mol dm^{-3}.

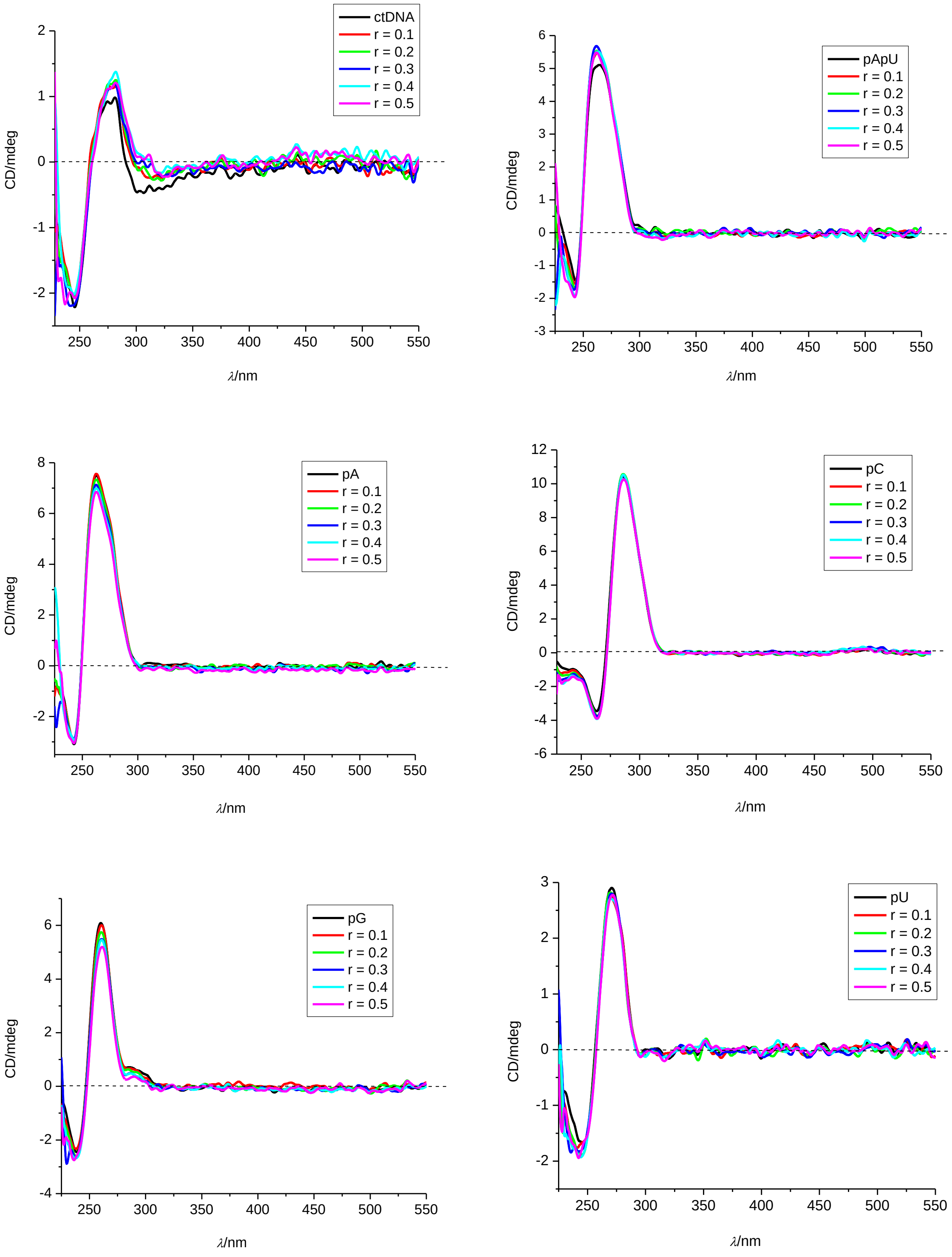

Figure S45. CD titration of **ctDNA**, **poly A – poly U**, **poly A**, **poly C**, **poly G** and **poly U** (DNA and all RNA c = 2×10^{-5} mol dm^{-3}) with **3** at molar ratios $\boldsymbol{r}_{[\mathbf{3}]\,/\,[\mathrm{polynucleotide}]}$ = 0.1 - 0.5. Done at pH 5, sodium cacodylate buffer, I = 0.05 mol dm^{-3}.

Thermal denaturation data:

Here shown are raw thermal denaturation data, all experiments are done at least twice, and for more accurate reading of thermal denaturation points 1st derivation of melting curves was used (maximum at inflection point of curve).

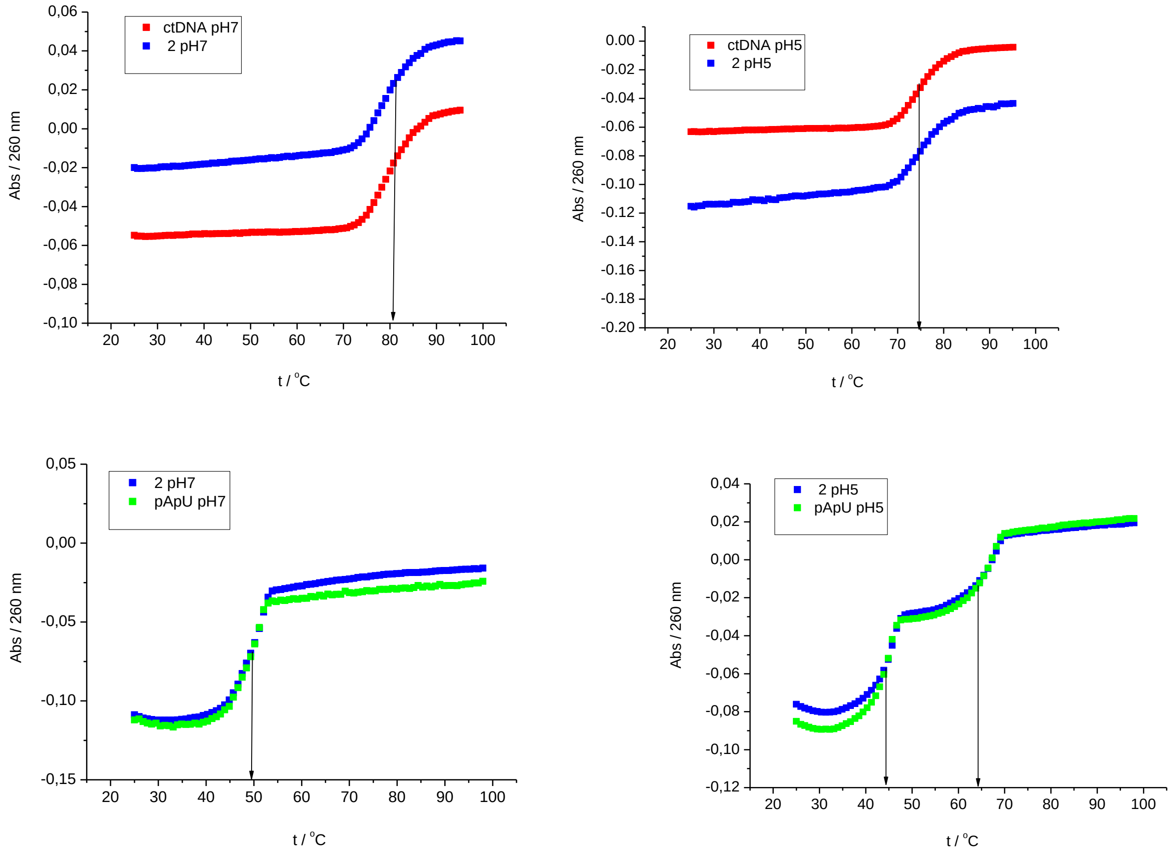

Figure S46. Thermal denaturation of **ctDNA** (UP) and **poly A - poly U** (DOWN), upon addition of **2** (r = 0.3[compound]/ [polynucleotide]) at pH 7.0 and pH 5.0 (buffer sodium cacodylate, I = 0.05 mol dm^{-3}).

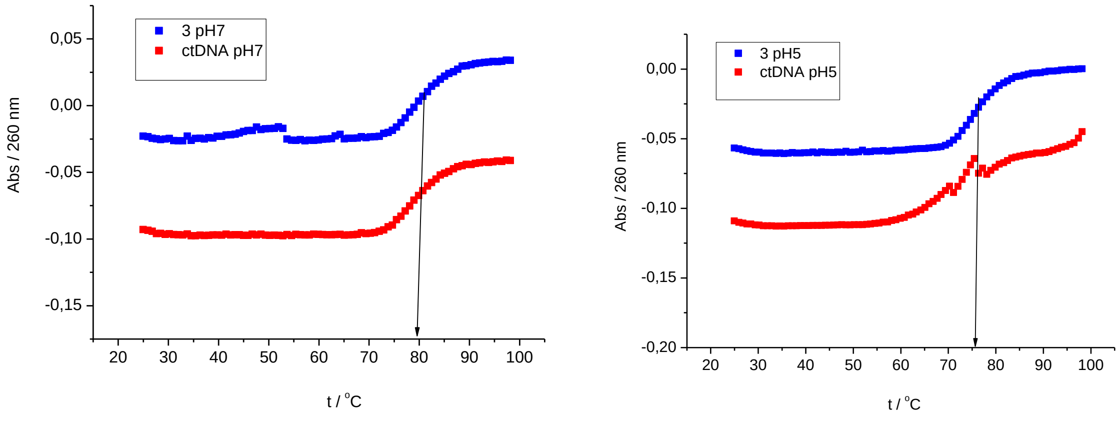

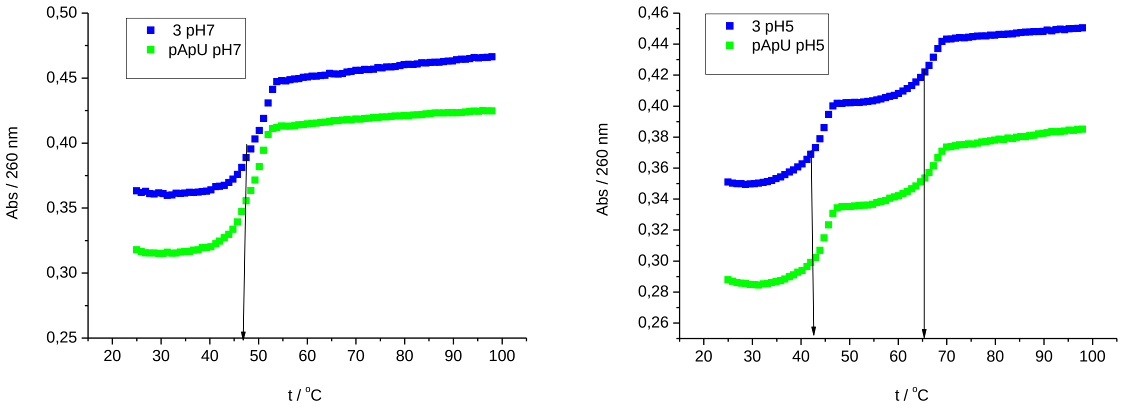

Figure S47. Thermal denaturation of **ctDNA** (UP) and **poly A - poly U** (DOWN), upon addition of **3** (*r* = 0.3[compound]/ [polynucleotide]) at pH 7.0 and pH 5.0 (buffer sodium cacodylate, *I* = 0.05 mol dm^{-3}).

Table S2.The $^{a}\Delta T_m$ values (°C) of studied ds- polynucleotides upon addition of ratio ^{b}***r*** = 0.3 of **2** and **3** at pH 7.0 and pH 5.0 (sodium cacodylate buffer, *I* = 0.05 mol dm^{-3}).

	ctDNA		polyA – polyU	
	pH 7.0	pH 5.0	pH 7.0	pH 5.0
**2**	-1.5	-0.4	0.8	0.2/0.2
**3**	-1.5	0.8	0.9	0/0

[a] Error in ΔT_m : ± 0.5°C;
[b] ***r*** = [compound] / [polynucleotide];

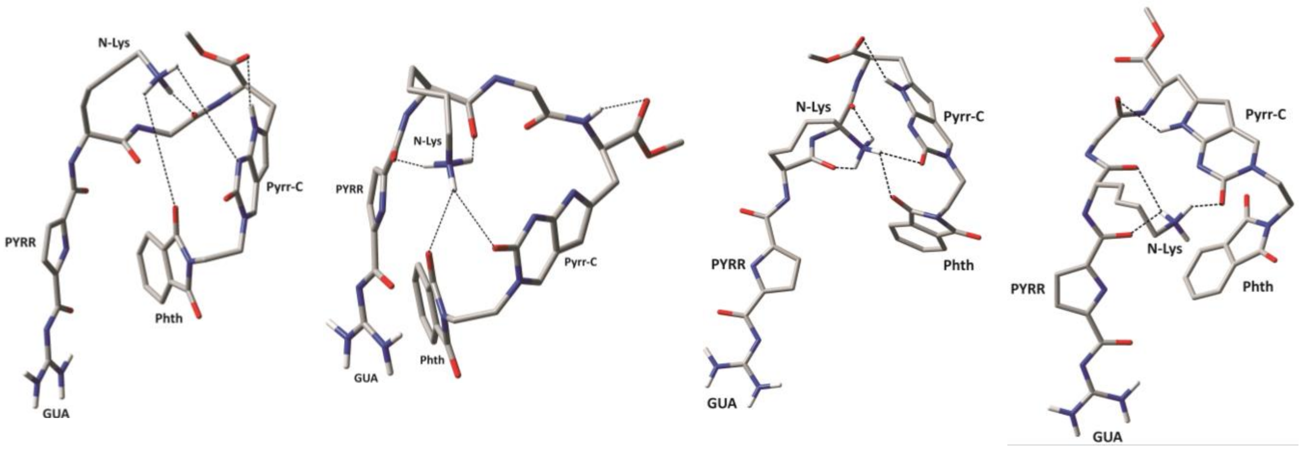

**2^+–a**	**2^+–b**	**2^+–c**	**2^+–d**
0.0	0.6	3.7	4.8

Figure S48. Most representative structures of the mono-protonated **2^+** located below a threshold of 5 kcal mol^{-1}, and their stabilities relative to the most stable conformation **2^+–a** (in kcal mol^{-1}). These are identified after a set of structures, elucidated with the clustering analysis of the corresponding MD trajectories, was optimized at the (SMD)/M06–2X/6–31G(d) level of theory. Abbreviations **GUA**, **PYRR**, **N-Lys**, **Pyrr-C** and **Phth** denote the guanidine group, pyrrole moiety, free amino group, nucleobase and phthalimide moiety, respectively. Only selected hydrogen atoms are depicted due to clarity.

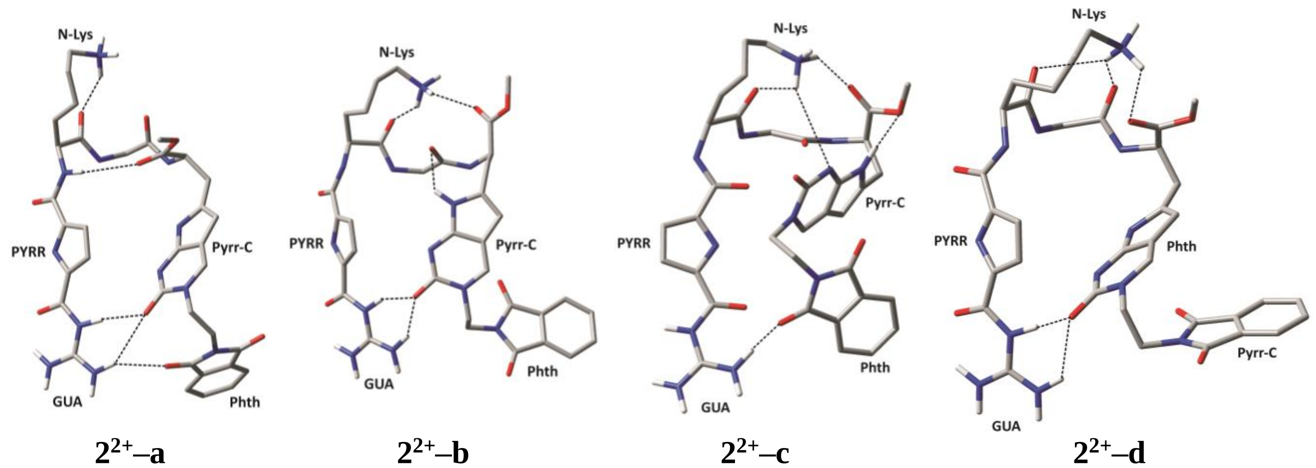

2^{2+}–a 0.0 | **2^{2+}–b** 1.6 | **2^{2+}–c** 2.0 | **2^{2+}–d** 2.2

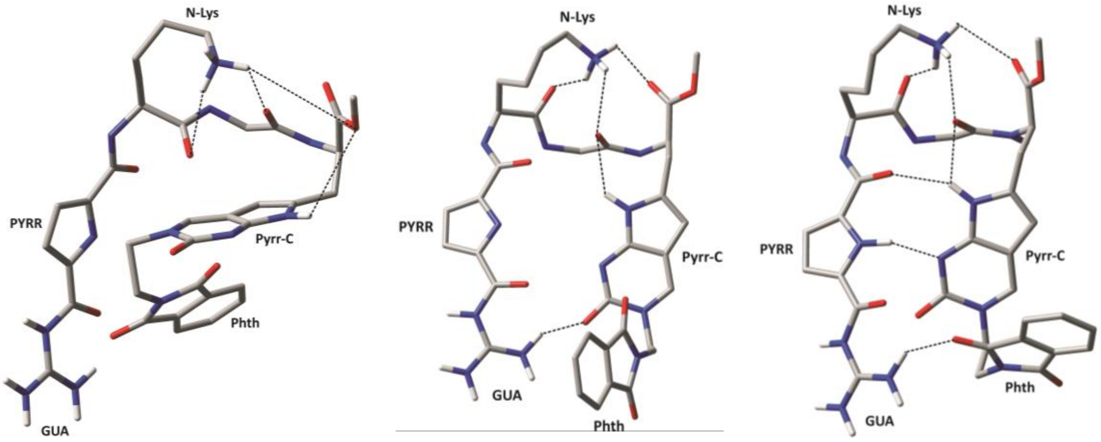

2^{2+}–e 2.9 | **2^{2+}–f** 3.0 | **2^{2+}–g** 3.4

Figure S49. Most representative structures of the di-protonated **2^{2+}** located below a threshold of 5 kcal mol^{-1}, and their stabilities relative to the most stable conformation **2^{2+}–a** (in kcal mol^{-1}). These are identified after a set of structures, elucidated with the clustering analysis of the corresponding MD trajectories, was optimized at the (SMD)/M06–2X/6–31G(d) level of theory. Abbreviations **GUA**, **PYRR**, **N-Lys**, **Pyrr-C** and **Phth** denote the guanidine group, pyrrole moiety, free amino group, nucleobase and phthalimide moiety, respectively. Only selected hydrogen atoms are depicted due to clarity.

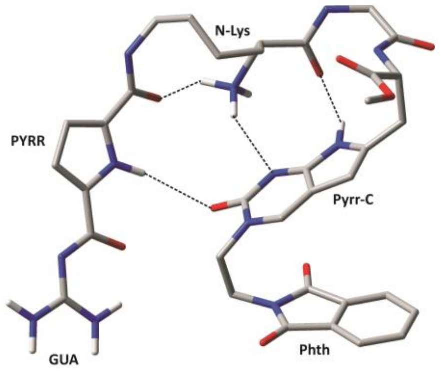

3^{+}–a
0.0

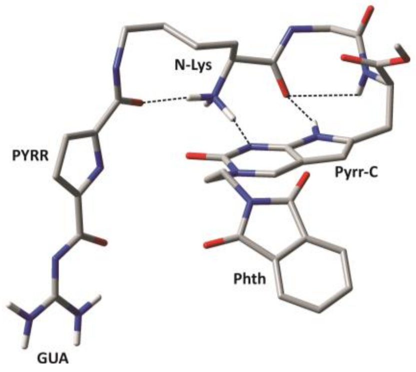

3^{+}–b
1.9

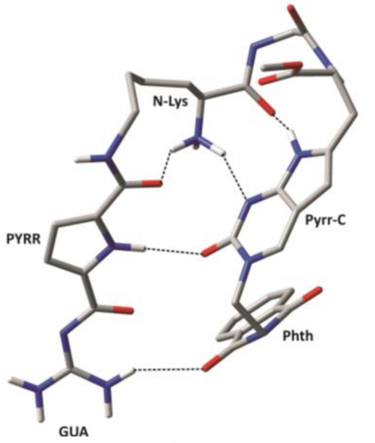

3^{+}–c
3.2

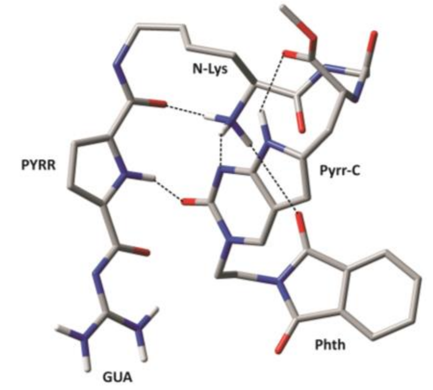

3^{+}–d
4.7

Figure S50. Most representative structures of the mono-protonated **3^{+}** located below a threshold of 5 kcal mol^{-1}, and their stabilities relative to the most stable conformation **3^{+}–a** (in kcal mol^{-1}). These are identified after a set of structures, elucidated with the clustering analysis of the corresponding MD trajectories, was optimized at the (SMD)/M06–2X/6–31G(d) level of theory. Abbreviations **GUA**, **PYRR**, **N-Lys**, **Pyrr-C** and **Phth** denote the guanidine group, pyrrole moiety, free amino group, nucleobase and phthalimide moiety, respectively. Only selected hydrogen atoms are depicted due to clarity.

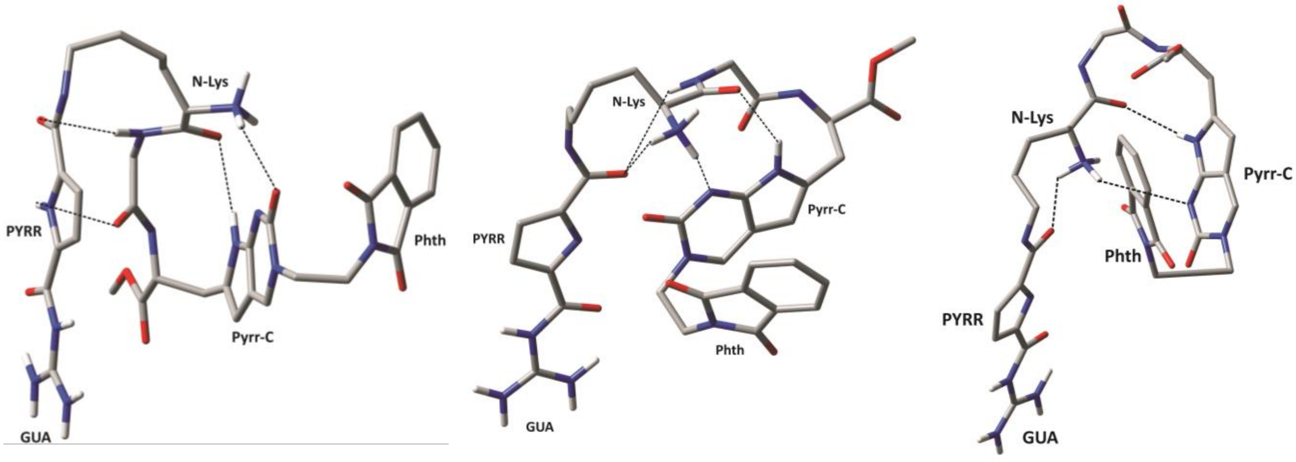

**3^{2+}–a**	**3^{2+}–b**	**3^{2+}–c**
0.0	2.4	7.3

Figure S51. Most representative structures of the di-protonated **3^{2+}** located below a threshold of 8 kcal mol^{-1}, and their stabilities relative to the most stable conformation **3^{2+}–a** (in kcal mol^{-1}). These are identified after a set of structures, elucidated with the clustering analysis of the corresponding MD trajectories, was optimized at the (SMD)/M06–2X/6–31G(d) level of theory. Abbreviations **GUA**, **PYRR**, **N-Lys**, **Pyrr-C** and **Phth** denote the guanidine group, pyrrole moiety, free amino group, nucleobase and phthalimide moiety, respectively. Only selected hydrogen atoms are depicted due to clarity.

REFERENCES

[1] J. B. Chaires, N. Dattagupta, D. M. Crothers, *Biochemistry* 1982, **21**, 3933.

[2] B. S. Palm, I. Piantanida, M. Žinić, H. J. Schneider, *J. Chem. Soc., Perkin Trans.* 2000, **2**, 385.

[3] J.-L. Mergny, L. Lacroix, *Oligonucl.*2003, **13**, 515.
